# Predicting network modules of cell cycle regulators using relative protein abundance statistics

**DOI:** 10.1186/s12918-017-0409-1

**Published:** 2017-02-28

**Authors:** Cihan Oguz, Layne T. Watson, William T. Baumann, John J. Tyson

**Affiliations:** 1 Department of Biological Sciences, Virginia Tech, Blacksburg VA, 24061 USA; 2 Department of Computer Science, Virginia Tech, Blacksburg VA, 24061 USA; 3 Department of Mathematics, Virginia Tech, Blacksburg VA, 24061 USA; 4 Department of Aerospace and Ocean Engineering, Virginia Tech, Blacksburg VA, 24061 USA; 5 Department of Electrical and Computer Engineering, Virginia Tech, Blacksburg VA, 24061 USA

**Keywords:** Parameter optimization, Differential evolution, Ensemble modeling, Machine learning, Random forests, Budding yeast, Cell cycle, Systems biology

## Abstract

**Background:**

Parameter estimation in systems biology is typically done by enforcing experimental observations through an objective function as the parameter space of a model is explored by numerical simulations. Past studies have shown that one usually finds a set of “feasible” parameter vectors that fit the available experimental data equally well, and that these alternative vectors can make different predictions under novel experimental conditions. In this study, we characterize the feasible region of a complex model of the budding yeast cell cycle under a large set of discrete experimental constraints in order to test whether the statistical features of relative protein abundance predictions are influenced by the topology of the cell cycle regulatory network.

**Results:**

Using differential evolution, we generate an ensemble of feasible parameter vectors that reproduce the phenotypes (viable or inviable) of wild-type yeast cells and 110 mutant strains. We use this ensemble to predict the phenotypes of 129 mutant strains for which experimental data is not available. We identify 86 novel mutants that are predicted to be viable and then rank the cell cycle proteins in terms of their contributions to cumulative variability of relative protein abundance predictions. Proteins involved in “regulation of cell size” and “regulation of G1/S transition” contribute most to predictive variability, whereas proteins involved in “positive regulation of transcription involved in exit from mitosis,” “mitotic spindle assembly checkpoint” and “negative regulation of cyclin-dependent protein kinase by cyclin degradation” contribute the least. These results suggest that the statistics of these predictions may be generating patterns specific to individual network modules (START, S/G2/M, and EXIT). To test this hypothesis, we develop random forest models for predicting the network modules of cell cycle regulators using relative abundance statistics as model inputs. Predictive performance is assessed by the areas under receiver operating characteristics curves (AUC). Our models generate an AUC range of 0.83-0.87 as opposed to randomized models with AUC values around 0.50.

**Conclusions:**

By using differential evolution and random forest modeling, we show that the model prediction statistics generate distinct network module-specific patterns within the cell cycle network.

**Electronic supplementary material:**

The online version of this article (doi:10.1186/s12918-017-0409-1) contains supplementary material, which is available to authorized users.

## Background

In systems biology research, mathematical models of sufficient predictive power allow researchers to interrogate biological systems under a wide variety of experimental conditions that may be difficult to achieve in the laboratory. Such in-silico experiments may lead to discoveries that affect life in important ways, for example, in understanding the molecular basis of certain diseases and in designing drugs for their treatment [1, 2]. What makes a model reliably predictive? Before using a model for predictive purposes, it is essential to show that the model is capable of reproducing major known experimental trends. In other words, incorporation of experimental data into a model by parameter optimization is a critical first step. Due to limitations in direct experimental measurements of kinetic parameters, a common approach is to estimate all unknown model parameters by minimizing the difference between model simulations and experimental data [3]. This approach often generates a set of parameter vectors with equivalent (or comparable) performance. Such parametric uncertainty can be used to advantage by extracting information about critical and dispensable parts of a model using global sensitivity analysis or identifying the most informative future experiments. This information can be used to constrain the model’s parameters [4] or to refine the model’s structure [5].

Creating an ensemble of parameter vectors with similar (or identical) performance (with respect to a known set of experimental observations) is especially useful when one would like to predict the potential outcome(s) of novel experimental designs. We refer the reader to [6] for a comprehensive survey of experimental design studies (with an emphasis on objective function formulations) from several fields including systems biology. More recent work in the area of experimental design within systems biology includes a study that compares the performances of several alternative methods with and without a predetermined network topology [7] and a novel framework for model selection implemented for both stochastic and deterministic models [8].

In the literature, “ensemble modeling” is a common term used to describe studies of multiple models [5, 9, 10] or a single model with multiple parameter vectors [11]. Here, we focus on the latter case with a complex model of the budding yeast cell cycle (more than 100 model parameters). Of special interest to us is parameter space exploration with a discontinuous objective function that is the sum of many discrete constraints. Recent work in ensemble modeling includes using simulated annealing with a multi objective function to extract robust and fragile model features [12], implementation of Metropolis Monte Carlo and multi ellipsoidal sampling [11], exploration of parameter space by adaptive sparse grids with control objectives [13], and identifying model fragilities with random walks [14]. More recently, ensembles of parameter vectors were generated to understand parameter adaptations underlying phenotypic transitions [15] with an application in pharmacological intervention [16]. In [17], Rumschinski introduced a set-based framework for detecting incorrect model hypotheses and refining parameter estimates with the help of infeasibility certificates and a bisection algorithm that identifies parts of parameter spaces consistent with incomplete and noisy experimental data. This approach was illustrated using two simple models with four species and 3–5 parameters. More recently, Rodriguez-Fernandez et al. implemented a mixed-integer nonlinear programming (MINLP) formulation to simultaneously perform model selection and parameter estimation using in silico generated data of homeostasis in *E. coli* [18]. For this biological system, the authors identified the best model among 1700 nested models in a computationally efficient manner rather than fully analysing each candidate model separately. Starting with 21 model parameters, the resulting solution showed that parameters were precisely estimated, while identifiability issues and scalability to models of larger complexity were mentioned as limitations of this model identification approach [18].

A common element in these ensemble modeling studies is the use of time-series data for optimizing parameters and for exploring the parameter space for alternative “feasible” vectors that provide acceptable fits to the data. Here, we use an ensemble modeling methodology for complex models when the constraining data are not quantitative time-series of model variables (which are often unavailable in experimental studies of cell physiology) but discrete qualitative observations (in our case, the observed phenotypes of many different yeast strains carrying mutations of cell cycle genes). In addition, the model we consider is much more complex, with many more adjustable parameters and much more experimental data, than the models studied in the work cited above.

Ensemble modeling with qualitative constraints has recently been explored by Pargett et al. [19], who combined “optimal scaling” and gradient-based multi-objective optimization for incorporating a heterogeneous set of experimental constraints into ODE models of stem cell regulation in *Drosophila*. Starting from a core model with 10 states and 18 unknown parameters, the authors generated several additional models by considering alternative connections between components of the regulatory network. Following the parameter optimization step, experimental design was implemented (based on ranking the predictive variances of measurements) in order to decrease the uncertainty of model parameter values and model structure. Each candidate model was represented by ensembles of optimal parameter vectors and Pareto optimality was used for comparing model performance and for identifying informative experiments.

In [20], temporal logics (typically used with discrete models) was implemented to express the dynamical features of a continuous (ODE-based) model of an enzymatic reaction network involved in cancer. Furthermore, global robustness and sensitivity analysis was used for identifying the boundaries between distinct regions of the model’s parameter space (producing different states such as stable steady states and oscillations) and for generating several novel biological insights regarding system’s dynamics [20].

For a recent review on the use of qualitative data for estimating the parameters of continuous models, we refer the reader to [21]. This review covers the application of alternative data normalization techniques depending on the nature of the experimental data at hand (qualitative vs. quantitative), formulation of multi-objective optimization using heterogeneous experimental data sets, and Pareto optimality based analysis of tradeoffs between such multiple objectives.

The proposed approach in this paper extends our recent work on parameter optimization of a complex model of the budding yeast cell cycle [22]. Starting from an ensemble of optimally performing parameter vectors, we propose several ways to explore the parameter space for more such vectors. In this search, our aim is to find parameter vectors with diverse predictions (i.e., an extended range of predictions for the phenotypes of novel genetic strains). We demonstrate that differential evolution (DE) [23], which is a metaheuristic method, can effectively find feasible parameter vectors with extended predictive ranges provided an additional feasibility criterion (in addition to the criterion of optimal model performance) is enforced so that the search does not get stuck in a small region of parameter space. We show how DE can be forced to widen the range of predictions during the search for optimal parameter vectors.

The application of DE in similar contexts include [24] in which DE is hybridized with Kalman Filter for improving the parameter estimation accuracy compared to pure DE and genetic algorithm (GA) based approaches. In [24], simple models of glycolysis and the cell cycle, with artificially generated noisy time series data, are used to demonstrate the improved performance of the hybrid approach. More recently, the 18 parameters of an ODE-based dynamic model of endocytosis are optimized with several metaheuristic methods including DE under different observability settings (complete vs. incomplete observability of system variables), multiple levels of measurement noise, and with real and artificially generated time series data [25]. In this study, DE turned out to be the best performer in terms of estimation accuracy and convergence speed while practical parameter identifiability problems suggested the need for additional experimental data to further constrain the model’s parameters. Recent studies on the use of metaheuristic methods in a wide range of science and enginnering applications are surveyed in [26] with more than 200 references (including the applications of several DE variants). An earlier review paper focuses on the application of metaheuristic methods to systems biology problems [27] including experimental design [28–30] and parameter identifiability [31–33].

**Fig. 1:**
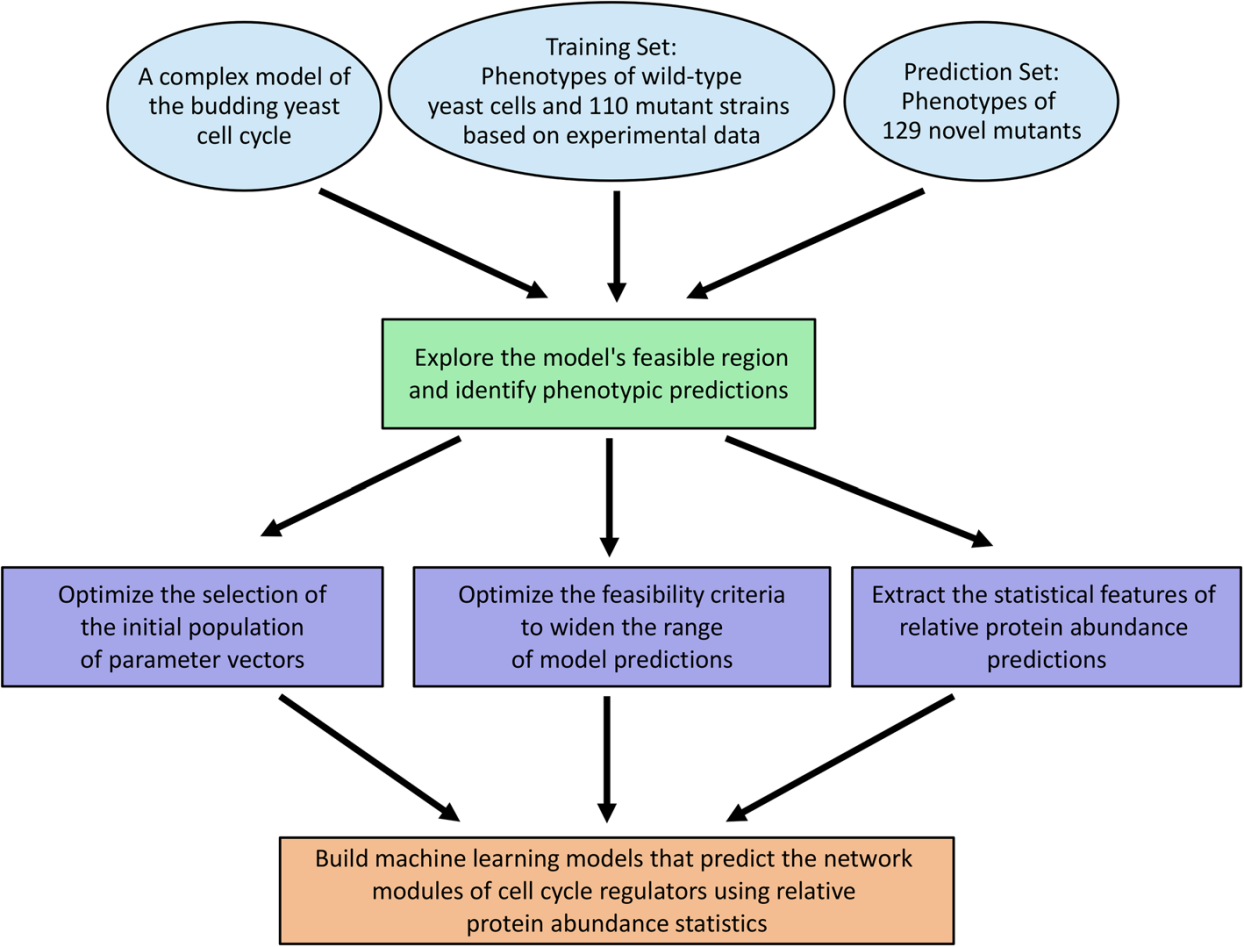
Schematic of the implemented modeling strategy for predicting the network modules of cell cycle regulators using relative protein abundance statistics

Our modified-DE approach generates an ensemble of feasible parameter vectors (i.e., vectors that satisfy a maximum number of discrete experimental constraints) with a broad “range of predictions” (i.e., vectors that extend the number of different phenotypic patterns predicted for a predefined set of mutant yeast strains). We then use this ensemble to test whether relative protein abundance predictions are influenced by the topology of the cell cycle regulatory network by ranking cell cycle regulators in our model with respect to their cumulative variability scores. The results suggest that the statistics of these predictions may be generating patterns specific to individual network modules. To test this hypothesis, we develop random forest models for predicting the network modules of cell cycle regulators using relative protein abundance statistics as model inputs. Our overall approach that ties the statistical features of model predictions to the modules of the cell cycle network, starting from optimizing the settings of DE for exploring the feasible region of the model in the parameter space is summarized in Fig. 1.

## Methods

### Problem formulation

The cell cycle is the ordered sequence of events that govern cell growth, replication of the cell’s genome, and division into two daughter cells that are capable of repeating this cycle in successive generations [34, 35]. The four phases of the cell cycle are DNA synthesis (S phase) and mitosis (M phase) separated by two gaps (G1 and G2). G1, S, G2 and M phases progress sequentially in a repeated manner, which is crucial to maintaining a constant number of chromosomes per cell after each cycle of DNA replication and cell division. Furthermore, the duration of a single cell cycle (i.e., from birth to division) has to be balanced (on average) with the time needed for doubling the amounts of all other cellular components. If this condition is not met (i.e., the mass doubling time is substantially different from the cell cycle time), then average cell size becomes progressively smaller or larger leading to cell death. In addition, a number of “checkpoints” prevent G1-S-G2-M progression in cases such as DNA damage or improper alignment of replicated chromosomes on the mitotic spindle. All of these features of cell cycle progression are controlled by the periodic activation of cyclin-dependent kinases (CDKs) [34]. Since the fundamental molecular mechanisms governing the activation of CDKs are similar among all eukaryotes, an improved understanding of cell cycle controls has potential benefits far beyond the intrinsic challenge of unraveling this complex molecular control system.

**Fig. 2:**
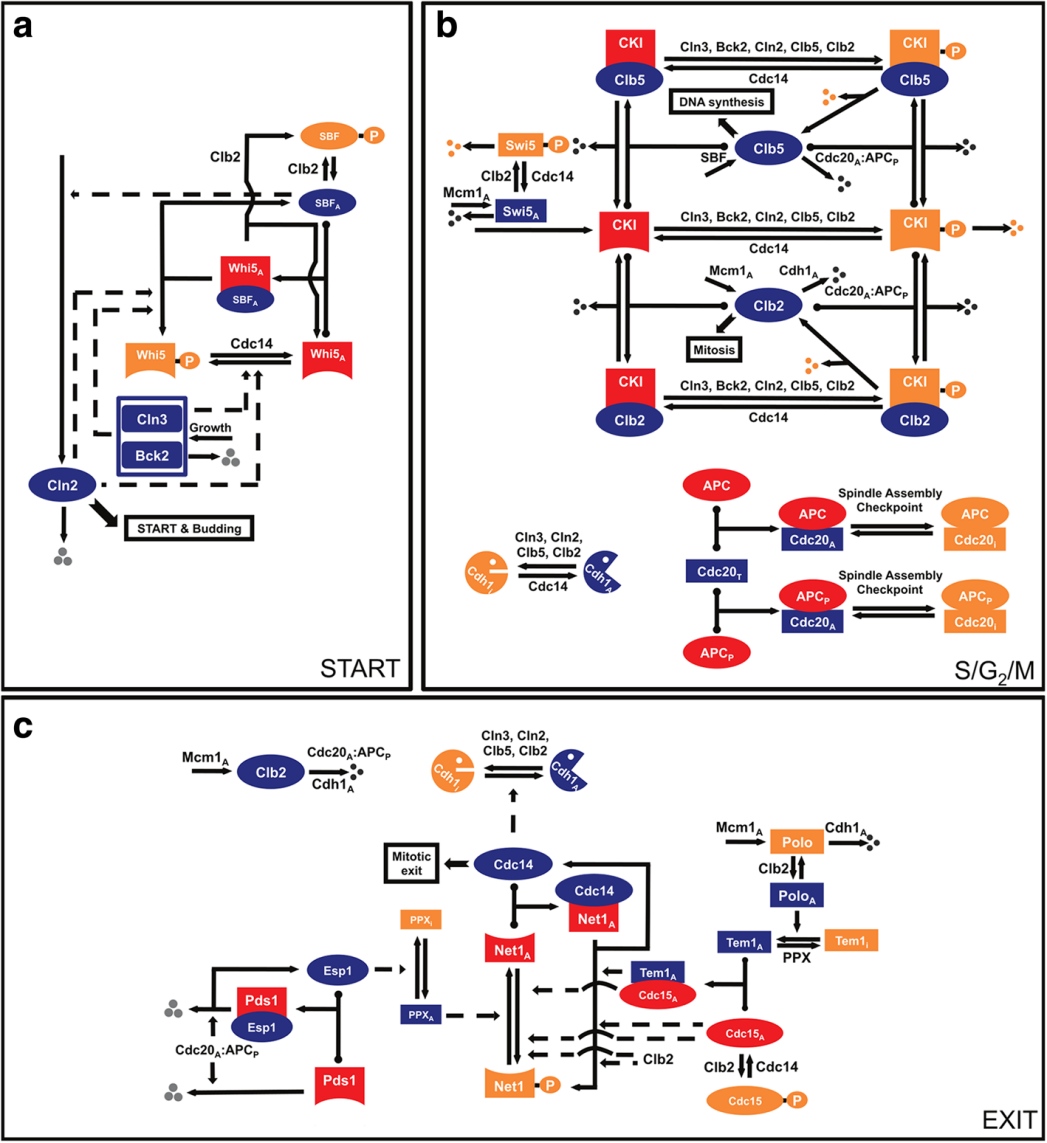
Wiring diagram of the budding yeast cell cycle network (from [40]). The network consists of three modules, namely START (in **a**), S/G2/M (in **b**) and EXIT (in **c**). *Red* and *blue* icons represent components that are in their active forms and orange icons represent components that are inactive. *Solid lines* represent chemical reactions (synthesis and degradation, phosphorylation and dephosphorylation, association and dissociation), whereas *dashed lines* represent activating or inhibitory influences of components on the chemical reactions. For simplicity, some interactions are not shown in the figures

To this end, we have proposed a variety of deterministic, stochastic and hybrid models of the CDK control mechanism in budding yeast cells and other eukaryotes [36–41]. Using the model in [40, 41], comprised of 26 ODEs and 126 kinetic parameters, we previously proposed a method for optimizing the parameter values under 119 qualitative experimental constraints [22]. (The parameters and variables of this model are listed in Additional file 1: Tables S1 and S2, respectively.) This model includes three classes of variables (or regulatory proteins). Class-1 variables are modeled by mass action kinetics of transcription factor activity and proteolytic degradation, whereas Class-2 variables (fractions of proteins in their active forms) are modeled by sigmoidal functions representing the phosphorylation and dephosphorylation reactions. On the other hand, Class-3 variables (or protein complexes) are modeled by maximum or minimum functions based on the quasi steady state assumption due to the fast time scales associated with these complex formation processes. The regulatory network represented by this model is composed of three distinct modules of proteins (START, S/G2/M, and EXIT) as shown in Fig. 2. The cell cycle events that take place in each module are summarized below.

- START module: START (or G1/S transition) is an event in G1 phase when a new round of DNA synthesis and mitosis are committed by a cell. The most critical step of the START transition is the translocation of Whi5, a stoichiometric inhibitor of SBF and MBF (transcription factors of Cln2 and Clb5 synthesis modeled as a single variable named SBF), from nucleus to cytoplasm. In early G1, SBF is not active since it is inhibited by Whi5. As the cells grow, Cln3 and Bck2 concentrations rise enough to phosphorylate Whi5 (inhibitor of SBF), and as a result SBF becomes active, promoting Cln2 and Cln5 synthesis. Increasing concentrations of Cln2, Cln3, and Clb5 support progression of bud emergence.
- S/G2/M module: Increasing Cln2 concentration following the START transition leads to phosphorylation and degradation of CKI. As a result of this, Clb5 is released. The active form of Clb5 promotes DNA synthesis, further inhibiting CKI through phosphorylation. Cln2 and Clb5 inhibit Cdh1 (responsible from Clb2 degradation) and Clb2 concentration increases resulting in the activation of Mcm1 (transcription factor of Clb2), and further Clb2 accumulation. By phosphorylating and inactivating SBF, Clb2 also halts the synthesis of Cln2 and Clb5 and the cells get ready for mitotic exit. Activation of APC by Clb2 and the cooperation of APC with Cdc20 are some of the key steps required for metaphase-anaphase transition and mitotic cyclin degradation. For Clb2 and Clb5 to be degraded, APC has to be phosphorylated and spindle assembly checkpoint needs to be released. Both of these processes are driven by Clb2.
- EXIT module: Activation of Cdc14 is the most critical event in the EXIT module since it is essential for exit from mitosis and return to G1 state. Cdc14 dephosphorylates several proteins previously phosphorylated by CDKs in S/G2/M, thereby leading to the activation of Cdh1 and CKI, as well as the repression of Clb2 and Clb5. Two pathways, namely FEAR (Cdc fourteen early anaphase release) and MEN (mitotic exit network), are involved in the activation of Cdc14. The release of Esp1 from Pds1 (through Cdc20 activity) in the FEAR pathway leads to chromatid separation and phosphorylation of Net1. As a result, Cdc14 is released from Net1:Cdc14 complex and free Cdc14 drives exit from mitosis. In order for budding yeast cells to return to G1 state by the robust phosphorylation of Net1, the FEAR pathway is supported by the MEN pathway through the activation of Cdc15 and Tem1 that form a complex (MEN). This results in the full release of Cdc14, activation of Cdh1, complete degradation of Clb2, as well as the stabilization of CKI and a fully restored G1 phase.

In [22], starting from an initial parameter vector that captured 72 of the 119 experimental phenotypes in the Training Set, we improved the number of captured phenotypes to 111. In the process, the optimization algorithm produced more than 3000 parameter vectors that captured the same 111 phenotypes of the Training Set. We call this collection an ensemble of “feasible” parameter vectors. (The ranges of model parameter values in this ensemble are given in Additional file 1: Tables S3 and S4.) In this paper, our goal is to extend the ensemble of feasible parameter vectors identified by [22] to maximize the range of model predictions for a specific group of novel mutant strains (the Prediction Set). These mutants were not included in the Training Set because their phenotypes have not yet been characterized experimentally.

Mutant strains in the Prediction Set originate from the elimination of certain phosphorylation and dephosphorylation reactions that were predicted to be critical [22] only in certain gene deletion backgrounds (not in wild type background) as shown in Table 1. We first set these rates to zero one-by-one to create nine single-mutant strains. The background is wild type (WT) for these strains. In the second step, we create double-mutant strains by setting these nine rates to zero in pairs, which results in 36 additional strains. Finally, we generate triple mutants by following the same strategy (84 more strains) resulting in a total of 129 novel strains in the Prediction Set (Additional file 1: Table S5). The initial conditions (species concentrations) for simulating these strains come from the cell state right after the last division in the WT simulations. For all simulations (listed in Additional file 1: Tables S5 and S6), Euler’s method with 0.05 min step size is used to integrate the model equations. The total simulation time per mutant (or WT) is 2000 min.

**Table 1:** Phosphorylation and dephosphorylation reactions that induce synthetic lethality upon their elimination

Eliminated reaction	Single mutation strains that are viable before and
(rate constant)	inviable after setting the rate constant to zero
Whi5 phosphorylation by Bck2 (*k* *p*_*i*5*k*2_)	*cln3 Δ*, Multicopy *BCK2*, *cdh1 Δ*, *sic1 Δ*, *swi5 Δ*,
	*CLB5-db Δ*, *net1-ts*, *GAL-CLB2*, *APC-A*
CKI phosphorylation by Cln2 (*e*_*ki*,*n*2_)	*bck2 Δ*, *GAL-SIC1*, *net1-ts*, *APC-A*
CKI phosphorylation by Clb2 (*e*_*ki*,*b*2_)	*GAL-CLN3*, *cdh1 Δ*, *GAL-CLB5*, *CLB1 clb2 Δ*
CKI dephosphorylation by Cdc14 (*k* *d* *p*_*ki*,14_)	*bck2 Δ*, *cdh1 Δ*, *GAL-CLB2*, *APC-A*
Whi5 phosphorylation by Cln3 (*k* *p*_*i*5*n*3_)	*bck2 Δ*, *cdh1 Δ*, *APC-A*
SBF phosphorylation by Clb2 (*k* *p*_*bfb*2_)	*cdh1 Δ*, *CLB5-db Δ*, *APC-A*
Whi5 phosphorylation by Cln2 (*k* *p*_*i*5*n*2_)	*bck2 Δ*, *APC-A*
Whi5 dephosphorylation by Cdc14 (*k* *d* *p*_*i*514_)	*APC-A*
Net1 dephosphorylation by PPX (*k* *d* *p*_*net*,*px*_)	Multicopy *CDC15*

Upon setting a phosphorylation or dephosphorylation rate constant to zero as specified in the left column, viability is lost in several single mutation strains (specified in the right column). These rate constants are eliminated to create the single, double, and triple mutants (a total of 129 novel mutant strains in the Prediction Set). The phenotypes and the relative abundances of species in these mutant simulations are the predictions of the model

### The range of model predictions

With *m* as the total number of feasible parameter vectors and *n* as the vector dimension (total number of parameters in the model), a collection of parameter vectors that capture the 111 phenotypes (Additional file 1: Table S7) out of the 119 total phenotypes in the Training Set defines an *m*×*n* feasible ensemble matrix.

1 $$\begin{array}{@{}rcl@{}} \mathbf{X} = \left[ {\begin{array}{cccc} {x}^{(1)}_{1} & {x}^{(1)}_{2} & \cdots & {x}^{(1)}_{n} \\ {x}^{(2)}_{1} & {x}^{(2)}_{2} & \cdots & {x}^{(2)}_{n} \\ \vdots & \vdots & \ddots & \vdots \\ {x}^{(m)}_{1} & {x}^{(m)}_{2} & \cdots & {x}^{(m)}_{n} \end{array}} \right] \end{array} $$

Here, ${x}^{(i)}_{j}$ is the value of the *j*th parameter in the *i*th parameter vector of **X**, which also generates an *m*×*l* prediction matrix.

2 $$\begin{array}{@{}rcl@{}} \mathbf{P} = \left[{\begin{array}{cccc} {p}^{(1)}_{1} & {p}^{(1)}_{2} & \cdots & {p}^{(1)}_{l} \\ {p}^{(2)}_{1} & {p}^{(2)}_{2} & \cdots & {p}^{(2)}_{l} \\ \vdots & \vdots & \ddots & \vdots \\ {p}^{{(m)}_{1}} & {p}^{{(m)}_{2}} & \cdots & {p}^{{(m)}_{l}} \end{array}} \right], \end{array} $$

where $ {p}^{(i)}_{j}\in \left \{ {0, 1, 2}\right \}$ characterizes the phenotype for the *j*th novel genetic strain for the *i*th parameter vector and *l* is the total number of novel strains. Phenotype values are set according to the following rules. If, during the simulation of a novel strain, cell size exceeds 25 (arbitrary units) at any time, then the strain’s phenotype is inviable (${p}^{(i)}_{j}=2$). On the other hand, if cell size at last division is within 5% of the cell sizes at the two previous divisions, then the phenotype is viable (${p}^{(i)}_{j}=1$). Finally, if the model generates cycles of multiple periodicity and cell size at division oscillates between values that differ by more than 5%, then the phenotype is “multiply periodic” (${p}^{(i)}_{j}=0$). The number *S*(**P**) of unique rows in **P** is defined as the range of the prediction vectors in **P**. As we explore different schemes for computing prediction matrices, we compute *S* values for the ensembles created by these schemes. For each ensemble generation scheme, the sampling efficiency (*e*_*S*_) is computed as *S*/*n*_*tot*_, where *n*_*tot*_ is the total number of samples taken from the parameter space. This measure allows us to compare different ensemble generation schemes based on the ranges of phenotypic predictions they produce.

Based on our previous study [22], which demonstrated that DE is an effective tool for exploring the parameter space of our high dimensional model given a discrete multi objective function (i.e, the number of phenotypes in the Training Set captured by the model), we continue using DE, this time for identifying the range of model predictions. While searching for an implementation of DE to meet this objective with efficient sampling, we encounter technical limitations with the standard implementation of DE that is typically used for parameter optimization, and we surmount these limitations by (i) improving the selection of the ensemble that serves as the starting point of DE, and (ii) adding new constraints to DE that force the method to search for feasible parameter vectors expanding the range of model predictions.

### Differential Evolution

Let *E* ^*D*^ denote real *D*-dimensional Euclidean space, and let *x*=(*x*_1_, …, *x*_*D*_)∈*E* ^*D*^ be a vector of parameter values. The vector *x* includes both the 126 kinetic constants in the model and the 26 ODE initial conditions (*D*=152). For each vector *x*∈*E* ^*D*^ proposed by the optimization algorithm, we calculate the phenotype $ {p}^{(i)}_{j}\in \left \{ {0, 1, 2}\right \}$ (for the *j*th strain for the *i*th parameter vector) for each of the 119 yeast strains in the Training Set. The objective function *O*(*x*) is an integer-valued function that counts the number of phenotypes in the Training Set that are correctly captured by the model, given the parameter values in the vector *x*.

In DE, parameter vectors are propagated from generation to generation by processes of mutation, crossover, and selection. Each generation (indexed by *t*=0,1,…) consists of *N* parameter vectors *x* ^(*t*,*i*)^. Hence, the real number ${x}^{(t,i)}_{j}$ is the value of the *j*th parameter in the *i*th parent in the *t*th generation. Let *u* ^(*t*,*i*)^ be the trial parameter vector born from the *i*th parent in the *t*th generation, whose components are constructed in two steps called “mutation” and “crossover”. Then, given the parent parameter vector *x* ^(*t*,*i*)^ and trial parameter vector *u* ^(*t*,*i*)^, a decision is made as to which one is propagated to generation *t*+1.

The steps of DE are described below.

1. Mutation. First, for each *i*, 1≤*i*≤*N*, we create a “mutant” vector
  3 $$ v^{(t,i)}=x^{(t,i)}+F\cdot d^{(t,i)}=x^{(t,i)}+F\cdot \left(x^{(t,i')}-x^{(t,i^{\prime\prime})}\right)  $$
  by perturbing a parental parameter vector *x* ^(*t*,*i*)^, where the perturbation vector *d* ^(*t*,*i*)^ is the difference between the parameter vectors of two distinct additional parents *i* ^′^ and *i* ^″^ chosen at random from the *t*th generation of parents, and 0<*F*<1 (*F*=0.1 in this study).
2. Crossover. For each *i* (1≤*i*≤*N*) and *j* (1≤*j*≤*D*), and uniform [ 0,1] random variables *U*_*i*,*j*_, define the offspring by
  4 $$\begin{array}{@{}rcl@{}} {u}^{{(t,i)}_{j}}= \left\{ \begin{array}{rl} {v}^{(t,i)}_{j} &, {0} \leq {U}_{i,j} \leq {C}, \\ {x}^{(t,i)}_{j} &\text{, otherwise.} \end{array} \right. \end{array} $$
  We choose the “crossover probability” *C*=0.5 so that neither parental values nor mutant values are given an advantage during the crossover step.
3. Selection. The next generation parent *x* ^(*t*+1,*i*)^ is either the parent *x* ^(*t*,*i*)^ or the trial vector *u* ^(*t*,*i*)^. As DE explores the parameter space under different settings in this study, depending on the settings of the particular DE run, we impose three distinct feasibility criteria for selection, which are described below.
  Feasibility Criterion 1 (*F* *C*_1_): Trial vector *u* ^(*t*,*i*)^ satisfies *F* *C*_1_ if the model it defines captures the 111 phenotypes listed in Additional file 1: Table S7 out of the 119 phenotypes in the Training Set. *F* *C*_1_ is always enforced by DE for creating Ensembles 1 through 16 in Table 2. For each ensemble generation scheme, the efficiency of sampling in terms of identifying parameter vectors that satisfy *F* *C*_1_ ($\phantom {\dot {i}\!}e_{{FC}_{1}}$) is computed as $n_{{FC}_{1}}/n_{tot}\phantom {\dot {i}\!}$, where $\phantom {\dot {i}\!}n_{{FC}_{1}}$ is the number of parameter vectors that satisfy *F* *C*_1_ and *n*_*tot*_ is the total number of samples taken from the parameter space.

**Table 2:** Ensembles of feasible vectors generated with different schemes

Ensemble	Scheme	Ensemble	Selection of	Feasibility criteria	# generations	#	*S*
#	#	size	the initial DE	used in selection	per DE run	DE runs	(Range of
			population from	step of DE			predictions)
			Ensemble 1				
1		3146	-	Parameter vectors	Ensemble generated	-	30
				satisfy *F* *C*_1_	in optimization [22]		
2	1	243	-	Parameter vectors	Ensemble extracted	-	51
				satisfy *F* *C*_1_	from 50,000		
					LHS samples		
3	2	7143	Randomly selected	*F* *C*_1_	400	1	6
			parameter vectors				
4	3	1893	*V*_*max*_(10)	*F* *C*_1_	400	1	41
5	4	1594	*V*_*max*_(10)	*F* *C*_1_ and *F* *C*_2_	1600	1	64
6	4	1326	*V*_*max*_(10)	*F* *C*_1_ and *F* *C*_2_	1600	1	69
7	5	3405	*V*_*max*_(123)	*F* *C*_1_ and *F* *C*_2_	1600	1	94
8	5	3753	*V*_*max*_(123)	*F* *C*_1_ and *F* *C*_2_	1600	1	80
9	6	2207	*S*_*max*_ & *V*_*max*_(123)	*F* *C*_1_ and *F* *C*_2_	1600	1	117
10	6	1842	*S*_*max*_ & *V*_*max*_(123)	*F* *C*_1_ and *F* *C*_2_	1600	1	95
11	7	3704	*S*_*max*_ & *V*_*max*_(123)	*F* *C*_1_, *F* *C*_2_, and *F* *C*_3_	1600	1	112
12	7	3481	*S*_*max*_ & *V*_*max*_(123)	*F* *C*_1_, *F* *C*_2_, and *F* *C*_3_	1600	1	133
13	8	4280	*S*_*max*_ & *V*_*max*_(123)	*F* *C*_1_ and *F* *C*_3_	1600	1	313
14	8	4550	*S*_*max*_ & *V*_*max*_(123)	*F* *C*_1_ and *F* *C*_3_	1600	1	367
15	7	15520	*S*_*max*_ & *V*_*max*_(123)	*F* *C*_1_, *F* *C*_2_, and *F* *C*_3_	2200	4	293
16	8	15050	*S*_*max*_ & *V*_*max*_(123)	*F* *C*_1_ and *F* *C*_3_	2200	4	671

Parameter ranges used for LHS are from Ensemble 1. Parameter vectors in all ensembles capture the phenotypes listed in Additional file 1: Table S7, while missing the phenotypes in Additional file 1: Table S8. *S*: The range of the phenotypic prediction vectors generated per ensemble (unique rows of the prediction matrix **P**). *V*_*max*_(10): Biased selection is used to expand the estimated volume spanned by the initial population with respect to the axes of the ten most critical parameters (Table 3). *V*_*max*_(123): Biased selection is used to expand the estimated volume spanned by the initial population with respect to the axes of 123 kinetic parameters. *S*_*max*_: Biased selection is used to enhance the initial population’s range of phenotypic predictions. The prediction ranges for all ensembles can be reproduced using Additional file 4 (simulation code), and Additional files 5, 6, 7, 8 and 9 (Ensembles 1 through 16)
  Feasibility Criterion 2 (*F* *C*_2_): *F* *C*_2_ requires that trial vector *u* ^(*t*,*i*)^ can only replace parent vector *x* ^(*t*,*i*)^ if *u* ^(*t*,*i*)^ leads to an expansion in the feasible region’s estimated volume. For this, we compute the estimated volumes of two Ensembles *X*_1_ and *X*_2_. The first ensemble *X*_1_ consists of all the parent vectors of the current *t*th generation of DE (all satisfying *F* *C*_1_) including *x* ^(*t*,*i*)^. This ensemble excludes *u* ^(*t*,*i*)^ since it is not a parent vector. The second ensemble *X*_2_ includes *u* ^(*t*,*i*)^ in addition to all the parent vectors excluding *x* ^(*t*,*i*)^. *F* *C*_2_ dictates that the trial vector *u* ^(*t*,*i*)^ can only replace *x* ^(*t*,*i*)^ if the estimated volume of the second ensemble is greater than the estimated volume of the first one (*V*(*X*_2_)>*V*(*X*_1_)). (We describe our approach for estimating the volume spanned by an ensemble of parameter vectors in Section 1 of the Additional file 2: Supplementary Text.) With ensemble creation Schemes 4 to 7 in Table 2, DE enforces *F* *C*_2_ together with *F* *C*_1_ so that a trial vector replaces the corresponding parent if and only if the trial vector that reproduces the 111 target phenotypes of the Training Set, and leads to an expansion in the feasible region’s estimated volume.
  Feasibility Criterion 3 (*F* *C*_3_): *F* *C*_3_ requires that trial vector *u* ^(*t*,*i*)^ can only replace parent vector *x* ^(*t*,*i*)^ if *u* ^(*t*,*i*)^ yields a prediction vector for the 129 mutant strains of the Prediction Set that has not been derived from any parent vector up through the *t*th generation of DE. In other words, if a trial vector *u* ^(*t*,*i*)^ satisfies *F* *C*_1_, *u* ^(*t*,*i*)^ replaces its parent *x* ^(*t*,*i*)^ if and only if the prediction vector $\hat {\mathbf {p}}$ generated by *u* ^(*t*,*i*)^ is not among the rows of the prediction matrix generated by all the parent vectors up through the point of generation of *u* ^(*t*,*i*)^. For creating Ensembles 11, 12, and 15 in Table 2, DE enforced all three criteria so that a trial vector replaces the corresponding parent if and only if the trial vector defines a model that captures the 111 target phenotypes of the Training Set, leads to an expansion in the feasible region’s estimated volume, and produces a new phenotypic prediction vector for the 129 novel mutants in the Prediction Set. Ensembles 13, 14, and 16 are created by enforcing only first and the third criteria.

## Results and discussion

### Exploring the parameter space with Latin hypercube sampling

Our starting ensemble in this study is derived from the 3415 feasible parameter vectors identified in [22]. The size of this ensemble is reduced by 8% since only 3146 of these vectors are *F* *C*_1_-feasible when truncated to 32-bit IEEE single precision. (We are eliminating parameter vectors that are very sensitive with respect to *F* *C*_1_.) We call this collection of vectors “Ensemble 1”. (Throughout this paper, parameter vectors are considered feasible only if their truncated 32-bit values are also feasible.) Applying Ensemble 1 to the Prediction Set, we generate 30 unique prediction vectors.

We explore this initial feasible region by Latin hypercube sampling (LHS). The bounds of the hypercube are formed by the minimum and maximum values of each parameter from Ensemble 1. 50,000 samples are generated as described in Section 2 of the Additional file 2: Supplementary Text. Out of these sample vectors, only 243 (0.5% of the total) are *F* *C*_1_-feasible. These feasible vectors form Ensemble 2, which produces 51 unique prediction vectors; a 70% improvement (51/30) in the total range of predictions (previously defined as the number of unique prediction vectors).

### Exploring the parameter space with DE

The results of LHS point out the possibility of finding feasible parameter vectors with a wider range of model predictions compared to those of Ensemble 1. We next investigate the possibility of using DE to identify alternative feasible ensembles with wider prediction ranges.

First, we created an initial random selection of 19 parameter vectors from Ensemble 1. (The population size of 19 is dictated by computational limitations imposed by the complexity of the model and the size of the Training Set [22]). Starting from this initial population of parameter vectors, DE explores the parameter space with mutation, crossover, and selection operations (described in Methods). (Rather than maximizing the total number of captured phenotypes by the model as we did previously [22], we only look for parameter vectors that capture the the 111 phenotypes listed in Additional file 1: Table S7 while missing the remaining eight phenotypes (Additional file 1: Table S8). Such vectors are feasible according to *F* *C*_1_ as described earlier). In 400 generations, DE generates 7143 vectors (Ensemble 3 in Table 2) whose truncated 32-bit values satisfy *F* *C*_1_. Despite its large size, Ensemble 3 yields only six unique prediction vectors for the 129 strains in the Prediction Set.

Why did DE perform so poorly compared to LHS even though, in our previous study, it was superior to random sampling in optimizing model performance (capturing phenotypes in the Training Set)? The answer comes from a comparison of the volumes the parameter space that are spanned by Ensembles 2 and 3. Ensemble 3 has an estimated volume that is 83 orders of magnitude smaller than that of Ensemble 2. In other words, DE zooms into a much smaller region of parameter space than LHS.

Following this observation, we conjectured that selecting the volume covered by the initial population of DE in a systematic way, rather than a random way, might improve the performance of the search. Therefore, we next choose an initial DE population such that the estimated volume spanned by the population vectors is maximized with respect to the axes of the ten most critical model parameters listed in Table 3. The details of the procedure for picking such a population are described in the Additional file 2: Supplementary Text (Section 3). A DE run for 400 generations, starting with this new initial population, finds 1893 feasible vectors (Ensemble 4), which account for 41 unique prediction vectors. This six-fold improvement compared to Ensemble 3 (6 vs. 41) shows that the outcome is highly dependent on the selection of the initial population, and supports the proposed scheme for maximizing the volume of the initial population of parameter vectors. We also note that Ensemble 4, although four-fold smaller than Ensemble 3 in terms of the total number of feasible parameter vectors, generates a much wider range of predictions.

**Table 3:** The ten most critical model parameters

Parameter name
Total amount of Cdc14
SPN synthesis rate
Total amount of Esp1
Total amount of Net1
Degradation rate of Cdc20
PPX inactivation by Esp1
Efficiency of Cdc14-Net1 complex (RENT) formation
Time scale for protein activation
Net1 phosphorylation by Clb2
Total amount of Mcm1

Based on the sensitivity analysis in [22], the listed model parameters had the largest effects on the objective function (number of phenotypes captured by the model) upon perturbations. Criticality decreases from top to bottom

**Fig. 3:**
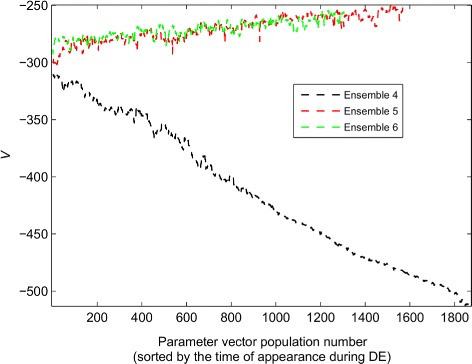
Dynamic evolution of the estimated volume *V* spanned by the parameter vectors generated during different DE realizations. Details regarding the computation of the estimated volume are provided in Section 4 of the Additional file 2: Supplementary Text. Ensembles 5 and 6 are generated by the Scheme 4 that uses *F* *C*_1_ and *F* *C*_2_, whereas Ensemble 4 is generated by Scheme 3 which only uses *F* *C*_1_ as its feasibility criteria

Nonetheless, the range of the predictions generated by Ensemble 4 is less than the range generated by Ensemble 2 (LHS). Why is this the case? The answer lies in the evolution of the volume spanned by the trial vectors generated during DE. Figure 3 (black line for Ensemble 4) shows that as DE progresses, the estimated volume spanned by the most recent feasible vectors, which serve as the parent vectors producing trial vectors in DE, continually shrinks as the generations pass. Details of the computation of this dynamic estimated volume are in Section 4 of the Additional file 2: Supplementary Text. One way to prevent this shrinkage is to increase the value of *F* in Eq. 3. However, increasing the value of *F* from 0.1 to 1 leads to a 37–64 fold drop in the sampling efficiency $e_{{FC}_{1}}\phantom {\dot {i}\!}$ with Schemes 2 and 3 (both schemes described in Table 2).

Therefore, to prevent this drop in dynamic volume, we introduce a new constraint (*F* *C*_2_) as described in the Methods section. To enforce *F* *C*_2_, the estimated volumes of two distinct ensembles are computed every time a new trial vector that satisfies *F* *C*_1_ is found. The first ensemble includes all parameter vectors satisfying *F* *C*_1_ until that point of DE, except the newest trial vector generated. Hence, this ensemble includes the trial vector’s competitor: the parent vector. The second ensemble is generated by including the trial vector instead of the parent vector, with the remaining members being identical to those of the first ensemble. If the estimated volume of the second ensemble is greater than that of the first one, the trial vector replaces the parent vector in the next generation of DE in the search for feasible vectors. Otherwise, the parent vector is not replaced, but the trial vector is recorded since it satisfies *F* *C*_1_ and its predictions for the phenotypes of the Prediction Set are evaluated after DE is complete. Succinctly, *F* *C*_2_ allows a trial vector to replace a parent only if it leads to an expansion of the feasible region. As shown in Fig. 3 (green and red lines), this new feasibility criterion prevents the volume of the feasible region from shrinking as the generations pass (two independent DE runs). Additional file 3: Figure S1 (blue line) and Additional file 1: Table S9 show that without this volume maximization strategy, the ranges of nearly all parameters are diminished after 400 generations. On the other hand, with estimated volume maximization, the majority of the parameters have about a 10% variation after 400 generations (green line in Additional file 3: Figure S1). These parameter ranges are calculated by dividing the maximum parameter values by the minimum parameter values among all parent vectors at the 400^*th*^ generation. Due to this improvement in the parameter ranges, we allow DE to explore for an additional 1200 generations. Additional file 3: Figure S1 (red line) shows that about 10% range for most parameters is still preserved among the parent vectors in the 1600^*th*^ generation. We perform two realizations of DE with this approach for 1600 generations, thereby creating two more ensembles (Ensembles 5 and 6 in Table 2). These ensembles generate 64 and 69 unique phenotypic prediction vectors, respectively.

A further improvement comes from selecting the initial DE population to maximize the volume spanned by the vectors with respect to all 123 kinetic parameters rather than just the 10 most critical parameters. (Note that the kinetic parameters *k* *s*_*n*2_, *f*, and MDT have fixed values in Additional file 1: Table S3.) Two independent DE realizations for 1600 generations produce an average of 87 unique phenotypic prediction vectors (Ensembles 7 and 8 in Table 2) further increasing the range of predictions compared to those of Ensembles 5 and 6. This is also a significant improvement over LHS (51 unique prediction vectors) even though DE required about 30,000 samples (1600 generations × 19 vectors) to identify ∼70*%* wider (87/50) range of predictions compared to 50,000 LH samples. Having gotten DE to a point where it is more efficient than random sampling in terms of exploring the feasible region, we next seek ways to improve the performance of DE even further.

### Increasing the phenotypic diversity of the initial population of DE

As we previously stated, 3146 feasible parameter vectors from the initial DE optimization run on the Training Set [22] (Ensemble 1) generate 30 unique phenotypic prediction vectors for the Prediction Set. Interestingly, 97% of these vectors generate only five of the total 30 prediction vectors as shown in Additional file 3: Figure S2. Due to this, the initial population of parameter vectors used in the last two DE runs (Scheme 5 in Table 2) produces a total of four unique prediction vectors all of which are in this set of five dominant prediction vectors. In other words, the diversity of the initial population in terms of phenotypic predictions is very low, only 13% (4 /30) of the diversity is utilized. Therefore, to increase this diversity, we select the initial population of feasible parameter vectors such that each one generates a different prediction vector (a total of 19) for the 129 strains in the Prediction Set. While using this initial selection scheme, we also maximize the estimated volume spanned by the selected vectors (Scheme 6 in Table 2). The details of this diversification procedure are in the Additional file 2: Supplementary Text (Section 5). This strategy further expands the range of predictions, with two independent runs (each for 1600 generations) increasing the average number of unique prediction vectors from 87 to 106 (Table 2). Thus, improved predictive diversity among the parent parameter vectors in the initial population results in feasible vectors (generated during DE) that are predictively more diverse.

### Enforcing an increased range of predictions during DE

In order to explore the phenotypic prediction space of the model further, we enforce a third criterion during DE. With this new criterion, a parent parameter vector is only replaced by a trial vector if the trial vector generates a new prediction vector, one not heretofore generated by any feasible parameter vector during this DE run. (For the descriptions of parent and trial parameter vectors, refer to Methods section.) In other words, with this modification, the trial parameter vector has to satisfy three constraints to replace the parent vector. It should reproduce 111 phenotypes in Additional file 1: Table S7 (*F* *C*_1_), increase the estimated volume of the feasible region upon replacing the parent vector (*F* *C*_2_), and generate a new prediction vector (*F* *C*_3_). Two independent realizations with this new scheme (for 1600 generations) increase the average number of unique predictions from 106 to 122.5 (average of Ensembles 11 and 12 in Table 2). We note that since the occurrence of a trial vector that satisfies the first two criteria is not very frequent (less than 10% among the samples generated by DE), simulating the 129 mutant strains of the Prediction Set on-the-fly (during DE) adds negligible computational time compared to the time required to run DE for 1600 generations with the 119 phenotypes in the Training Set.

**Fig. 4:**
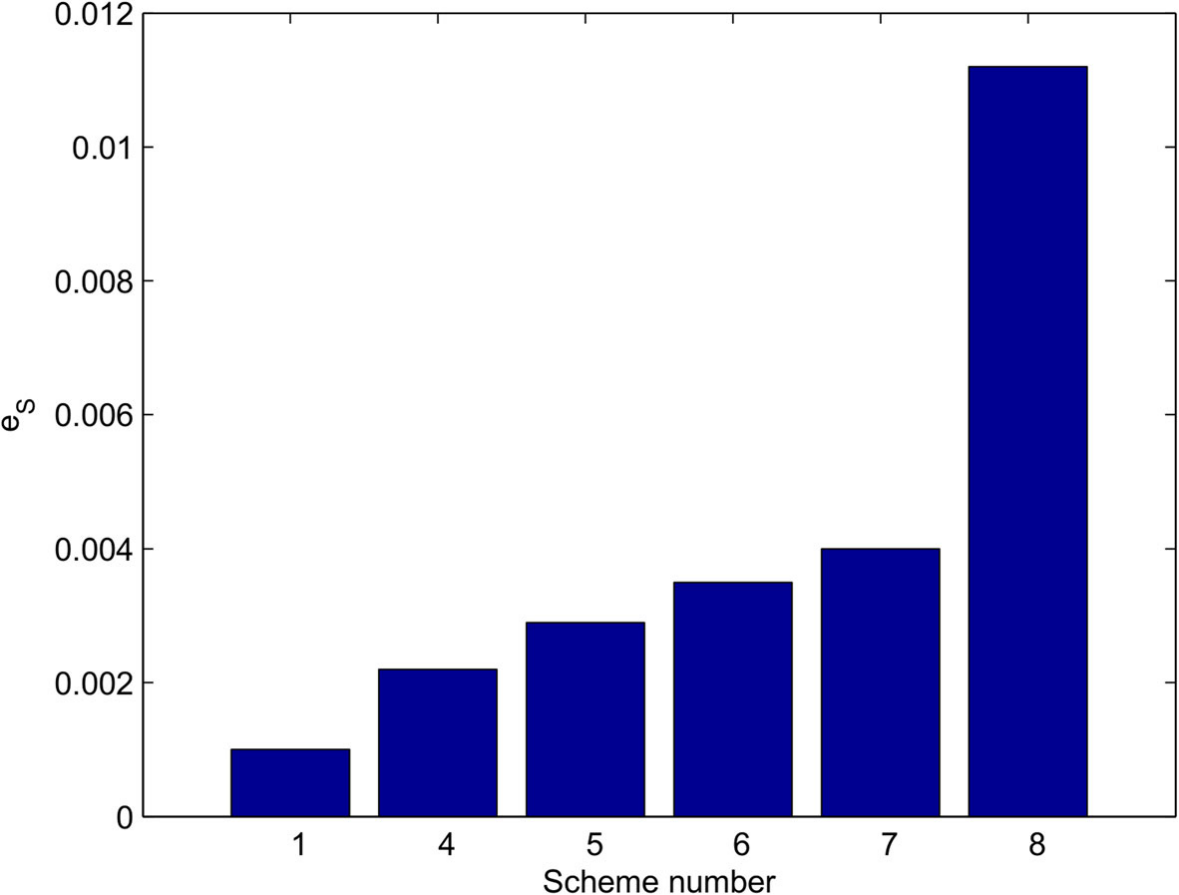
Comparison of *e*_*S*_ with different schemes. *e*_*S*_ is the efficiency of sampling computed as the ratio between the range of phenotypic predictions (*S*) and the total number of samples taken from the parameter space (*n*_*tot*_). LHS is used in Scheme 1, whereas DE is used in the remaining schemes. The detailed settings used for ensemble generation with each scheme are given in Table 2. For schemes 4–8, we average two *e*_*S*_ values computed from two independently generated ensembles (per scheme)

Since our major goal in this study is to devise a method that discovers as many unique phenotypic prediction vectors as possible, we next drop the second feasibility criterion *F* *C*_2_ (maximization of the feasible region’s estimated volume during DE) but keep the first and third criteria (*F* *C*_1_ and *F* *C*_3_). As shown in Additional file 3: Figure S3 (green line) and Additional file 1: Table S10, even though *F* *C*_2_ is dropped, DE is still able to keep some parametric variability among the feasible vectors after 1600 generations. This variability is due to the presence of *F* *C*_3_ that indirectly forces diversity in parameter values by guiding the search towards new prediction vectors. More importantly, dropping *F* *C*_2_ results in an average of 340 unique phenotypic prediction vectors (Ensembles 13 and 14 in Table 2), almost a 200% increase (122.5 to 340) in the range of predictions. Hence, not enforcing the second feasibility criterion allows us to exploit DE’s search capability for expanding the range of predictions. *e*_*S*_ value of Scheme 8, computed as the number of unique prediction vectors found per sample taken in the parameter space, is equal to 0.011 (340 unique prediction vectors found in 1600 generations × 19 parameter vectors per generation). The same efficiency value is 0.001 for random LHS (51 unique prediction vectors found in 50000 randomly generated parameter vectors), a 10-fold difference in favor of our DE based approach. Figure 4 provides a snapshot of the performances (*e*_*S*_ values) of different schemes. We also note that random selection of the initial population decreases the *e*_*S*_ value of Scheme 8 by 81%, whereas selecting an initial population with expanded volume (with respect to the axes of 123 kinetic parameters), but without enhanced predictive diversity, causes a 64% drop in Scheme 8’s *e*_*S*_ value (results based on two DE runs for 1600 generations in both cases). These results show that the selection of the initial population of DE is critical for the efficient exploration of the prediction space of the model.

**Fig. 5:**
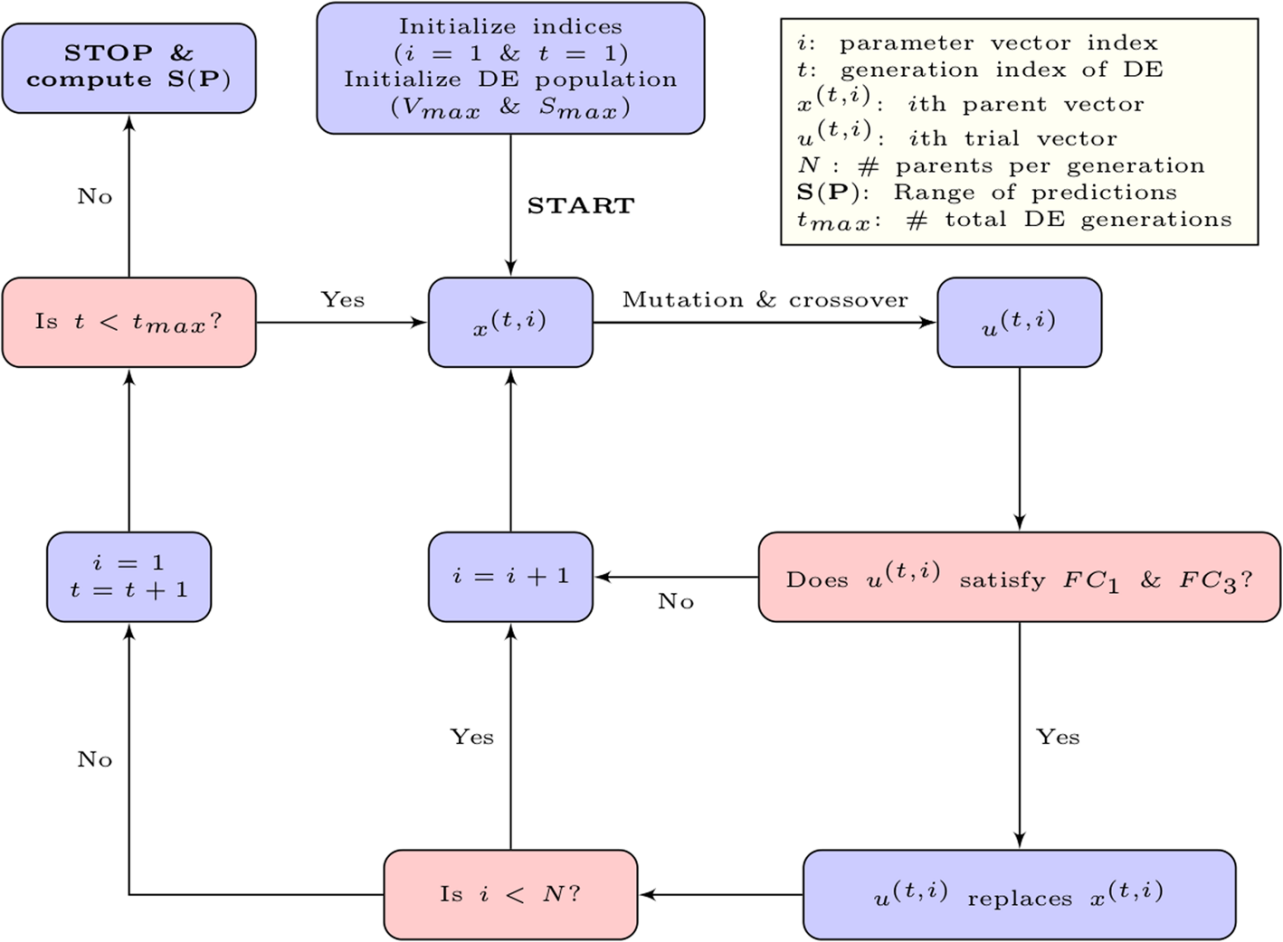
Flowchart of the parameter space exploration approach with the top-performing Scheme 8. Prediction matrix **P** is generated by simulating the novel genetic strains (the Prediction Set) in Additional file 1: Table S5 with all DE vectors that satisfy *F* *C*_1_ on the Training Set, from a total of *t*_*max*_ number of generations. The range of phenotypic predictions (*S*) is computed as the number of unique rows in **P**. With Scheme 8, the diversity of the initial population of parameter vectors is enhanced with *V*_*max*_ (biased selection for enhancing the volume spanned by the population) and *S*_*max*_ (biased selection for enhancing the range of population’s phenotypic predictions)

At this point, we have two top performing DE based schemes (Schemes 7 and 8 in Table 2) for exploring the prediction space of the model. Top performing Scheme 8 is illustrated in Fig. 5. Next, we will compare the performances of the two top performing schemes in a more thorough way, using the aggregates of ensembles from several DE runs with a higher number of generations per DE run. Then, from these ensembles, we will extract future experiments for which the model produces wide (or narrow) prediction ranges. Our goal will be to differentiate between the strong predictions of the model (e.g., novel phenotypes that are viable regardless of the parameter vector location in the feasible region) and the model predictions with some variability within the feasible region of the model’s parameter space.

### Comparison of the two most efficient ensemble generation schemes

In order to compare the performances of Schemes 7 and 8 more thoroughly, we perform four DE runs with each scheme (2200 generations per run). As shown in Table 2, Scheme 8 produces 671 unique prediction vectors (from 15050 feasible parameter vectors in Ensemble 16), whereas the number of unique prediction vectors is 293 for Scheme 7 (from 15520 feasible parameter vectors in Ensemble 15), reiterating our previously stated conclusion that Scheme 8 is more efficient in exploring the phenotypic prediction space. Lower performance of Scheme 7 suggests that maximizing the feasible estimated volume during DE (through *F* *C*_2_) may have no benefit.

However, in this section, we will show that Scheme 7 outperforms Scheme 8 in terms of a “robustness” measure based on parametric perturbations to be defined. After each of these perturbations, we simulate the model to check if the outcome of the simulation (mutant phenotype) is the same as the phenotype before the perturbation. For this robustness analysis, we limit our focus to the ten most critical model parameters (Table 3) and the ten most fragile phenotypes (Table 4), which were previously identified by the sensitivity analysis in [14].

**Table 4:** The ten most fragile phenotypes

Phenotype #	Phenotype name		
61	*CLB2-db Δ* multicopy *SIC1* (Viable)		
18	*cln1 Δ cln2 Δ cdh1 Δ* (Viable)		
63	*CLB2-db Δ clb5 Δ clb6 Δ* in galactose (Viable)		
105	*cdc15 Δ net1-ts cdh1 Δ* (Viable)		
56	*GAL-CLB2 cdh1 Δ* (Inviable)		
20	*cln1 Δ cln2 Δ cdh1 Δ GAL-CLN2* (Viable)		
59	*CLB2-db Δ* in galactose (Inviable)		
77	*APC-A* (Viable)		
78	*APC-A sic1 Δ* (Viable)		
73	*CLB5-db Δ pds1 Δ* (Viable)		

Based on the sensitivity analysis in [22], these are the phenotypes that are most often lost (i.e., incorrectly simulated) when perturbations are applied to individual model parameters in feasible parameter vectors. Fragility decreases from top to bottom

Each critical parameter is perturbed ±20%, ±40%, ±60%, ±80% (eight perturbation levels) from its nominal value and also set to zero (the ninth perturbation level). Each of these individual perturbations defines a new parameter vector. With each new vector, each of the ten fragile phenotypes is simulated (initial conditions come from the WT simulation as described before). In each simulation, the phenotype derived from the feasible vector before the perturbation is either maintained or lost. We have 900 simulations (9 perturbation levels × 10 perturbed parameters × 10 simulated phenotypes) that are used to quantify the robustness of each parameter vector. The robustness score of the *i*th parameter vector is defined by

5 $$ {{\hat{R}_{i}}=\sum\limits_{j=1}^{10}\sum\limits_{k=1}^{10}\sum\limits_{l=1}^{9} {R}_{\text{\textit{i,j,k,l}}}},  $$

where *j* is number of the critical parameter that is perturbed, *k* is number of the fragile mutant that is simulated, *l* is the number of the perturbation level, and *R*_*i,j,k,l*_ is 0 (1) if the fragile phenotype from Table 4 is lost (maintained) after the parametric perturbation. The highest robustness score within an ensemble of *m* parameter vectors is

6 $$ {{\breve{R}}=\max\limits_{1 \leq i \leq m} {\hat R_{i}}}.  $$

${\hat {R}_{i}}=900$ is the highest possible robustness score for a feasible parameter vector that satisfies *F* *C*_1_ prior to perturbations. One way to compare different ensembles in terms of robustness is to compare the distributions of ${\hat {R}_{i}}$.

In addition, each parameter vector *i* and fragile phenotype-critical parameter pair (*k*,*j*) define the robustness score

7 $$ {{\tilde {R}}_{\text{\textit{i,j,k}}}=\sum\limits_{l=1}^{9} {R}_{\text{\textit{i,j,k,l}}}},  $$

which helps us differentiate between different ensemble generation schemes listed in Table 2 in terms of the robustness linked to particular phenotype-parameter pairings. The maximum robustness of such a pair in an ensemble of parameter vectors is defined by

8 $$ {{\overline{R}}_{j,k}=\max\limits_{1 \leq i \leq m} {\tilde {R}}_{\text{\textit{i,j,k}}}}.  $$

**Fig. 6:**
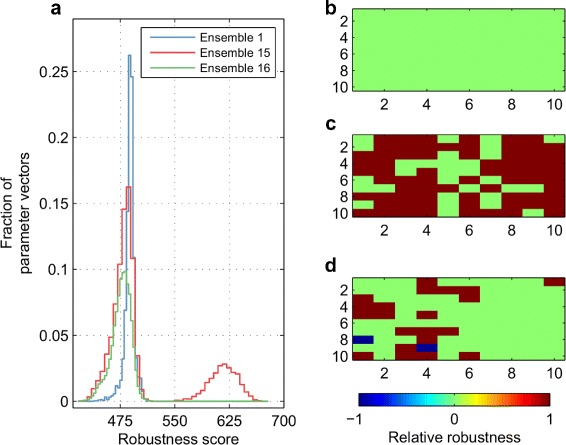
Robustness-based comparison of different ensembles. **a** Robustness score distributions of feasible parameter vectors in Ensembles 1, 15, and 16. Each parameter vector’s robustness is computed by perturbing the ten most critical model parameters with nine distinct perturbation levels to simulate the ten most fragile phenotypes (Table 4). The total number of perturbations that do not lead to phenotype losses in these 900 simulations is recorded as the robustness score $\hat R$ per feasible parameter vector. **b**-**d** Comparison of the maximum robustness ${\overline {R}}$ per phenotype-parameter pair among Ensembles 1 in **b**, 15 in **c**, and 16 in **d**. The relative robustness in Ensemble 15 or 16 is -1 (1) if the particular robustness value is lower (higher) than Ensemble 1

As shown in Fig. 6 a, feasible parameter vectors in Ensemble 15 (produced with Scheme 7) generate a bimodal distribution of robustness ${\hat {R}}$, computed for each feasible vector. Ensemble 15’s first mode with low robustness overlaps with Ensembles 1 and 16 (both ensembles have a unimodal distribution of ${\hat R}$). On the other hand, Ensemble 15’s second mode with higher robustness has no overlap with the two other ensembles’ distributions. Hence, maximizing the estimated volume of the feasible region through the mutation and crossover operations of DE leads to the discovery of feasible points in the parameter space with superior robustness. The maximum robustness value ${\breve {R}}$ among Ensemble 15 is 672, but only 512 among Ensemble 1, a 31% improvement with Ensemble 15 (generated by Scheme 7). On the other hand, ${\breve {R}}$ is 514 among Ensemble 16 (generated by Scheme 8), approximately equal to the ${\breve {R}}$ value among Ensemble 1. In addition, as depicted in Fig. 6 b, c, and d, Scheme 7 improves the maximum robustness among 70 critical parameter-fragile phenotype pairs, whereas the number of such pairs is only 21 for Scheme 8. Here, the maximum robustness (per ensemble) is quantified by a ${\overline {R}}$ value per parameter-phenotype pair (Eq. 8).

From these results, we conclude that by forcing the DE search to expand the range of predictions, one can explore the prediction space effectively as demonstrated by Scheme 8’s superior predictive diversity over its alternatives (Table 2). However, by forcing DE to maximize the feasible region’s estimated volume, it is possible to improve the robustness of the model in reproducing experimentally verified phenotypes, but at the expense of predictive diversity. Therefore, one should select the appropriate scheme for parameter space exploration, depending on one’s preference between higher robustness (Scheme 7) or diversity of model predictions (Scheme 8). Higher robustness against parametric perturbations may be enforced in some cases. For instance, one may need to modify the values of parameters in feasible vectors in order to capture additional experimental constraints while still capturing the original data [42], and this would favor the selection of Scheme 7 over Scheme 8.

### Relative protein abundance predictions

Up to this point, we have only considered the phenotypic prediction range for the 129 mutant strains in the Prediction Set. Next, we consider predictions of relative protein abundances. In simulations, the time average concentration of a protein represents the model’s prediction for that protein’s abundance in an asynchronous population of budding yeast cells. For theoretical and experimental reasons, it is better to focus on relative protein abundances, i.e., the ratio of the abundance of one protein with respect to another. Relative abundances of proteins are typically measured by Western Blotting [43] or mass spectrometry [44]. Relative abundance measurements have been useful in estimating the parameters of systems biology models in the past [45, 46].

**Fig. 7:**
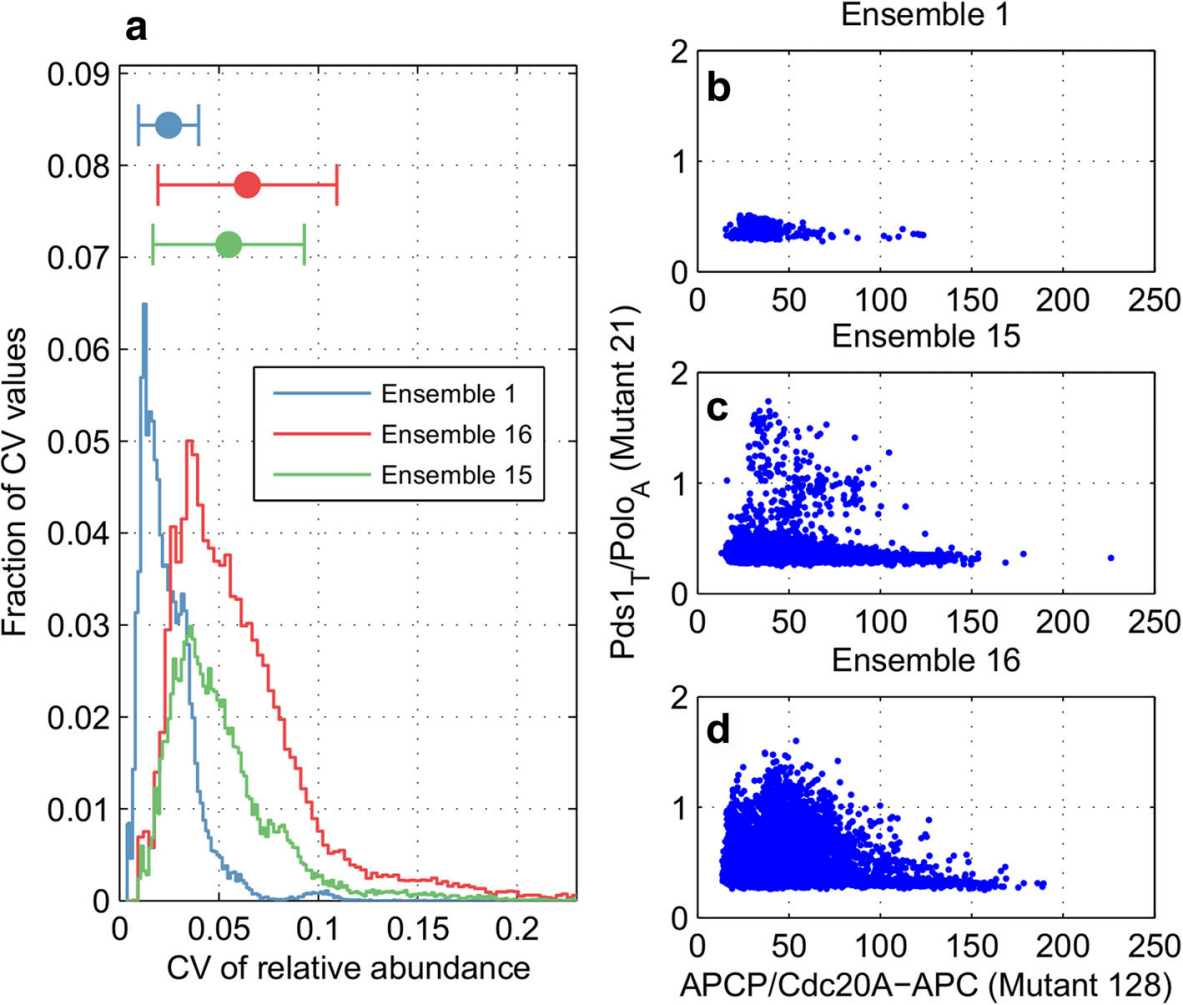
Relative abundance predictions from different ensembles. **a** Distributions of CV values of the relative abundance predictions generated by three different ensembles of parameter vectors. Mean ± standard deviation for each distribution (listed in Additional file 1: Table S11) is depicted by a single horizontal bar. The extreme values of these distributions are shown in more detail in Additional file 3: Figure S4. **b**-**d** The displayed relative abundance predictions (with high variability) are generated by Ensembles 1 (in b), 15 (in c), and 16 (in d). CV values of these predictions are of 0.18/0.51/0.53 (x-axis) and 0.076/0.27/0.41 (y-axis) among Ensemble 1/15/16

We compute the relative abundances of all species (cell size and 25 different proteins in Additional file 1: Table S2) over 2000 min in deterministic simulations of the 86 novel mutants that are consistently predicted to be viable by the parameter vectors in Ensembles 1, 15, and 16 (about 33000 feasible vectors in total). There are 91, 89, and 86 viable mutants (among the 129 strains in the Prediction Set) in these ensembles, respectively. The variability of each relative abundance prediction is quantified by its coefficient of variation (CV=standard deviation/mean) across the feasible parameter vectors within each ensemble. In order to show the effectiveness of characterizing the feasible region beyond Ensemble 1, we compare the ranges of all relative abundance predictions among the three ensembles (after collecting these CV values (one value per relative abundance) in a separate array for each ensemble). As shown in Fig. 7 a and Additional file 3: Figure S4, Ensembles 15 and 16 generated by our parameter exploration schemes 7 and 8, respectively, exhibit significantly wider CV distributions than Ensemble 1 once again demonstrating the capacity of our DE-based approach to explore the parameter space. According to Additional file 1: Table S11, both the mean and standard deviation values of CV distributions from Ensembles 15 and 16 are consistently greater than double those from Ensemble 1. Figure 7 b-d show an example, where the ranges of model predictions made by Ensembles 15 and 16 (Fig. 7 c and d) for two relative abundances are significantly wider and much less sparse in the prediction space compared to Ensemble 1 (Fig. 7 b).

**Fig. 8:**
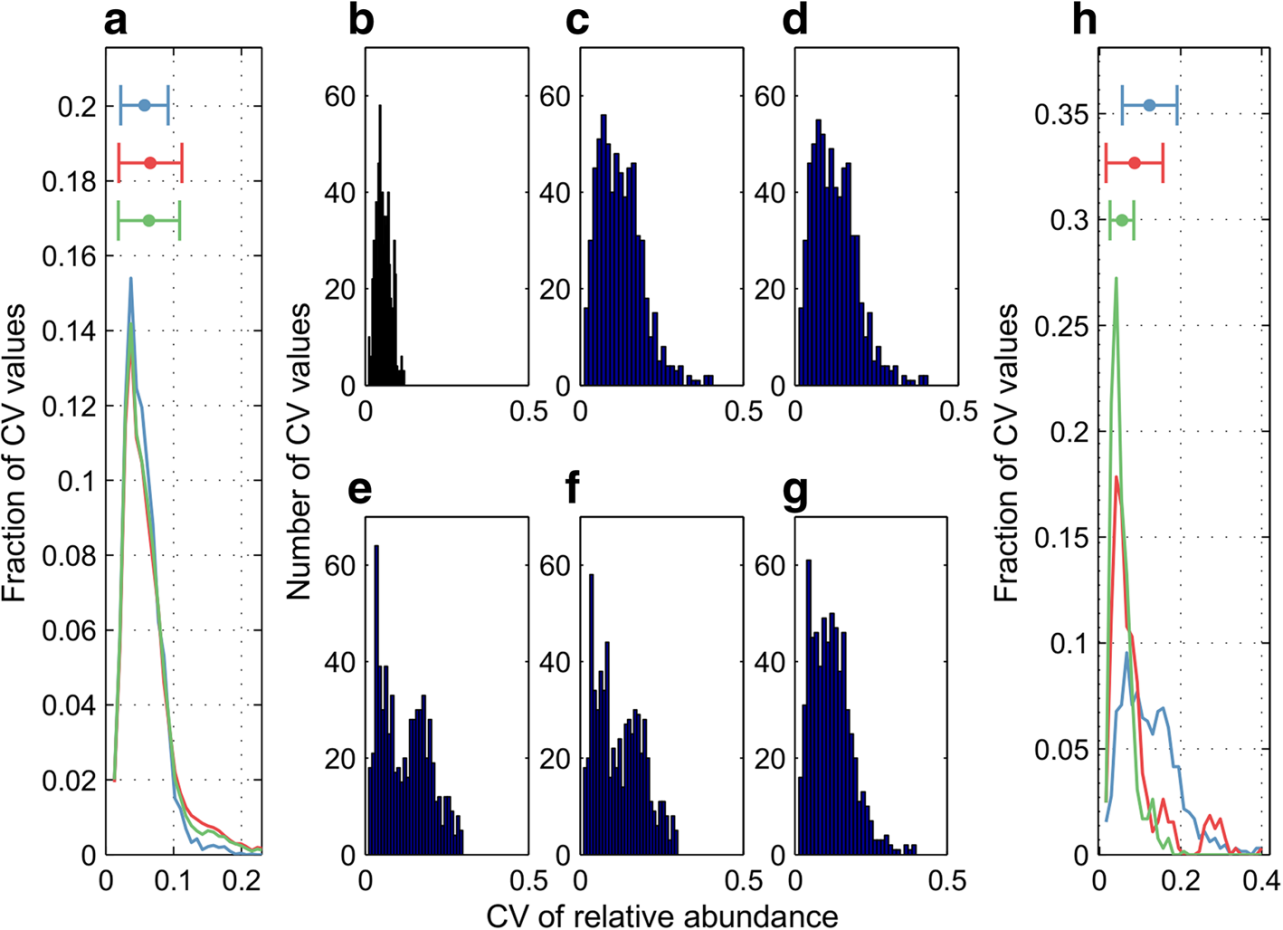
CV values of the relative abundance predictions from different mutants. **a** Smoothened distributions of CV values of the relative abundance predictions generated by single mutants (*blue curve*), double mutants (*green curve*), and triple mutants (*red curve*) among the 129 novel mutants. Mean ± standard deviation for each distribution (listed in Additional file 1: Table S12) is depicted by a single horizontal bar. These predictions are generated by the parameter vectors in Ensemble 16. **b**-**g** CV values of the relative abundance predictions for the WT strain (in **b**) and the five most informative (highest ranked based on a prediction variability statistic) novel mutants (Mutant 90 in **c**, Mutant 21 in **d**, Mutant 57 in **e**, Mutant 11 in **f**, and Mutant 85 in **g**. These predictions are generated by the parameter vectors in Ensemble 16. **h** Distributions of CV values of the relative abundance predictions generated by Mutant 21 (a double mutant shown in *blue curve*), which is a combination of two single mutants: Mutant 2 (*green curve*) and Mutant 6 (*red curve*). Mean ± standard deviation for each distribution (listed in Additional file 1: Table S13) is depicted by a single horizontal bar. These predictions are generated by the parameter vectors in Ensemble 16

As we ranked the 86 novel viable mutants in terms of decreasing value of a prediction variability statistic generated by Ensemble 16, namely the sum of relative abundance CV’s predicted for each mutant strain, we observed that the ten highest ranked strains with most variability (Table 5) are composed of three double mutants and seven triple mutants (no single mutant), suggesting that the increased number of mutations in a genetic strain provide model predictions with wider relative abundance ranges. Figure 8 a and Additional file 1: Table S12 confirm this trend. Here, we see that a higher prediction variability statistic is associated with double and triple mutants compared to single mutants. Histograms of the CV distributions for the WT strain and the five highest ranked novel mutant strains (Fig. 8 b-g) indicate that these mutants generate predictions with significantly higher variabilities compared to the WT strain. Interestingly, these five double and triple mutants have common mutations (Table 5). For instance, even though Mutant 57 has an additional mutation compared to Mutant 11, the distributions of the CV values of the predicted relative abundances are almost identical. Hence, this additional mutation does not increase the prediction variability in the relative abundance measurements. A similar trend is observed with Mutant 21 (double mutant) and Mutants 90 and 85 (triple mutants), once again indicating a common mutation pair that is responsible for the wide prediction ranges. Mutant 21 is created from two single mutations (Mutants 2 and 6 in Additional file 1: Table S5). As depicted in Fig. 8 h and Additional file 1: Table S13, these two individual mutations synergize upon creating Mutant 21 and generate overall ranges of predictions (each CV value corresponds to the range of one prediction) wider than either of the single mutations alone. These analyses highlight the usefulness of our approach to designing genetic strains that generate informative model predictions. For instance, the pair of relative abundance predictions shown in Fig. 7 d have CV values that are higher than 0.40 among Ensemble 16. In contrast, the two relative abundance predictions shown in Additional file 3: Figure S5 have CV values that are less than 0.01 among the same ensemble. Hence, the presented parameter space exploration approach enables us to differentiate between informative genetic strains with high prediction variability (Fig. 7 b, c, and d) and the genetic strains that generate model predictions with low variabilities (Additional file 3: Figure S5).

**Table 5:** The ten novel phenotypes with highest predictive variance

Mutant #	Mutation 1	Mutation 2	Mutation 3
90	*e*_*ki*,*n*2_=0	*k* *p*_*bfb*2_=0	*k* *d* *p*_*i*514_=0
21	*e*_*ki*,*n*2_=0	*k* *p*_*bfb*2_=0	-
57	*k* *p*_*i*5*k*2_=0	*e*_*ki*,*b*2_=0	*k* *d* *p*_*i*514_=0
11	*k* *p*_*i*5*k*2_=0	*e*_*ki*,*b*2_=0	-
85	*e*_*ki*,*n*2_=0	*k* *p*_*i*5*n*3_=0	*k* *p*_*bfb*2_=0
58	*k* *p*_*i*5*k*2_=0	*e*_*ki*,*b*2_=0	*k* *d* *p*_*net*,*px*_=0
81	*e*_*ki*,*n*2_=0	*k* *d* *p*_*ki*,14_=0	*k* *p*_*bfb*2_=0
128	*k* *p*_*bfb*2_=0	*k* *d* *p*_*i*514_=0	*k* *d* *p*_*net*,*px*_=0
42	*k* *p*_*bfb*2_=0	*k* *d* *p*_*net*,*px*_=0	-
106	*e*_*ki*,*b*2_=0	*k* *p*_*bfb*2_=0	*k* *d* *p*_*net*,*px*_=0

Based on the relative abundance predictions generated by the parameter vectors in Ensemble 16, these mutants mutants have the highest variability values (i.e., Mutant 90 has the largest sum of CV values from the relative abundance predictions among the 129 novel mutants). Variability decreases from top to bottom

Similar approaches have been used in two previous model driven experimental design studies [47, 48]. In [47], Dong et al. presented an experimental design process called “Computing Life” and illustrated it for the biological clock of *Neurospora crassa*. At each experimental design cycle, the authors chose the Maximally Informative Next Experiment (MINE) from a large set of potential network models and microarray experiments using a criterion that enforced maximal independence between observables. This analysis identified several genes (from a total of 11,000 genes) under the direct control of a key clock oscillator and also discovered a link between this clock and ribosome biogenesis. In [48], Donahue et al. implemented a sparse grid approximation using polynomials to explore their objective function (based on time series data) in order to discriminate simultaneously between uncertainties in model structure and in parameter values (without an initially determined feasible region). One disadvantage of the sparse grid search is the required smoothness of the objective function, whereas typically rugged objective function landscapes [49] are observed for large and nonlinear network models. This is especially the case in our study where many discrete experimental constraints determine the feasibility of model parameter vectors. For detailed theoretical discussions regarding the use of prediction variability statistics in model-based experimental design, we refer the reader to two excellent reviews [4, 6].

**Fig. 9:**
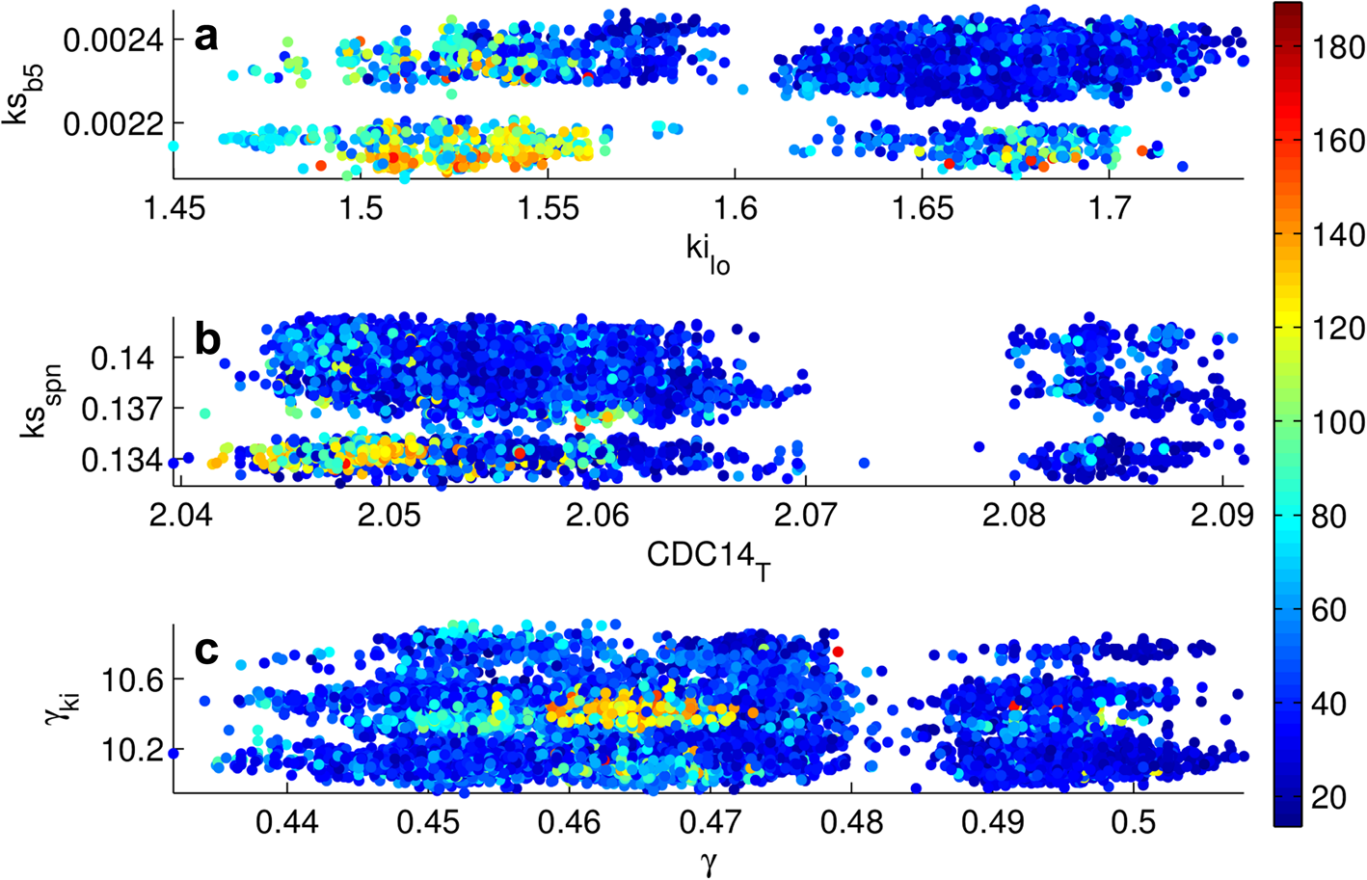
Predictions for the relative abundance of APCP with respect to Cdc20A-APC in Mutant 128 as a function of different parameter pairs. *k* *i*_10_ (basal Polo inactivation) and *k* *s*_*b*5_ (basal Clb5 synthesis rate) in **a**), *C* *D* *C*14_*T*_ (Total amount of Cdc14) and *k* *s*_*spn*_ (SPN synthesis rate) in **b**), and *γ* (time scale for protein activation) and *γ*_*ki*_ (CKI inactivation time scale) in **c**). Color map indicates the relative abundance values ranging 13–189

As shown in Fig. 9, starting from Ensemble 16, one can also refine the feasible ranges of parameters upon incorporating a relative abundance measurement into the model. Here, we see that low values of *k* *i*_10_, *C* *D* *C*14_*T*_, and *k* *s*_*spn*_, and medium values of *γ* and *γ*_*ki*_ produce high values (greater than 120) of the relative abundance APCP/Cdc20A-APC in Mutant 128 (relative abundance measurement with the highest variability based on its CV value). Hence, it is possible to use new data from an experiment that is predicted to be informative and eliminate some of the parameter vectors in the feasible ensemble. In other words, future measurements selectively taken based on model predictions by targeting highly variable relative protein abundances within particular mutants could be useful for reducing parametric uncertainty. However, we did not perform experimental design in our study since it is outside our scope.

### Ranking cell cycle proteins and biological processes in terms of prediction variability

In order to study the potential relationships between the variabilities of relative abundance predictions linked to individual cell cycle proteins and the topology of the cell cycle network, we first identified the total variability associated with each of the 26 proteins. To this end, for each protein, we computed the sum of the CV values for each protein abundance ratio with that particular protein in its numerator. We refer to this sum as the “variability score” of the protein. (We have verified that our ranking of proteins based on their variability scores does not depend on whether we use the protein in the numerator or denominator in the summation process (data not shown)).

Next, we ranked the cell cycle proteins with respect to their total variability scores (Table 6). Five of the seven species in the EXIT module were in the group of “low variability” proteins (the bottom half of the group). In contrast, four of the five proteins within the START module were within the “high variability” category (the top half). The less variable nature of the EXIT module aligns with our previous study, which identified the EXIT module as the most fragile network module [22], as well as an experimental study which showed the cell cycle was least tolerant to overexpression of *CDC14* (a major regulator in the EXIT module) among 31 cell cycle genes studied by Moriya et al. [50]. Similarly, two other proteins in the EXIT module, namely *NET1* and *PDS1* were among the more fragile genes (ranked 8^*th*^ and 10^*th*^ among 31 genes in terms of cell cycle’s tolerance limit to their overexpression) in [50] in agreement with the “low variability” status of these proteins in our model (Table 6).

**Table 6:** Cell cycle regulators ordered in terms of their variability scores (decreasing from top to bottom)

Rank	Regulator	Variability score	Percentile	Category	Network module	Class label
1	Cdc20A-APC	231.4	100	High variability	S/G2/M	2
2	Cln3	208.3	94	High variability	START	1
3	BUD	195.71	91	High variability	-	-
4	APCP	191.2	87	High variability	S/G2/M	2
5	WHI5dep	160.63	82	High variability	START	1
6	SBFdep	156.26	79	High variability	START	1
7	Polo _*A*_	154.48	75	High variability	EXIT	3
8	Cln2	152.8	71	High variability	START	1
9	Tem1	147.02	67	High variability	EXIT	3
10	ORI	146.3	64	High variability	-	-
11	Clb5 _*T*_	139.93	60	High variability	S/G2/M	2
12	CKI _*T*_	129.58	56	High variability	S/G2/M	2
13	Cdh1 _*A*_	124.2	52	High variability	S/G2/M	2
14	SPN	122.95	48	Low variability	-	-
15	Polo _*T*_	122.16	44	Low variability	EXIT	3
16	Bck2	120.28	40	Low variability	START	1
17	Pds1 _*T*_	119.01	37	Low variability	EXIT	3
18	Clb2 _*T*_	118.21	33	Low variability	S/G2/M	2
19	PPX	117.53	29	Low variability	EXIT	3
20	Cdc20A-APCP	115.58	25	Low variability	S/G2/M	2
21	V (Mass)	114.18	21	Low variability	-	-
22	CKI _*P*_	111.99	17	Low variability	S/G2/M	2
23	Cdc15	109.39	13	Low variability	EXIT	3
24	Net1dep	109.19	10	Low variability	EXIT	3
25	CDC20 _*T*_	109.03	6	Low variability	S/G2/M	2
26	Swi5 _*T*_	107.48	1	Low variability	S/G2/M	2

Ten regulators in the S/G2/M module, on the other hand, were evenly distributed among both categories. Two regulators in this module, namely Cdc20A-APC and Cdc20A-APCP had strikingly different predictive variability scores. Cdc20A-APC complex has the highest score of 231.4), whereas Cdc20A-APCP complex was ranked 20^*th*^ with a score of 115.58. These two complexes are responsible from the degradation of Clb5, Clb2, and Pds1 through ubiquitin-mediated proteolysis [51]. Interestingly, Cdc20A-APCP is 9.3, 3.8, and 6.5–fold more potent than Cdc20A-APC (based on the average parameter values in Ensemble 16) in terms of degrading Clb5, Clb2, and Pds1, respectively. Hence, a potent (or critical) regulator turned out to have less predictive variability compared to a weaker regulator in our model once again pointing to a potential relationship between the cell cycle network and the variability scores of individual model variables (more critical variables have less predictive variance). After ranking cell cycle proteins by denominator-based formation of relative abundance-network module pairs (i.e., each relative abundance is matched to the module of the protein in its denominator), we computed the Pearson correlation coefficient between the vectors formed from the order of the two rankings (numerator-based vs. denominator-based) as 0.99. Hence, the ranking of cell cycle proteins was independent of the way network modules were assigned to relative abundance values.

Next, we compiled all of the gene ontology based biological processes [52] associated with the cell cycle proteins and ranked them using the variability score associated with each regulator (Table 7). In cases where a biological process was associated with more than one protein, we computed the mean and standard deviation of the variability scores associated with each process. According to Table 7, the biological processes with the largest predictive variability values (85^*th*^-100^*th*^ percentile range), were identified as the regulation of cell size and regulation (both negative and positive) of the G1/S transition. These processes are known to be closely tied to each other [53]. Budding yeast cells that are exceptionally small at birth than others spend more time in G1 before entering S phase due to an experimentally verified size threshold requirement [54]. Later studies showed that this size control mechanism acts for the most part in daughter cells (in our simulations, the “daughter cell” is the smaller cell at each asymmetric division) through multiple daughter-specific transcription factors [55] and also showed that this mechanism is “imperfect” [56] since cell size at birth is not perfectly correlated with the length of G1 phase. It is also thought that size fluctuations can not be compensated in a single cycle due to the imperfect nature of size control [53] and we hypothesize that this factor plays into the high values of model prediction variability associated with the relative abundances of proteins that regulate size control and the G1/S transition. Aligned with this trend, Di Talia et al. [56], observed that cell size at birth is significantly variable with CV values around 0.2 for both daughters and mothers. Hence, our identification of “cell size” and “regulation of G1/S transition” as the biological processes associated with the highest values of predictive variability is consistent with previous experimental literature.

**Table 7:** Biological processes ordered in terms of predictive variability which decreases from top to bottom

Rank	Biological process	Variability	Percentile
		score	
1	Regulation of cell size	164.38 ±42.18	100
2	Negative regulation of transcription involved in	160.63 ±0.00	95
	G1/S transition of mitotic cell cycle		
3	Regulation of transcription involved in	157.89 ±71.29	91
	G1/S transition of mitotic cell cycle		
4	Positive regulation of transcription involved in	156.26 ±0.00	85
	G1/S transition of mitotic cell cycle		
5	Positive regulation of transcription from RNA polymerase II promoter	156.26 ±0.00	85
6	Regulation of cyclin-dependent protein kinase activity	154.81 ±38.41	81
7	Mitotic spindle orientation checkpoint	147.02 ±0.00	71
8	Exit from mitosis	147.02 ±0.00	71
9	Establishment of mitotic spindle localization	147.02 ±0.00	71
10	Regulation of mitotic spindle assembly	139.93 ±0.00	64
11	Positive regulation of DNA replication	139.93 ±0.00	64
12	G1/S transition of mitotic cell cycle	130.11 ±13.89	60
13	Positive regulation of spindle pole body separation	129.07 ±15.36	54
14	G2/M transition of mitotic cell cycle	129.07 ±15.36	54
15	Positive regulation of protein ubiquitination	124.20 ±0.00	47
16	Negative regulation of spindle pole body separation	124.20 ±0.00	47
17	Regulation of cell cycle	120.28 ±0.00	40
18	Positive regulation of gene expression	120.28 ±0.00	40
19	Mitotic sister chromatid segregation	119.01 ±0.00	36
20	Regulation of mitotic spindle elongation	118.21 ±0.00	30
21	Negative regulation of protein dephosphorylation	118.21 ±0.00	30
22	Positive regulation of mitotic metaphase/anaphase transition	116.62 ±10.73	23
23	Activation of APC-Cdc20 complex activity	116.62 ±10.73	23
24	Protein phosphorylation	109.39 ±0.00	16
25	Mitotic cytokinesis	109.39 ±0.00	16
26	Regulation of exit from mitosis	109.29 ±0.14	12
27	Negative regulation of cyclin-dependent protein kinase by cyclin degradation	109.03 ±0.00	6
28	Mitotic spindle assembly checkpoint	109.03 ±0.00	6
29	Positive regulation of transcription involved in exit from mitosis	107.48 ±0.00	1

Based on Table 7, the biological processes associated with the smallest predictive variability values (1 ^*st*^-12^*th*^ percentile range) were identified as the positive regulation of transcription involved in exit from mitosis (and also its simpler form “regulation of exit from mitosis”), mitotic spindle assembly checkpoint, and negative regulation of cyclin-dependent protein kinase by cyclin degradation. These processes are associated with Swi5 (the transcription factor for CKI), Net1 (stoichiometric inhibitor of Cdc14), Cdc15 (responsible for Net1 phosphorylation), and Cdc20 (required for Clb5, Clb2, and Pds1 degradation) that all play critical roles for mitotic exit which is the cell cycle network module with least predictive variability as we previously stated.

The findings that we summarize in this section, when taken together, suggest that the statistics generated from the model predictions are influenced by the topology of the cell cycle network and that these statistics may also be generating distinct patterns that are specific to individual network modules. In order to test this hypothesis, we next implemented the “random forest” classification method and developed statistical models to predict the network modules in which individual cell cycle regulators operate (i.e., biological functions of these regulators) using model prediction statistics.

### Predicting biological functions (or network modules) of cell cycle regulators using relative abundance statistics

In order to predict the biological functions (or network modules) of cell cycle regulators using relative abundance statistics, we implemented the random forest classification method using the Statistics and Machine Learning Toolbox^TM^ of Matlab^®;^ [57]. For each relative abundance (a total of 47850 relative abundances with finite CV values), four features were used for predicting the network modules of individual cell cycle proteins, namely the mean, standard deviation, and CV values of the particular relative abundance and the ID-number of the viable novel mutant (of the 129 strains in the Predictive Set plus the wild type strain) that is simulated to generate the relative abundance prediction. The true class of each relative abundance was identified as the network module to which the protein in the numerator belonged. (We later tested if the predictive accuracy significantly changed when the denominator was taken as the reference point for identifying the true class labels and found out that our predictive ability was not dependent on this choice.) Predictive accuracy is computed by generating receiver operating characteristic (ROC) curves (true positive rate vs. the false positive rate obtained using several classifier output thresholds) and quantifying the areas under these curves (AUC) for each network module as the positive class vs. the negative class generated by combining the remaining two modules (i.e., START module vs. S/G2/M and EXIT modules, S/G2/M module vs. START and EXIT modules, and EXIT module vs. START and S/G2/M modules). We performed 100 runs (per set of features or model inputs) and reported the average AUC and its *p*-value based on a Z-test with respect to a random model with two classes (i.e., AUC=0.5) [58], an approach commonly taken for computing the statistical significance of AUC in ROC based predictive modeling studies. When the *p*-value computed from the AUC is less than 0.05, the predictive performance measured by the AUC value is deemed statistically significant. We also generated randomized models by permuting the class labels (or network modules) attached to each relative abundance in 100 independent realizations. The *p*-values associated with the predictive performances of these randomized models were expected to be higher than 0.05 in order to verify the statistical significance achieved by the non-randomized models trained and tested by the true network modules associated with all the relative abundances.

Per decision tree, approximately 64% of the samples are retained to be used for model training, whereas the remaining samples are used for model testing. These test samples are referred to as “out-of-bag” (OOB) samples, whereas the training samples are expanded by bootstrapping [59] (or sampling with replacement) up to the sample size of the original data [60] prior to model training. Classification of the test samples are based on the complete ensemble of trees (a total of 100 trees) with a voting scheme. For example, a test sample (i.e., the protein in the numerator of a relative abundance) is predicted to be in the “START” module if the number of trees that predict this outcome is higher than the ones that predict the protein’s network module as “S/G2/M” or “EXIT”.

As shown in Table 8, random forest models developed using model prediction statistics were highly predictive of network modules (START, S/G2/M and EXIT) in which the cell cycle regulators operate with an average AUC of 0.83–0.87 (with less than 0.01% variability and *p*-values of zero). Furthermore, the randomized models generated by permuting the network modules attached to relative abundances had no predictive value indicated by AUC values around 0.5 (and *p*-values around 0.5), typical of a coin-flipping process with two possible system states (e.g., START module vs. S/G2/M or EXIT). Hence, the predictive performances of models trained with the correct (or non-random) network module-relative abundance matching were statistically significant.

**Table 8:** Predictive performances of the random forest models developed using relative abundance statistics along with the *p*-values corresponding to mean AUC values in 100 independent realizations (STD corresponds to standard deviation)

Positive class	AUC (Mean ±STD)	*p*-value	AUC (Mean ±STD)	*p*-value
			with randomized modules	with randomized modules
START	0.8667 ±0.0004	<1.0E- 15	0.4996 ±0.0046	0.55
S/G2/M	0.8326 ±0.0005	<1.0E-15	0.5003 ±0.0038	0.46
EXIT	0.8366 ±0.0005	<1.0E-15	0.5008 ±0.0038	0.40

Here, for each relative abundance, the network module of the cell cycle regulator in the “numerator” is used as the true class label of the relative abundance for model training and testing. The results were practically identical (less than 0.01 change in AUC values) when the regulator in the “denominator” was used as the true class label

Recent studies have indicated that abundances of proteins are regulated in a biological function-dependent manner [61–63]. For example, in general, production and degradation rates of regulatory proteins are trained by evolution to quickly respond to certain stimuli, whereas proteins produced by housekeeping genes and structural proteins that are critical for the integrity of an organism are relatively more stable [61]. Furthermore, it is now also clear that protein abundance signatures are shaped not only by transcriptional and post-transcriptional regulation [64] but also by translation and post-translational regulation, which play prominent roles in determining both dynamic and steady-state behaviours of protein abundances [61, 62, 65]. The cell cycle model used in our study takes into account all of these individual modes of regulation and successfully predicts the network modules of individual cell cycle regulators (related to their biological functions) from model prediction statistics. This outcome demonstrates the critical importance of developing comprehensive and accurate models of important biological processes (such as cell cycle control) for correctly predicting various dynamic and steady-state behaviours shaped by a complex interplay between several modes of regulation. Generating correct predictions despite such complexity holds the key to elucidating critical components and their interactions in complex biological networks in a context-dependent manner.

## Conclusions

Previously [22], we demonstrated a practical approach for fitting a complex dynamical model of the budding yeast cell cycle [40, 41] to a large set of qualitative experimental observations (viability/inviability of mutant strains of yeast). Taking a further step in this work, we characterize the feasible region of this model in order to test whether the statistical features of relative protein abundance predictions are influenced by the topology of the cell cycle regulatory network.

Using differential evolution (DE), we generate an ensemble of feasible parameter vectors that reproduce the phenotypes (viable or inviable) of wild-type yeast cells and 110 mutant strains (we call these 111 strains the Training Set). We use this ensemble to predict the phenotypes of 129 mutants (the Prediction Set) for which experimental data is not available. We identify 86 novel mutants that are predicted to be viable and then rank the cell cycle proteins in terms of their contributions to cumulative variability of relative protein abundance predictions. Of the three modules in the cell cycle control system (START, S/G2/M, and EXIT), the EXIT module (the most fragile module identified in [22]) has the least predictive variability, whereas the START module has the highest predictive variability. When we compile all of the gene ontology based biological processes associated with the cell cycle proteins in the model, we identify that the proteins involved in “regulation of cell size” and “regulation of G1/S transition” contribute most to predictive variability, whereas proteins involved in “positive regulation of transcription involved in exit from mitosis”, “mitotic spindle assembly checkpoint”, and “negative regulation of cyclin-dependent protein kinase by cyclin degradation” contribute the least. These results suggest that the statistics of these predictions may be generating patterns specific to individual network modules (START, S/G2/M, and EXIT). To test this hypothesis, we develop random forest models for predicting the network modules of cell cycle regulators using relative abundance statistics as model inputs. Predictive performance is assessed by the areas under receiver operating characteristics curves (AUC). Our models generate an AUC range of 0.83-0.87 as opposed to randomized models with AUC values around 0.50. By using differential evolution and random forest modeling, we show that the model prediction statistics generate distinct network module-specific patterns within the cell cycle network.

## Additional files

Additional file 1 Supplementary Tables. This pdf file includes 13 tables referred to in the main text. (PDF 175 kb)

Additional file 2 Supplementary Text. This pdf file includes detailed descriptions of certain aspects of our study including the computation of the estimated volume spanned by an ensemble of parameter vectors (Section 1), using LHS for generating an ensemble of parameter vectors (Section 2), selection of the initial DE population that spans a large volume (Section 3), computation of the estimated volume spanned by the most recent subensemble of parameter vectors (Section 4), selection of the initial DE population that spans a large volume and has a large prediction range (Section 5), alternative parameter space exploration methods (Section 6), the impact of precision on the number of identified feasible parameter vectors (Section 7), the impact of additional normalization on the contributions of individual parameters to the feasible region’s volume (Section 8), the impact of additional normalization on the contributions of individual parameters to the robustness score (Section 9), the impact of the viability criteria on the model prediction range (Section 10), the choice of ODE solver (Section 11), a potential biological application of the parameter space exploration approach (Section 12), and discussion regarding the most critical model parameters (Section 13), and the most fragile phenotypes (Section 14). (PDF 350 kb)

Additional file 3 Supplementary Figures. This pdf file includes five additional figures. (PDF 1640 kb)

Additional file 4 Simulation code. This ZIP file includes a Matlab script (getpredictionrange.m), a C subroutine (for solving the ODEs), and additional files that simulate the model with different ensembles (by the execution of getpredictionrange.m) to compute the prediction range and protein abundances for all mutant strains in the Prediction Set for a given ensemble (ensembles included as additional ZIP files and getpredictionrange.m currently set to load and use Ensemble 1 for the computations). (ZIP 18.7 kb)

Additional file 5 Ensemble 1. This ZIP file includes all of the parameter vectors in Ensemble 1. (ZIP 1720 kb)

Additional file 6 Ensembles 2 through 9. This ZIP file includes all of the parameter vectors in Ensembles 2 through 9. (ZIP 12100 kb)

Additional file 7 Ensembles 10 through 14. This ZIP file includes all of the parameter vectors in Ensembles 10 through 14. (ZIP 11500 kb)

Additional file 8 Ensemble 15. This ZIP file includes all of the parameter vectors in Ensemble 15. (ZIP 10100 kb)

Additional file 9 Ensemble 16. This ZIP file includes all of the parameter vectors in Ensemble 16. (ZIP 9760 kb)

## Abbreviations

AUC: Area under the curve
CV: Coefficient of variation
DE: Differential evolution
LHS: Latin hypercube sampling
ODE: Ordinary differential equation
WT: Wild type

## References

[1] Butcher EC, Berg EL, Kunkel EJ (2004). Systems biology in drug discovery. Nat Biotechnol.

[2] Nelander S, Wang W, Nilsson B, She QB, Pratilas C, Rosen N, Gennemark P, Sander C (2008). Models from experiments: combinatorial drug perturbations of cancer cells. Mol Syst Biol..

[3] Gutenkunst RN, Waterfall JJ, Casey FP, Brown KS, Myers CR, Sethna JP (2007). Universally sloppy parameter sensitivities in systems biology models. PLoS Comput Biol.

[4] Kreutz C, Timmer J (2009). Systems biology: experimental design. FEBS J.

[5] Kuepfer L, Peter M, Sauer U, Stelling J (2007). Ensemble modeling for analysis of cell signaling dynamics. Nat Biotechnol.

[6] Franceschini G, Macchietto S (2008). Model-based design of experiments for parameter precision: State of the art. Chem Eng Sci.

[7] Meyer P, Cokelaer T, Chandran D, Kim KH, Loh PR, Tucker G, Lipson M, Berger B, Kreutz C, Raue A (2014). Network topology and parameter estimation: from experimental design methods to gene regulatory network kinetics using a community based approach. BMC Syst Biol.

[8] Silk D, Kirk PD, Barnes CP, Toni T, Stumpf MP (2014). Model selection in systems biology depends on experimental design. PLoS Comput Biol.

[9] Schaber J, Baltanas R, Bush A, Klipp E, Colman-Lerner A (2012). Modelling reveals novel roles of two parallel signalling pathways and homeostatic feedbacks in yeast. Mol Syst Biol..

[10] Tran LM, Rizk ML, Liao JC (2008). Ensemble modeling of metabolic networks. Biophys J.

[11] Jia G, Stephanopoulos G, Gunawan R (2012). Ensemble kinetic modeling of metabolic networks from dynamic metabolic profiles. Metabolites.

[12] Song SO, Chakrabarti A, Varner JD (2010). Ensembles of signal transduction models using Pareto optimal ensemble techniques (POETs). Biotechnol J.

[13] Noble SL, Buzzard GT, Rundell AE (2011). Feasible parameter space characterization with adaptive sparse grids for nonlinear systems biology models. American Control Conference (ACC), 2011.

[14] Dayarian A, Chaves M, Sontag ED, Sengupta AM (2009). Shape, size, and robustness: feasible regions in the parameter space of biochemical networks. PLoS Comput Biol.

[15] Tiemann C, Vanlier J, Hilbers P, van Riel N (2011). Parameter adaptations during phenotype transitions in progressive diseases. BMC Syst Biol.

[16] Tiemann CA, Vanlier J, Oosterveer MH, Groen AK, Hilbers PA, van Riel NA (2013). Parameter trajectory analysis to identify treatment effects of pharmacological interventions. PLoS Comput Biol.

[17] Rumschinski P, Borchers S, Bosio S, Weismantel R, Findeisen R (2010). Set-base dynamical parameter estimation and model invalidation for biochemical reaction networks. BMC Syst Biol.

[18] Rodriguez-Fernandez M, Rehberg M, Kremling A, Banga JR (2013). Simultaneous model discrimination and parameter estimation in dynamic models of cellular systems. BMC Syst Biol.

[19] Pargett M, Rundell AE, Buzzard GT, Umulis DM (2014). Model-based analysis for qualitative data: an application in drosophila germline stem cell regulation. PLoS Comput Biol.

[20] Donzé A, Fanchon E, Gattepaille LM, Maler O, Tracqui P (2011). Robustness analysis and behavior discrimination in enzymatic reaction networks. PloS ONE.

[21] Pargett M, Umulis DM (2013). Quantitative model analysis with diverse biological data: applications in developmental pattern formation. Methods.

[22] Oguz C, Laomettachit T, Chen KC, Watson LT, Baumann WT, Tyson JJ (2013). Optimization and model reduction in the high dimensional parameter space of a budding yeast cell cycle model. BMC Syst Biol.

[23] Price KV, Storn RM, Lampinen JA (2005). Differential Evolution: A Practical Approach to Global Optimization. Natural Computing Series.

[24] Chong CK, Mohamad MS, Deris S, Shamsir MS, Choon YW, Chai LE (2012). Improved differential evolution algorithm for parameter estimation to improve the production of biochemical pathway. Intl J Interactive Multimedia Artif Intell..

[25] Tashkova K, Korošec P, Šilc J, Todorovski L, Džeroski S (2011). Parameter estimation with bio-inspired meta-heuristic optimization: modeling the dynamics of endocytosis. BMC Syst Biol.

[26] Mahdavi S, Shiri ME, Rahnamayan S (2015). Metaheuristics in large-scale global continues optimization: A survey. Inf Sci.

[27] Sun J, Garibaldi JM, Hodgman C (2012). Parameter estimation using metaheuristics in systems biology: a comprehensive review. Comput Biol Bioinformatics IEEE/ACM Trans.

[28] Banga JR, Versyck KJ, Van Impe JF (2002). Computation of optimal identification experiments for nonlinear dynamic process models: a stochastic global optimization approach. Ind Eng Chem Res.

[29] Rodriguez-Fernandez M, Mendes P, Banga JR (2006). A hybrid approach for efficient and robust parameter estimation in biochemical pathways. Biosystems.

[30] Balsa-Canto E, Alonso AA, Banga JR (2008). Computational procedures for optimal experimental design in biological systems. IET Syst Biol.

[31] Ashyraliyev M, Jaeger J, Blom JG (2008). Parameter estimation and determinability analysis applied to drosophila gap gene circuits. BMC Syst Biol.

[32] Audoly S, Bellu G, D’Angio L, Saccomani MP, Cobelli C (2001). Global identifiability of nonlinear models of biological systems. Biomed Eng IEEE Trans.

[33] Zak DE, Gonye GE, Schwaber JS, Doyle FJ (2003). Importance of input perturbations and stochastic gene expression in the reverse engineering of genetic regulatory networks: insights from an identifiability analysis of an in silico network. Genome Res.

[34] Morgan DO (2007). The Cell Cycle: Principles of Control.

[35] Mitchison JM (1971). The Biology of the Cell Cycle.

[36] Chen KC, Csikasz-Nagy A, Gyorffy B, Val J, Novak B, Tyson JJ (2000). Kinetic analysis of a molecular model of the budding yeast cell cycle. Mol Biol Cell.

[37] Chen KC, Calzone L, Csikasz-Nagy A, Cross FR, Novak B, Tyson JJ (2004). Integrative analysis of cell cycle control in budding yeast,. Mol Biol Cell.

[38] Singhania R, Sramkoski RM, Jacobberger JW, Tyson JJ (2011). A hybrid model of mammalian cell cycle regulation. PLoS Comput Biol.

[39] Kraikivski P, Chen KC, Laomettachit T, Murali T, Tyson JJ (2015). From start to finish: computational analysis of cell cycle control in budding yeast. npj Syst Biol Appl.

[40] Laomettachit T. Mathematical modeling approaches for dynamical analysis of protein regulatory networks with applications to the budding yeast cell cycle and the circadian rhythm in cyanobacteria. PhD thesis, Virginia Institute of Technology. 2011. http://scholar.lib.vt.edu/theses/available/etd-11072011-021528/.

[41] Laomettachit T, Chen KC, Baumann WT, Tyson JJ (2016). A model of yeast cell-cycle regulation based on a standard component modeling strategy for protein regulatory networks. PloS ONE.

[42] Donahue MM, Buzzard GT, Rundell AE (2009). Robust parameter identification with adaptive sparse grid-based optimization for nonlinear systems biology models. American Control Conference, 2009. ACC’09.

[43] Taylor SC, Berkelman T, Yadav G, Hammond M (2013). A defined methodology for reliable quantification of western blot data. Mol Biotechnol..

[44] Oda Y, Huang K, Cross FR, Cowburn D, Chait BT (1999). Accurate quantitation of protein expression and site-specific phosphorylation. Proc Natl Acad Sci.

[45] Bucher J, Riedmaier S, Schnabel A, Marcus K, Vacun G, Weiss T, Thasler W, Nüssler A, Zanger U, Reuss M (2011). A systems biology approach to dynamic modeling and inter-subject variability of statin pharmacokinetics in human hepatocytes. BMC Syst Biol.

[46] Shankaran H, Zhang Y, Tan Y, Resat H (2013). Model-based analysis of HER activation in cells co-expressing EGFR, HER2 and HER3. PLoS Comput Biol.

[47] Dong W, Tang X, Yu Y, Nilsen R, Kim R, Griffith J, Arnold J, Schüttler HB (2008). Systems biology of the clock in neurospora crassa. PloS ONE.

[48] Donahue M, Buzzard G, Rundell A (2010). Experiment design through dynamical characterisation of non-linear systems biology models utilising sparse grids. IET Syst Biol.

[49] Lucia A, DiMaggio PA, Depa P (2004). Funneling algorithms for multiscale optimization on rugged terrains. Ind Eng Chem Res.

[50] Moriya H, Shimizu-Yoshida Y, Kitano H. In Vivo Robustness Analysis of Cell Division Cycle Genes in *Saccharomyces cerevisiae*. PLOS Genet. 2010; 6(4). doi:10.1371/journal.pgen.002011. 10.1371/journal.pgen.0020111 PMC1500812 16839182

[51] Shirayama M, Tóth A, Gálová M, Nasmyth K (1999). Apccdc20 promotes exit from mitosis by destroying the anaphase inhibitor pds1 and cyclin clb5. Nature.

[52] Dwight SS, Harris MA, Dolinski K, Ball CA, Binkley G, Christie KR, Fisk DG, Issel-Tarver L, Schroeder M, Sherlock G (2002). Saccharomyces genome database (sgd) provides secondary gene annotation using the gene ontology (go). Nucleic Acids Res.

[53] Turner JJ, Ewald JC, Skotheim JM (2012). Cell size control in yeast. Curr Biol.

[54] Johnston G, Pringle J, Hartwell LH (1977). Coordination of growth with cell division in the yeast saccharomyces cerevisiae. Experimental Cell Res.

[55] Di Talia S, Wang H, Skotheim JM, Rosebrock AP, Futcher B, Cross FR (2009). Daughter-specific transcription factors regulate cell size control in budding yeast. PLoS Biol.

[56] Di Talia S, Skotheim JM, Bean JM, Siggia ED, Cross FR (2007). The effects of molecular noise and size control on variability in the budding yeast cell cycle. Nature.

[57] MATLAB (2013). Version 8.1 (R2013a).

[58] Hanley JA, McNeil BJ (1982). The meaning and use of the area under a receiver operating characteristic (ROC) curve. Radiology.

[59] Efron B (1979). Bootstrap methods: another look at the jackknife. Annals Stat..

[60] Dasgupta A, Sun YV, König IR, Bailey-Wilson JE, Malley JD (2011). Brief review of regression-based and machine learning methods in genetic epidemiology: the genetic analysis workshop 17 experience. Genet Epidemiol.

[61] Vogel C, Marcotte EM (2012). Insights into the regulation of protein abundance from proteomic and transcriptomic analyses. Nat Rev Genet.

[62] Schwanhäusser B, Busse D, Li N, Dittmar G, Schuchhardt J, Wolf J, Chen W, Selbach M (2011). Global quantification of mammalian gene expression control. Nature.

[63] Vogel C, de Sousa Abreu R, Ko D, Le SY, Shapiro BA, Burns SC, Sandhu D, Boutz DR, Marcotte EM, Penalva LO (2010). Sequence signatures and mrna concentration can explain two-thirds of protein abundance variation in a human cell line. Mol Syst Biol.

[64] Plotkin JB (2010). Transcriptional regulation is only half the story. Mol Syst Biol.

[65] Maier T, Schmidt A, Güell M, Kühner S, Gavin AC, Aebersold R, Serrano L (2011). Quantification of mrna and protein and integration with protein turnover in a bacterium. Mol Syst Biol.

