# Supplementary material for: Predicting network modules of cell cycle regulators using relative protein abundance statistics
Source: BMC Syst Biol. 2017 Feb 28;11:30. doi: 10.1186/s12918-017-0409-1 (PMC5329933; doi:10.1186/s12918-017-0409-1)
Supplement: Additional file 1 — Supplementary Tables. This pdf file includes 13 tables referred to in the main text. (PDF 175 kb) [file 12918_2017_409_MOESM1_ESM.pdf]

Table S1: **Model parameters**

Parameter	Description	Parameter	Description
γ	Time scale for protein activation	kd_{swi5}	Degradation rate of Swi5
γ_{ki}	CKI inactivation time scale	$ka_{swi5,14}$	Swi5 activation by Cdc14
γ_{cp}	APC activation time scale	$ki_{swi5,b2}$	Swi5 inactivation by Clb2
γ_{tem}	Tem1 activation time scale	$ka_{m1,b2}$	Mcm1 activation by Clb2
σ	Sigmoidicity of protein activation	ki_{m1}	Basal Mcm1 inactivation
σ_{net}	Sigmoidicity of Net1 activation	ks_{20}	Basal Cdc20 synthesis
ks_{n3}	Cln3 synthesis rate	$ks_{20,m1}$	Mcm1-dependent Cdc20 synthesis
J_{n3}	Michaelis-Menten constant	kd_{20}	Cdc20 degradation
D_{n3}	Dosage of CLN3 gene	ka_{20}	Basal Cdc20 activation
kd_{n3}	Cln3 degradation rate	$kd_{b5,20,i}$	Clb5 degradation by Cdc20
ks_{k2}	Bck2 synthesis rate	$kd_{clb2,20,i}$	Clb2 degradation by Cdc20
kd_{k2}	Bck2 degradation rate	$ki_{20,ori}$	Cdc20 inactivation by spindle checkpoint
kdp_{i5}	Basal Whi5 dephosphorylation	$ka_{cp,b2}$	APC phosphorylation by Clb2
kdp_{i514}	Whi5 dephosphorylation by Cdc14	ki_{cp}	APC inactivation
kp_{i5}	Basal Whi5 phosphorylation	ka_{h1}	Basal Cdh1 activation
kp_{i5n3}	Whi5 phosphorylation by Cln3	$ka_{h1,14}$	Cdh1 activation by Cdc14
kp_{i5k2}	Whi5 phosphorylation by Bck2	ki_{h1}	Basal inactivation of Cdh1
kp_{i5n2}	Whi5 phosphorylation by Cln2	$ki_{h1,e}$	Cdh1 inactivation rate
kp_{i5b5}	Whi5 phosphorylation by Clb5	$e_{h1,n3}$	Cdh1 inactivation by Cln3
kdp_{bf}	Basal SBF dephosphorylation	$e_{h1,n2}$	Cdh1 inactivation by Cln2
kp_{bfb2}	SBF phosphorylation by Clb2	$e_{h1,b5}$	Cdh1 inactivation by Clb5
ks_{n2}	Basal Cln2 synthesis rate	$e_{h1,b2}$	Cdh1 inactivation by Clb2
ks_{n2bf}	SBF-dependent Cln2 synthesis	kdp_{net}	Basal Net1 dephosphorylation
kd_{n2}	Cln2 degradation	$kdp_{net,14}$	Net1 dephosphorylation by Cdc14
ks_{ki}	Basal CKI synthesis rate	$kdp_{net,px}$	Net1 dephosphorylation by PPX
$ks_{ki,swi5}$	Swi5-dependent CKI synthesis	kp_{net}	Basal Net1 phosphorylation
kd_{ki}	Degradation rate of CKI	$kp_{net,b2}$	Net1 phosphorylation by Clb2
kd_{kip}	Degradation rate of CKIP	$kp_{net,en}$	Net1 phosphorylation by MEN
$kp_{ki,e}$	CKI phosphorylation rate	$kp_{net,15}$	Net1 phosphorylation by Cdc15
$e_{ki,n3}$	CKI phosphorylation by Cln3	ka_{px}	Basal PPX activation
$e_{ki,k2}$	CKI phosphorylation by Bck2	ki_{px}	Basal PPX inactivation
$e_{ki,n2}$	CKI phosphorylation by Cln2	$ki_{px,p1}$	PPX inactivation by Esp1
$e_{ki,b5}$	CKI phosphorylation by Clb5	ks_{pds}	Basal Pds1 synthesis
$e_{ki,b2}$	CKI phosphorylation by Clb2	kd_{pds}	Basal Pds1 degradation
kdp_{ki}	Basal dephosphorylation of CKI	$kd_{pds,20}$	Pds1 degradation by Cdc20A
$kdp_{ki,14}$	CKI dephosphorylation by Cdc14	$kd_{pds,20,i}$	Pds1 degradation by Cdc20
ks_{b5}	Basal Clb5 synthesis	ka_{15}	Basal Cdc15 activation
$ks_{b5,bf}$	SBF-dependent Clb5 synthesis	$ka_{15,14}$	Cdc15 activation by Cdc14
kd_{b5}	Basal Clb5 degradation	ki_{15}	Basal Cdc15 inactivation
$kd_{b5,20}$	Clb5 degradation by Cdc20A	$ki_{15,b2}$	Cdc15 inactivation by Clb2
ks_{b2}	Basal Clb2 synthesis	ka_{tem}	Basal Tem1 activation
$ks_{b2,m1}$	Mcm1-dependent Clb2 synthesis	$ka_{tem,lo}$	Tem1 activation by Polo
kd_{b2}	Basal Clb2 degradation	$ka_{tem,p1}$	Tem1 activation by Esp1
$kd_{b2,20}$	Clb2 degradation by Cdc20A	ki_{tem}	Basal inactivation of Tem1
$kd_{b2,h1}$	Clb2 degradation by Cdh1A	$ki_{tem,px}$	Tem1 inactivation by PPX

Continued on next page

Table S1 – *Continued from previous page*

Parameter	Description	Parameter	Description
$ks_{bud,e}$	Time scale for BUD synthesis	ks_{lo}	Basal Polo synthesis
$e_{bud,n3}$	Cln3 activation of BUD	$ks_{lo,m1}$	Mcm1-dependent synthesis of Polo
$e_{bud,n2}$	Cln2 activation of BUD	kd_{lo}	Basal Polo degradation
$e_{bud,b5}$	Clb5 activation of BUD	$kd_{lo,h1}$	Polo degradation by Cdh1
$e_{bud,b2}$	Clb2 activation of BUD	ka_{lo}	Basal Polo activation
kd_{bud}	BUD degradation	$ka_{lo,b2}$	Polo activation by Clb2
ks_{spn}	SPN synthesis	ki_{lo}	Basal Polo inactivation
kd_{spn}	SPN degradation	kas_{net}	Efficiency of Cdc14-Net1 complex (RENT) formation
J_{spn}	SPN synthesis threshold	f	Fraction of mass retained by daughter at division
$ks_{ori,e}$	Time scale for ORI synthesis	MDT	Mass doubling time
$e_{ori,b5}$	Clb5 activation of ORI	$Whi5_T$	Total Whi5
$e_{ori,b2}$	Clb2 activation of ORI	SBF_T	Total SBF
kd_{ori}	Degradation of ORI	$Mcm1_T$	Total Mcm1
ks_{swi5}	Basal Swi5 synthesis	APC_T	Total APC
$ks_{swi5,m1}$	Mcm1-dependent Swi5 synthesis	$Cdh1_T$	Total Cdh1
		$Net1_T$	Total Net1
		$Cdc14_T$	Total Cdc14
		PPX_T	Total PPX
		$Esp1_T$	Total Esp1
		$Cdc15_T$	Total Cdc15
		$Tem1_T$	Total Tem1

Table S2: **Model variables**

Variable	Description
V	Cell size
[Cln3]	Concentration of G1 cyclin Cln3
[Bck2]	Concentration of Bck2, an activator for START transition
[WHI5dep]	Dephosphorylated (active) Whi5
[SBFdep]	Dephosphorylated SBF
[Cln2]	Total concentration of G1 cyclins Cln1,2
$[\mathrm{CKI}_T]$	Total concentration of Sic1+Cdc6, stoichiometric inhibitors of Clb5,6 and Clb1,2
$[\mathrm{CKI}_P]$	Phosphorylated form of Sic1+Cdc6, stoichiometric inhibitors of Clb5,6 and Clb1,2
$[\mathrm{Clb5}_T]$	Total concentration of free B cyclins Clb5,6
$[\mathrm{Clb2}_T]$	Total concentration of free B cyclins Clb1,2
[BUD]	Variable for bud emergence progression
[ORI]	Variable for DNA synthesis progression
[SPN]	Variable for spindle assembly progression
$[\mathrm{Swi5}_T]$	Total concentration of SWI5, transcription factor for CKI synthesis
$[\mathrm{CDC20}_T]$	Total concentration of Cdc20, a protein involved in Clb5,6, Clb1,2 and Pds1 degradation
[APCP]	Phosphorylated (active) APC, activates Cdc20 by complex formation
$[\mathrm{Cdh1}_A]$	Active Cdh1 degrading Clb1,2 and Polo kinase
[Net1dep]	Dephosphorylated (active) Net1, a stoichiometric inhibitor of Cdc14
[PPX]	Phosphatase that dephosphorylates Net1
$[\mathrm{Pds1}_T]$	Securin, Esp1's stoichiometric inhibitor
[Cdc15]	Kinase, when complexed with Tem1, phosphorylates Net1
[Tem1]	Protein, when complexed with Cdc15, phosphorylates Net1
$[\mathrm{Polo}_T]$	Total concentration of Polo kinase that activates Tem1
$[\mathrm{Polo}_A]$	Concentration of phosphorylated (active) Polo kinase that activates Tem1
[Cdc20A-APC]	Complex formed from active Cdc20 and APC
[Cdc20A-APCP]	Complex formed from active Cdc20 and APC-P

Initial values of these variables in the wild type (glucose) simulations are also optimized with the model parameters. Each variable corresponds to a single ODE. Initial conditions for simulating the genetic strains other than wild type (glucose) come from the cell state right after the last division in 2000 min wild type simulations.

Table S3: **Kinetic parameter ranges in Ensemble 1**

Parameter	Range in Ensemble 1	Parameter	Range in Ensemble 1
γ	0.4484–0.5024	kd_{swi5}	0.0965–0.1039
γ_{ki}	9.8180–10.7689	$ka_{swi5,14}$	1.3797–1.4480
γ_{cp}	0.4971–0.5600	$ki_{swi5,b2}$	0.0452–0.0465
γ_{tem}	0.6562–0.6635	$ka_{m1,b2}$	11.1548 11.2010
σ	9.9283–10.2929	ki_{m1}	1.1396–1.3243
σ_{net}	11.3173–11.4912	ks_{20}	0.0084 0.0087
ks_{n3}	0.7918–0.8308	$ks_{20,m1}$	0.6590–0.6755
J_{n3}	4.9037–5.5114	kd_{20}	0.2642–0.2704
D_{n3}	0.6581–0.7951	ka_{20}	0.0802–0.0833
kd_{n3}	3.0653–3.0831	$kd_{b5,20,i}$	0.0160–0.0163
ks_{k2}	0.1636–0.1655	$kd_{clb2,20,i}$	0.0321–0.0323
kd_{k2}	2.2752–2.3940	$ki_{20,ori}$	7.9895–8.1284
kdp_{i5}	0.6684–0.7028	$ka_{cp,b2}$	1.2888–1.3928
kdp_{i514}	0.1423–0.1433	ki_{cp}	1.0685–1.1014
kp_{i5}	0.0934–0.1173	ka_{h1}	0.9869–1.1131
kp_{i5n3}	8.0040–8.0810	$ka_{h1,14}$	6.7577–6.8959
kp_{i5k2}	5.8573–6.2917	ki_{h1}	0.1144–0.1331
kp_{i5n2}	9.0349–10.9157	$ki_{h1,e}$	1.3630–1.3726
kp_{i5b5}	0.0597–0.0707	$e_{h1,n3}$	0.3428–0.4476
kdp_{bf}	0.7961–0.8289	$e_{h1,n2}$	0.5882–0.6473
kp_{bfb2}	9.1500–9.5944	$e_{h1,b5}$	6.4725–7.3508
ks_{n2}	0.0000–0.0000	$e_{h1,b2}$	6.6411–6.8824
ks_{n2bf}	0.5035–0.5413	kdp_{net}	0.7716–0.8149
kd_{n2}	0.3359–0.3439	$kdp_{net,14}$	0.0671–0.0672
ks_{ki}	0.0075–0.0088	$kdp_{net,px}$	9.3856–10.6345
$ks_{ki,swi5}$	0.0894–0.0979	kp_{net}	0.1320–0.1364
kd_{ki}	0.0153–0.0155	$kp_{net,b2}$	0.9586–1.0246
kd_{kip}	1.6227–1.6775	$kp_{net,en}$	8.1055–8.4092
$kp_{ki,e}$	1.0245–1.0599	$kp_{net,15}$	0.0601–0.0602
$e_{ki,n3}$	2.1481–2.1760	ka_{px}	0.6003–0.7264
$e_{ki,k2}$	0.6634–0.7247	ki_{px}	0.1161–0.1339
$e_{ki,n2}$	1.0077–1.0368	$ki_{px,p1}$	3.2938–3.4142
$e_{ki,b5}$	2.3180–2.3752	ks_{pds}	0.0296–0.0309
$e_{ki,b2}$	1.9663–2.6812	kd_{pds}	0.0347–0.0357
kdp_{ki}	0.7946–0.8847	$kd_{pds,20}$	2.2570–2.5039
$kdp_{ki,14}$	2.6829–3.7045	$kd_{pds,20,i}$	0.3551–0.3798
ks_{b5}	0.0021–0.0024	ka_{15}	0.0759–0.0870
$ks_{b5,bf}$	0.0108–0.0110	$ka_{15,14}$	4.3358–4.5972
kd_{b5}	0.0050–0.0061	ki_{15}	0.6738–0.7610
$kd_{b5,20}$	0.1477–0.1543	$ki_{15,b2}$	0.5729–0.8190
ks_{b2}	0.0028–0.0030	ka_{tem}	0.1143–0.1180
$ks_{b2,m1}$	0.1006–0.1053	$ka_{tem,lo}$	2.4195–2.4905
kd_{b2}	0.0023–0.0023	$ka_{tem,p1}$	0.1131–0.1243
$kd_{b2,20}$	0.1210–0.1224	ki_{tem}	0.8592–0.8989

Continued on next page

Table S3 – *Continued from previous page*

Parameter	Range in Ensemble 1	Parameter	Range in Ensemble 1
$kd_{b2,h1}$	0.5176–0.5313	$ki_{tem,px}$	1.5929–2.0781
$ks_{bud,e}$	0.2005–0.2112	ks_{lo}	0.0102–0.0121
$e_{bud,n3}$	0.0481–0.0507	$ks_{lo,m1}$	0.0999–0.1108
$e_{bud,n2}$	0.3111–0.3449	kd_{lo}	0.0115–0.0139
$e_{bud,b5}$	0.8390–0.9075	$kd_{lo,h1}$	0.0373–0.0377
$e_{bud,b2}$	0.0466–0.0936	ka_{lo}	0.0637–0.0649
kd_{bud}	0.0695–0.0909	$ka_{lo,b2}$	2.9338–3.3335
ks_{spn}	0.1338–0.1424	ki_{lo}	1.4757–1.7254
kd_{spn}	0.0573–0.0601	kas_{net}	1.2493–1.2684
J_{spn}	0.0982–0.1083	f	0.4000–0.4000
$ks_{ori,e}$	2.3406–2.4693	MDT	100.0000–100.0000
$e_{ori,b5}$	0.7648–0.8134	$Whi5_T$	2.2348–2.5219
$e_{ori,b2}$	0.5872–0.6044	SBF_T	0.9452–1.1215
kd_{ori}	0.0495–0.0519	$Mcm1_T$	0.6585–0.7097
ks_{swi5}	0.0042–0.0045	APC_T	34.7611–35.1986
$ks_{swi5,m1}$	0.1017–0.1031	$Cdh1_T$	1.2272–1.2465
		$Net1_T$	2.9743–3.0579
		$Cdc14_T$	2.0347– 2.1213
		PPX_T	1.1038– 1.1345
		$Esp1_T$	0.3832– 0.3858
		$\mathrm{Cdc15}_T$	0.7405–0.8525
		$Tem1_T$	1.3604– 1.5572

These ranges are used in LH sampling to generate Ensemble 2 (details described in Section 2 of Supplementary Text).

Table S4: **Initial condition ranges in Ensemble 1**

Variable	Range in Ensemble 1
V_0	0.6772–1.6213
$[Cln3]_0$	0.2495–0.2551
$[Bck2]_0$	0.0829–0.0873
$[WHI5dep]_0$	3.0876–3.5306
$[SBFdep]_0$	0.5325–1.1732
$[Cln2]_0$	0.0886–0.1248
$[CKI_T]_0$	1.1979–1.4018
$[CKI_P]_0$	0.1080–0.1131
$[Clb5_T]_0$	0.2858–0.2899
$[Clb2_T]_0$	0.1290–0.1315
$[BUD]_0$	0.0185–0.0236
$[ORI]_0$	0.0768–0.1094
$[SPN]_0$	0.1042–0.1078
$[Swi5_T]_0$	0.1698–0.1940
$[CDC20_T]_0$	0.0208–0.0216
$[APCP]_0$	0.0800–0.0997
$[Cdh1_A]_0$	1.0712–1.1167
$[Net1dep]_0$	2.4645–2.4826
$[PPX]_0$	0.9957–1.0066
$[Pds1_T]_0$	0.2571–0.2790
$[Cdc15]_0$	0.1004–0.1095
$[Tem1]_0$	0.0900–0.1036
$[Polo_T]_0$	0.8896–0.9982
$[Polo_A]_0$	0.0783–0.0907
$[Cdc20A\text{-}APC]_0$	0.1093–0.1150
$[Cdc20A\text{-}APCP]_0$	0.0919–0.0929

These ranges are used in LH sampling to generate Ensemble 2. Initial conditions for simulating the genetic strains other than wild type (glucose) come from the cell state right after the last division in 2000 min wild type simulations.

Table S5: **Novel mutants (Prediction Set)**

Novel mutant #	Mutation 1	Mutation 2	Mutation 3	Novel mutant #	Mutation 1	Mutation 2	Mutation 3
1	$kp_{i5k2} = 0$	-	-	66	$kp_{i5k2} = 0$	$kp_{i5n3} = 0$	$kdp_{i514} = 0$
2	$e_{ki,n2} = 0$	-	-	67	$kp_{i5k2} = 0$	$kp_{i5n3} = 0$	$kdp_{net,px} = 0$
3	$e_{ki,b2} = 0$	-	-	68	$kp_{i5k2} = 0$	$kp_{bfb2} = 0$	$kp_{i5n2} = 0$
4	$kdp_{ki,14} = 0$	-	-	69	$kp_{i5k2} = 0$	$kp_{bfb2} = 0$	$kdp_{i514} = 0$
5	$kp_{i5n3} = 0$	-	-	70	$kp_{i5k2} = 0$	$kp_{bfb2} = 0$	$kdp_{net,px} = 0$
6	$kp_{bfb2} = 0$	-	-	71	$kp_{i5k2} = 0$	$kp_{i5n2} = 0$	$kdp_{i514} = 0$
7	$kp_{i5n2} = 0$	-	-	72	$kp_{i5k2} = 0$	$kp_{i5n2} = 0$	$kdp_{net,px} = 0$
8	$kdp_{i514} = 0$	-	-	73	$kp_{i5k2} = 0$	$kdp_{i514} = 0$	$kdp_{net,px} = 0$
9	$kdp_{net,px} = 0$	-	-	74	$e_{ki,n2} = 0$	$e_{ki,b2} = 0$	$kdp_{ki,14} = 0$
10	$kp_{i5k2} = 0$	$e_{ki,n2} = 0$	-	75	$e_{ki,n2} = 0$	$e_{ki,b2} = 0$	$kp_{i5n3} = 0$
11	$kp_{i5k2} = 0$	$e_{ki,b2} = 0$	-	76	$e_{ki,n2} = 0$	$e_{ki,b2} = 0$	$kp_{bfb2} = 0$
12	$kp_{i5k2} = 0$	$kdp_{ki,14} = 0$	-	77	$e_{ki,n2} = 0$	$e_{ki,b2} = 0$	$kp_{i5n2} = 0$
13	$kp_{i5k2} = 0$	$kp_{i5n3} = 0$	-	78	$e_{ki,n2} = 0$	$e_{ki,b2} = 0$	$kdp_{i514} = 0$
14	$kp_{i5k2} = 0$	$kp_{bfb2} = 0$	-	79	$e_{ki,n2} = 0$	$e_{ki,b2} = 0$	$kdp_{net,px} = 0$
15	$kp_{i5k2} = 0$	$kp_{i5n2} = 0$	-	80	$e_{ki,n2} = 0$	$kdp_{ki,14} = 0$	$kp_{i5n3} = 0$
16	$kp_{i5k2} = 0$	$kdp_{i514} = 0$	-	81	$e_{ki,n2} = 0$	$kdp_{ki,14} = 0$	$kp_{bfb2} = 0$
17	$kp_{i5k2} = 0$	$kdp_{net,px} = 0$	-	82	$e_{ki,n2} = 0$	$kdp_{ki,14} = 0$	$kp_{i5n2} = 0$
18	$e_{ki,n2} = 0$	$e_{ki,b2} = 0$	-	83	$e_{ki,n2} = 0$	$kdp_{ki,14} = 0$	$kdp_{i514} = 0$
19	$e_{ki,n2} = 0$	$kdp_{ki,14} = 0$	-	84	$e_{ki,n2} = 0$	$kdp_{ki,14} = 0$	$kdp_{net,px} = 0$
20	$e_{ki,n2} = 0$	$kp_{i5n3} = 0$	-	85	$e_{ki,n2} = 0$	$kp_{i5n3} = 0$	$kp_{bfb2} = 0$
21	$e_{ki,n2} = 0$	$kp_{bfb2} = 0$	-	86	$e_{ki,n2} = 0$	$kp_{i5n3} = 0$	$kp_{i5n2} = 0$
22	$e_{ki,n2} = 0$	$kp_{i5n2} = 0$	-	87	$e_{ki,n2} = 0$	$kp_{i5n3} = 0$	$kdp_{i514} = 0$
23	$e_{ki,n2} = 0$	$kdp_{i514} = 0$	-	88	$e_{ki,n2} = 0$	$kp_{i5n3} = 0$	$kdp_{net,px} = 0$
24	$e_{ki,n2} = 0$	$kdp_{net,px} = 0$	-	89	$e_{ki,n2} = 0$	$kp_{bfb2} = 0$	$kp_{i5n2} = 0$
25	$e_{ki,b2} = 0$	$kdp_{ki,14} = 0$	-	90	$e_{ki,n2} = 0$	$kp_{bfb2} = 0$	$kdp_{i514} = 0$
26	$e_{ki,b2} = 0$	$kp_{i5n3} = 0$	-	91	$e_{ki,n2} = 0$	$kp_{bfb2} = 0$	$kdp_{net,px} = 0$
27	$e_{ki,b2} = 0$	$kp_{bfb2} = 0$	-	92	$e_{ki,n2} = 0$	$kp_{i5n2} = 0$	$kdp_{i514} = 0$
28	$e_{ki,b2} = 0$	$kp_{i5n2} = 0$	-	93	$e_{ki,n2} = 0$	$kp_{i5n2} = 0$	$kdp_{net,px} = 0$
29	$e_{ki,b2} = 0$	$kdp_{i514} = 0$	-	94	$e_{ki,n2} = 0$	$kdp_{i514} = 0$	$kdp_{net,px} = 0$
30	$e_{ki,b2} = 0$	$kdp_{net,px} = 0$	-	95	$e_{ki,b2} = 0$	$kdp_{ki,14} = 0$	$kp_{i5n3} = 0$
31	$kdp_{ki,14} = 0$	$kp_{i5n3} = 0$	-	96	$e_{ki,b2} = 0$	$kdp_{ki,14} = 0$	$kp_{bfb2} = 0$
32	$kdp_{ki,14} = 0$	$kp_{bfb2} = 0$	-	97	$e_{ki,b2} = 0$	$kdp_{ki,14} = 0$	$kp_{i5n2} = 0$
33	$kdp_{ki,14} = 0$	$kp_{i5n2} = 0$	-	98	$e_{ki,b2} = 0$	$kdp_{ki,14} = 0$	$kdp_{i514} = 0$
34	$kdp_{ki,14} = 0$	$kdp_{i514} = 0$	-	99	$e_{ki,b2} = 0$	$kdp_{ki,14} = 0$	$kdp_{net,px} = 0$
35	$kdp_{ki,14} = 0$	$kdp_{net,px} = 0$	-	100	$e_{ki,b2} = 0$	$kp_{i5n3} = 0$	$kp_{bfb2} = 0$
36	$kp_{i5n3} = 0$	$kp_{bfb2} = 0$	-	101	$e_{ki,b2} = 0$	$kp_{i5n3} = 0$	$kp_{i5n2} = 0$
37	$kp_{i5n3} = 0$	$kp_{i5n2} = 0$	-	102	$e_{ki,b2} = 0$	$kp_{i5n3} = 0$	$kdp_{i514} = 0$
38	$kp_{i5n3} = 0$	$kdp_{i514} = 0$	-	103	$e_{ki,b2} = 0$	$kp_{i5n3} = 0$	$kdp_{net,px} = 0$
39	$kp_{i5n3} = 0$	$kdp_{net,px} = 0$	-	104	$e_{ki,b2} = 0$	$kp_{bfb2} = 0$	$kp_{i5n2} = 0$
40	$kp_{bfb2} = 0$	$kp_{i5n2} = 0$	-	105	$e_{ki,b2} = 0$	$kp_{bfb2} = 0$	$kdp_{i514} = 0$
41	$kp_{bfb2} = 0$	$kdp_{i514} = 0$	-	106	$e_{ki,b2} = 0$	$kp_{bfb2} = 0$	$kdp_{net,px} = 0$
42	$kp_{bfb2} = 0$	$kdp_{net,px} = 0$	-	107	$e_{ki,b2} = 0$	$kp_{i5n2} = 0$	$kdp_{i514} = 0$
43	$kp_{i5n2} = 0$	$kdp_{i514} = 0$	-	108	$e_{ki,b2} = 0$	$kp_{i5n2} = 0$	$kdp_{net,px} = 0$
44	$kp_{i5n2} = 0$	$kdp_{net,px} = 0$	-	109	$e_{ki,b2} = 0$	$kdp_{i514} = 0$	$kdp_{net,px} = 0$
45	$kdp_{i514} = 0$	$kdp_{net,px} = 0$	-	110	$kdp_{ki,14} = 0$	$kp_{i5n3} = 0$	$kp_{bfb2} = 0$

Continued on next page

Table S5 – *Continued from previous page*

Novel mutant #	Mutation 1	Mutation 2	Mutation 3	Novel mutant #	Mutation 1	Mutation 2	Mutation 3
46	$kp_{i5k2} = 0$	$e_{ki,n2} = 0$	$e_{ki,b2} = 0$	111	$kdp_{ki,14} = 0$	$kp_{i5n3} = 0$	$kp_{i5n2} = 0$
47	$kp_{i5k2} = 0$	$e_{ki,n2} = 0$	$kdp_{ki,14} = 0$	112	$kdp_{ki,14} = 0$	$kp_{i5n3} = 0$	$kdp_{i514} = 0$
48	$kp_{i5k2} = 0$	$e_{ki,n2} = 0$	$kp_{i5n3} = 0$	113	$kdp_{ki,14} = 0$	$kp_{i5n3} = 0$	$kdp_{net,px} = 0$
49	$kp_{i5k2} = 0$	$e_{ki,n2} = 0$	$kp_{bfb2} = 0$	114	$kdp_{ki,14} = 0$	$kp_{bfb2} = 0$	$kp_{i5n2} = 0$
50	$kp_{i5k2} = 0$	$e_{ki,n2} = 0$	$kp_{i5n2} = 0$	115	$kdp_{ki,14} = 0$	$kp_{bfb2} = 0$	$kdp_{i514} = 0$
51	$kp_{i5k2} = 0$	$e_{ki,n2} = 0$	$kdp_{i514} = 0$	116	$kdp_{ki,14} = 0$	$kp_{bfb2} = 0$	$kdp_{net,px} = 0$
52	$kp_{i5k2} = 0$	$e_{ki,n2} = 0$	$kdp_{net,px} = 0$	117	$kdp_{ki,14} = 0$	$kp_{i5n2} = 0$	$kdp_{i514} = 0$
53	$kp_{i5k2} = 0$	$e_{ki,b2} = 0$	$kdp_{ki,14} = 0$	118	$kdp_{ki,14} = 0$	$kp_{i5n2} = 0$	$kdp_{net,px} = 0$
54	$kp_{i5k2} = 0$	$e_{ki,b2} = 0$	$kp_{i5n3} = 0$	119	$kdp_{ki,14} = 0$	$kdp_{i514} = 0$	$kdp_{net,px} = 0$
55	$kp_{i5k2} = 0$	$e_{ki,b2} = 0$	$kp_{bfb2} = 0$	120	$kp_{i5n3} = 0$	$kp_{bfb2} = 0$	$kp_{i5n2} = 0$
56	$kp_{i5k2} = 0$	$e_{ki,b2} = 0$	$kp_{i5n2} = 0$	121	$kp_{i5n3} = 0$	$kp_{bfb2} = 0$	$kdp_{i514} = 0$
57	$kp_{i5k2} = 0$	$e_{ki,b2} = 0$	$kdp_{i514} = 0$	122	$kp_{i5n3} = 0$	$kp_{bfb2} = 0$	$kdp_{net,px} = 0$
58	$kp_{i5k2} = 0$	$e_{ki,b2} = 0$	$kdp_{net,px} = 0$	123	$kp_{i5n3} = 0$	$kp_{i5n2} = 0$	$kdp_{i514} = 0$
59	$kp_{i5k2} = 0$	$kdp_{ki,14} = 0$	$kp_{i5n3} = 0$	124	$kp_{i5n3} = 0$	$kp_{i5n2} = 0$	$kdp_{net,px} = 0$
60	$kp_{i5k2} = 0$	$kdp_{ki,14} = 0$	$kp_{bfb2} = 0$	125	$kp_{i5n3} = 0$	$kdp_{i514} = 0$	$kdp_{net,px} = 0$
61	$kp_{i5k2} = 0$	$kdp_{ki,14} = 0$	$kp_{i5n2} = 0$	126	$kp_{bfb2} = 0$	$kp_{i5n2} = 0$	$kdp_{i514} = 0$
62	$kp_{i5k2} = 0$	$kdp_{ki,14} = 0$	$kdp_{i514} = 0$	127	$kp_{bfb2} = 0$	$kp_{i5n2} = 0$	$kdp_{net,px} = 0$
63	$kp_{i5k2} = 0$	$kdp_{ki,14} = 0$	$kdp_{net,px} = 0$	128	$kp_{bfb2} = 0$	$kdp_{i514} = 0$	$kdp_{net,px} = 0$
64	$kp_{i5k2} = 0$	$kp_{i5n3} = 0$	$kp_{bfb2} = 0$	129	$kp_{i5n2} = 0$	$kdp_{i514} = 0$	$kdp_{net,px} = 0$
65	$kp_{i5k2} = 0$	$kp_{i5n3} = 0$	$kp_{i5n2} = 0$				

Table S6: **List of 119 phenotypes (Training Set)**

Phenotype #	Phenotype name	Viable/Inviable	Changes in WT parameters
1	WT in glucose	Viable	NONE
2	WT in galactose	Viable	MDT=150; f=0.48;
3	*cln3*Δ	Viable	D_{n3}=0; [CLN3]=0;
4	*bck2*Δ	Viable	ks_{k2}=0; [BCK2]=0;
5	*cln3*Δ *bck2*Δ	Inviable	D_{n3}=0; [CLN3]=0; ks_{k2}=0;[BCK2]=0;
6	*cln3*Δ *bck2*Δ multicopy *CLN2*	Inviable	D_{n3}=0; [CLN3]=0; ks_{k2}=0; [BCK2]=0; ks_{n2bf}=ks_{n2bf}*2;
7	*cln3*Δ *bck2*Δ *sic*Δ	Inviable	D_{n3}=0; [CLN3]=0; ks_{k2}=0; [BCK2]=0; ks_{ki}=ks_{ki}*0.125; $ks_{ki,swi5}$=$ks_{ki,swi5}$*0.125; [CKIT]=0.2;
8	*cln3*Δ *bck2*Δ *whi5*Δ	Viable	D_{n3}=0; [CLN3]=0; ks_{k2}=0; [BCK2]=0; WHI5T=0; [WHI5A]=0;
9	*GAL-CLN3*	Viable	MDT=150; f=0.48; D_{n3}=D_{n3}*20;
10	Multicopy *BCK2*	Viable	ks_{k2}=ks_{k2}*17;
11	*cln1*Δ *cln2*Δ	Viable	ks_{n2}=0; ks_{n2bf}=0; [CLN2]=0;
12	*cln1*Δ *cln2*Δ *bck2*Δ	Viable	ks_{n2}=0; ks_{n2bf}=0; [CLN2]=0; ks_{k2}=0; [BCK2]=0;
13	*cln1*Δ *cln2*Δ *sic1*Δ	Viable	ks_{n2}=0; ks_{n2bf}=0; [CLN2]=0; ks_{ki}=ks_{ki}*0.125; $ks_{ki,swi5}$=$ks_{ki,swi5}$*0.125; [CKIT]=0.2;
14	*cln1*Δ *cln2*Δ *cki*Δ	Viable	ks_{n2}=0; ks_{n2bf}=0; [CLN2]=0; ks_{ki}=0; $ks_{ki,swi5}$=0; [CKIT]=0; [CKIP]=0;
15	*cln1*Δ *cln2*Δ *GAL-SIC1*	Inviable	ks_{n2}=0; ks_{n2bf}=0; [CLN2]=0; MDT=150; f=0.48; ks_{ki}=ks_{ki}*33.33;
16	*cln1*Δ *cln2*Δ *GAL-CLN2*	Viable	ks_{n2bf}=0; MDT=150; f=0.48; ks_{n2}=0.15;
17	*cln1*Δ *cln2*Δ *GAL-SIC1 GAL-CLN2*	Viable	ks_{n2bf}=0; MDT=150; f=0.48; ks_{n2}=0.15; ks_{ki}=ks_{ki}*33.33;
18	*cln1*Δ *cln2*Δ *cdh1*Δ	Viable	ks_{n2}=0; ks_{n2bf}=0; [CLN2]=0; CDH1T=0; [CDH1A]=0;
19	*cln1*Δ *cln2*Δ *cdh1*Δ *GAL-SIC1*	Inviable	ks_{n2}=0; ks_{n2bf}=0; [CLN2]=0; CDH1T=0; [CDH1A]=0; MDT=150; f=0.48; ks_{ki}=ks_{ki}*33.33;
20	*cln1*Δ *cln2*Δ *cdh1*Δ *GAL-CLN2*	Viable	ks_{n2bf}=0; CDH1T=0; [CDH1A]=0; MDT=150; f=0.48; ks_{n2}=0.15;
21	*cln1*Δ *cln2*Δ *cdh1*Δ *GAL-SIC1 GAL-CLN2*	Viable	ks_{n2bf}=0; CDH1T=0; [CDH1A]=0; MDT=150; f=0.48; ks_{n2}=0.15; ks_{ki}=ks_{ki}*33.33;
22	*cln1*Δ *cln2*Δ *cln3*Δ	Inviable	ks_{n2}=0; ks_{n2bf}=0; [CLN2]=0; D_{n3}=0; [CLN3]=0;
23	*cln1*Δ *cln2*Δ *cln3*Δ *GAL-CLN2*	Viable	ks_{n2bf}=0; D_{n3}=0; [CLN3]=0; MDT=150; f=0.48; ks_{n2}=0.15;

Continued on next page

Table S6 – *Continued from previous page*

Phenotype #	Phenotype name	Viable/Inviable	Changes in WT parameters
24	*cln1Δ cln2Δ cln3Δ GAL-CLN3*	Viable	ks_{n2}=0; ks_{n2bf}=0; [CLN2]=0;<br>MDT=150; f=0.48; D_{n3}=D_{n3}*20;
25	*cln1Δ cln2Δ cln3Δ sicΔ*	Viable	ks_{n2}=0; ks_{n2bf}=0; [CLN2]=0;<br>D_{n3}=0; [CLN3]=0;<br>ks_{ki}=ks_{ki}*0.125; $ks_{ki,swi5}$=$ks_{ki,swi5}$*0.125;<br>[CKIT]=0.2;
26	*cln1Δ cln2Δ cln3Δ* multicopy *BCK2*	Viable	ks_{n2}=0; ks_{n2bf}=0; [CLN2]=0;<br>D_{n3}=0; [CLN3]=0;<br>ks_{k2}=ks_{k2}*17;
27	*cln1Δ cln2Δ cln3Δ bck2Δ GAL-CLN2*	Viable	ks_{n2bf}=0;<br>D_{n3}=0; [CLN3]=0;<br>ks_{k2}=0; [BCK2]=0;<br>MDT=150; f=0.48; ks_{n2}=0.15;
28	*cln1Δ cln2Δ cln3Δ* multicopy *CLB5*	Viable	ks_{n2}=0; ks_{n2bf}=0; [CLN2]=0;<br>D_{n3}=0; [CLN3]=0;<br>ks_{b5}=ks_{b5}=*5.33; $ks_{b5,bf}$=$ks_{b5,bf}$*5.33;
29	*cln1Δ cln2Δ cln3Δ GAL-CLB5*	Viable	ks_{n2}=0; ks_{n2bf}=0; [CLN2]=0;<br>D_{n3}=0; [CLN3]=0;<br>MDT=150; f=0.48; ks_{b5}=0.04;
30	*cln1Δ cln2Δ cln3Δ GAL-CLB2*	Inviable	ks_{n2}=0; ks_{n2bf}=0; [CLN2]=0;<br>D_{n3}=0; [CLN3]=0;<br>MDT=150; f=0.48; ks_{b2}=ks_{b2}*49.40;
31	*cln1Δ cln2Δ cln3Δ cdh1Δ*	Inviable	ks_{n2}=0; ks_{n2bf}=0; [CLN2]=0;<br>D_{n3}=0; [CLN3]=0;<br>CDH1T=0; [CDH1A]=0;
32	*cln1Δ cln2Δ cln3Δ apc-ts*	Inviable	ks_{n2}=0; ks_{n2bf}=0; [CLN2]=0;<br>D_{n3}=0; [CLN3]=0;<br>ks_{20}=0; $ks_{20,m1}$=0; CDH1T=0; [CDH1A]=0;
33	*cdh1Δ*	Viable	CDH1T=0; [CDH1A]=0;
34	*CDH1* constitutively active	Inviable	CDH1T=CDH1T*3; $ki_{h1,e}$=0;
35	*sic1Δ*	Viable	ks_{ki}=ks_{ki}*0.125; $ks_{ki,swi5}$=$ks_{ki,swi5}$*0.125;<br>[CKIT]=0.2;
36	*GAL-SIC1*	Viable	MDT=150; f=0.48; ks_{ki}=ks_{ki}*33.33;
37	*GAL-SIC1-dbΔ*	Inviable	MDT=150; f=0.48; ks_{ki}=ks_{ki}*33.33; kd_{kip}=0;
38	*sic1Δ cdc6Δ (ckiΔ)*	Viable	ks_{ki}=0; $ks_{ki,swi5}$=0; [CKIT]=0; [CKIP]=0;
39	*swi5Δ*	Viable	ks_{swi5}=0; $ks_{swi5,m1}$=0; [SWI5T]=0;
40	*sic1Δ cdc6Δ2-49 cdh1Δ*	Inviable	ks_{ki}=0; $ks_{ki,swi5}$=0; [CKIT]=0; [CKIP]=0;<br>CDH1T=0; [CDH1A]=0;
41	*swi5Δ cdh1Δ*	Inviable	ks_{swi5}=0; $ks_{swi5,m1}$=0; [SWI5T]=0;<br>CDH1T=0; [CDH1A]=0;
42	*swi5Δ cdh1Δ GAL-SIC1*	Viable	ks_{swi5}=0; $ks_{swi5,m1}$=0; [SWI5T]=0;<br>CDH1T=0; [CDH1A]=0;<br>MDT=150; f=0.48; ks_{ki}=ks_{ki}*33.33;
43	*clb5Δ clb6Δ*	Viable	ks_{b5}=0; $ks_{b5,bf}$=0; [CLB5T]=0;
44	*clb5Δ clb6Δ cln1Δ cln2Δ*	Inviable	ks_{b5}=0; $ks_{b5,bf}$=0; [CLB5T]=0;<br>ks_{n2}=0; ks_{n2bf}=0; [CLN2]=0;
45	*CLB5-dbΔ*	Viable	$kd_{b5,20}$=0; $kd_{b5,20,i}$=0;

Continued on next page

Table S6 – *Continued from previous page*

Phenotype #	Phenotype name	Viable/Inviable	Changes in WT parameters
46	*CLB5-db*Δ *sic1*Δ	Inviable	$kd_{b5,20}$=0; $kd_{b5,20,i}$=0; ks_{ki}=ks_{ki}*0.125; $ks_{ki,swi5}$=$ks_{ki,swi5}$*0.125; [CKIT]=0.2;
47	*GAL-CLB5*	Viable	MDT=150; f=0.48; ks_{b5}=0.04;
48	*GAL-CLB5 sic1*Δ	Inviable	MDT=150; f=0.48; ks_{b5}=0.04; ks_{ki}=ks_{ki}*0.125; $ks_{ki,swi5}$=$ks_{ki,swi5}$*0.125; [CKIT]=0.2;
49	*GAL-CLB5 cdh1*Δ	Inviable	MDT=150; f=0.48; ks_{b5}=0.04; CDH1T=0; [CDH1A]=0;
50	*GAL-CLB5-db*Δ	Inviable	MDT=150; f=0.48; ks_{b5}=0.04; $kd_{b5,20}$=0; $kd_{b5,20,i}$=0;
51	*clb1*Δ *clb2*Δ	Inviable	ks_{b2}=0; $ks_{b2,m1}$=0; [CLB2T]=0;
52	*clb1*Δ *clb2*Δ *clb5*Δ *clb6*Δ	Inviable	ks_{b2}=0; $ks_{b2,m1}$=0; [CLB2T]=0; ks_{b5}=0; $ks_{b5,bf}$=0; [CLB5T]=0;
53	*GAL-CLB2*	Viable	MDT=150; f=0.48; ks_{b2}=ks_{b2}*49.40;
54	Multicopy *GAL-CLB2*	Inviable	MDT=150; f=0.48; ks_{b2}=ks_{b2}*242.42;
55	*GAL-CLB2 sic1*Δ	Inviable	MDT=150; f=0.48; ks_{b2}=ks_{b2}*49.40; ks_{ki}=ks_{ki}*0.125; $ks_{ki,swi5}$=$ks_{ki,swi5}$*0.125; [CKIT]=0.2;
56	*GAL-CLB2 cdh1*Δ	Inviable	MDT=150; f=0.48; ks_{b2}=ks_{b2}*49.40; CDH1T=0; [CDH1A]=0;
57	*GAL-CLB2 swi5*Δ	Inviable	MDT=150; f=0.48; ks_{b2}=ks_{b2}*49.40; ks_{swi5}=0; $ks_{swi5,m1}$=0; [SWI5T]=0;
58	*CLB2-db*Δ	Inviable	$kd_{b2,20}$=0; $kd_{b2,h1}$=$kd_{b2,h1}$*0.09; $kd_{b2,20,i}$=0;
59	*CLB2-db*Δ in galactose	Inviable	$kd_{b2,20}$=0; $kd_{b2,h1}$=$kd_{b2,h1}$*0.09; $kd_{b2,20,i}$=0; MDT=150; f=0.48;
60	*CLB2-db*Δ *GAL-SIC1*	Viable	$kd_{b2,20}$=0; $kd_{b2,h1}$=$kd_{b2,h1}$*0.09; $kd_{b2,20,i}$=0; MDT=150; f=0.48; ks_{ki}=ks_{ki}*33.33;
61	*CLB2-db*Δ multicopy *SIC1*	Viable	$kd_{b2,20}$=0; $kd_{b2,h1}$=$kd_{b2,h1}$*0.09; $kd_{b2,20,i}$=0; ks_{ki}=ks_{ki}*65; $ks_{ki,swi5}$=$ks_{ki,swi5}$*65;
62	*CLB2-db*Δ *clb5*Δ *clb6*Δ	Inviable	$kd_{b2,20}$=0; $kd_{b2,h1}$=$kd_{b2,h1}$*0.09; $kd_{b2,20,i}$=0; ks_{b5}=0; $ks_{b5,bf}$=0; [CLB5T]=0;
63	*CLB2-db*Δ *clb5*Δ *clb6*Δ in galactose	Viable	$kd_{b2,20}$=0; $kd_{b2,h1}$=$kd_{b2,h1}$*0.09; $kd_{b2,20,i}$=0; ks_{b5}=0; $ks_{b5,bf}$=0; [CLB5T]=0; MDT=150; f=0.48;
64	*GAL-CLB2-db*Δ	Inviable	$kd_{b2,20}$=0; $kd_{b2,h1}$=$kd_{b2,h1}$*0.09; $kd_{b2,20,i}$=0; MDT=150; f=0.48; ks_{b2}=ks_{b2}*49.40;
65	*CLB1 clb2*Δ	Viable	ks_{b2}=ks_{b2}*0.33; $ks_{b2,m1}$=$ks_{b2,m1}$*0.33;
66	*CLB1 clb2*Δ *cdh1*Δ	Inviable	ks_{b2}=ks_{b2}*0.33; $ks_{b2,m1}$=$ks_{b2,m1}$*0.33; CDH1T=0; [CDH1A]=0;
67	*CLB1 clb2*Δ *pds1*Δ	Inviable	ks_{b2}=ks_{b2}*0.33; $ks_{b2,m1}$=$ks_{b2,m1}$*0.33; ks_{pds}=0; [PDS1T]=0;
68	*cdc20-ts* (*cdc20*Δ)	Inviable	ks_{20}=0; $ks_{20,m1}$=0; [CDC20T]=0; [CDC20A-APCP]=0;
69	*clb5*Δ *clb6*Δ *cdc20*Δ	Inviable	ks_{b5}=0; $ks_{b5,bf}$=0; [CLB5T]=0; ks_{20}=0; $ks_{20,m1}$=0; [CDC20T]=0; [CDC20A-APCP]=0;
70	*cdc20*Δ *pds1*Δ	Inviable	ks_{20}=0; $ks_{20,m1}$=0; [CDC20T]=0; [CDC20A-APCP]=0; ks_{pds}=0; [PDS1T]=0;
71	*clb5*Δ *clb6*Δ *cdc20*Δ *pds1*Δ	Viable	ks_{b5}=0; $ks_{b5,bf}$=0; [CLB5T]=0;

Continued on next page

Table S6 – *Continued from previous page*

Phenotype #	Phenotype name	Viable/Inviable	Changes in WT parameters
			ks_{20}=0; $ks_{20,m1}$=0; [CDC20T]=0; [CDC20A-APCP]=0; ks_{pds}=0; [PDS1T]=0;
72	*CLB5-db*Δ *cdc20*Δ *pds1*Δ	Inviable	$kd_{b5,20}$=0; $kd_{b5,20,i}$=0; ks_{20}=0; $ks_{20,m1}$=0; [CDC20T]=0; [CDC20A-APCP]=0; ks_{pds}=0; [PDS1T]=0;
73	*CLB5-db*Δ *pds1*Δ	Viable	$kd_{b5,20}$=0; $kd_{b5,20,i}$=0; ks_{pds}=0; [PDS1T]=0;
74	*GAL-CDC20*	Inviable	MDT=150; f=0.48; ks_{20}=ks_{20}*1666.67;
75	*GALL-CDC20 sic1*Δ *cdh1*Δ	Viable	MDT=150; f=0.48; ks_{20}=ks_{20}*1000; ks_{ki}=ks_{ki}*0.125; $ks_{ki,swi5}$=$ks_{ki,swi5}$*0.125; [CKIT]=0.2; CDH1T=0; [CDH1A]=0;
76	*GALL-CDC20 sic1*Δ *Cdc6*Δ*2-49 cdh1*Δ	Viable	MDT=150; f=0.48; ks_{20}=ks_{20}*1000; ks_{ki}=0; $ks_{ki,swi5}$=0; [CKIT]=0; [CKIP]=0; CDH1T=0; [CDH1A]=0;
77	*APC-A*	Viable	$ka_{cp,b2}$=0;
78	*APC-A sic1*Δ	Viable	$ka_{cp,b2}$=0; ks_{ki}=ks_{ki}*0.125; $ks_{ki,swi5}$=$ks_{ki,swi5}$*0.125; [CKIT]=0.2;
79	*APC-A sic1*Δ *cdc6*Δ*2-49*	Viable	$ka_{cp,b2}$=0; ks_{ki}=0; $ks_{ki,swi5}$=0; [CKIT]=0; [CKIP]=0;
80	*APC-A cdh1*Δ	Inviable	$ka_{cp,b2}$=0; CDH1T=0; [CDH1A]=0;
81	*APC-A cdh1*Δ in galactose	Inviable	$ka_{cp,b2}$=0; CDH1T=0; [CDH1A]=0; MDT=150; f=0.48;
82	*APC-A cdh1*Δ *GAL-SIC1*	Viable	$ka_{cp,b2}$=0; CDH1T=0; [CDH1A]=0; MDT=150; f=0.48; ks_{ki}=ks_{ki}*33.33;
83	*APC-A cdh1*Δ multicopy *SIC1*	Viable	$ka_{cp,b2}$=0; CDH1T=0; [CDH1A]=0; ks_{ki}=ks_{ki}*65; $ks_{ki,swi5}$=$ks_{ki,swi5}$*65;
84	*APC-A cdh1*Δ multicopy *CDC20*	Viable	$ka_{cp,b2}$=0; CDH1T=0; [CDH1A]=0; ks_{20}=ks_{20}*2; $ks_{20,m1}$=$ks_{20,m1}$*2;
85	*APC-A GAL-CLB2*	Inviable	$ka_{cp,b2}$=0; MDT=150; f=0.48; ks_{b2}=ks_{b2}*49.40;
86	*pds1*Δ	Viable	ks_{pds}=0; [PDS1T]=0;
87	*PDS1-db*Δ	Inviable	$kd_{pds,20}$=0;
88	*GAL-PDS1-db*Δ	Inviable	MDT=150; f=0.48; ks_{pds}=ks_{pds}*3.33; $kd_{pds,20}$=0;
89	*esp1-ts*	Inviable	ESP1T=0;
90	*GAL-PDS1-db*Δ *esp1-ts*	Inviable	MDT=150; f=0.48; ks_{pds}=ks_{pds}*3.33; $kd_{pds,20}$=0; ESP1T=0;
91	*GAL-ESP1 cdc20-ts*	Inviable	MDT=150; f=0.48; ESP1T=ESP1T*2; ks_{20}=0; $ks_{20,m1}$=0; [CDC20T]=0; [CDC20A-APCP]=0;
92	*ppx*Δ	Viable	PPXT=0; [PPX]=0;
93	*GAL-PPX*	Viable	MDT=150; f=0.48; PPXT=PPXT*2;
94	*tem1*Δ	Inviable	TEM1T=0; [TEM1]=0;
95	*net1-ts*	Viable	kas_{net}=kas_{net}*0.45;

Continued on next page

Table S6 – *Continued from previous page*

Phenotype #	Phenotype name	Viable/Inviable	Changes in WT parameters
96	*tem1Δ net1-ts*	Viable	TEM1T=0; [TEM1]=0; kas_{net}=kas_{net}*0.45;
97	*GAL-TEM1*	Viable	TEM1T=TEM1T*5;
98	*tem1-ts GAL-CDC15*	Viable	TEM1T=0; [TEM1]=0; MDT=150; f=0.48; CDC15T=CDC15T*10;
99	*tem1-ts* multicopy *CDC14*	Viable	TEM1T=0; [TEM1]=0; CDC14T=CDC14T*2;
100	Multicopy *CDC15*	Viable	CDC15T=CDC15T*20;
101	*tem1-ts* multicopy *CDC15*	Viable	TEM1T=0; [TEM1]=0; CDC15T=CDC15T*20;
102	*net1-ts cdc20-ts*	Inviable	kas_{net}=kas_{net}*0.45; ks_{20}=0; $ks_{20,m1}$=0; [CDC20T]=0; [CDC20A-APCP]=0;
103	*cdc15Δ*	Inviable	CDC15T=0;
104	*cdc15Δ net1-ts*	Viable	CDC15T=0; kas_{net}=kas_{net}*0.45;
105	*cdc15Δ net1-ts cdh1Δ*	Viable	CDC15T=0; kas_{net}=kas_{net}*0.45; CDH1T=0; [CDH1A]=0;
106	*cdc15-ts* multicopy *TEM1*	Inviable	CDC15T=0; TEM1T=TEM1T*5;
107	*cdc15-ts* multicopy *CDC14*	Viable	CDC15T=0; CDC14T=CDC14T*2;
108	*TAB6-1*	Viable	kas_{net}=kas_{net}*0.5;
109	*cdc15Δ TAB6-1*	Viable	CDC15T=0; kas_{net}=kas_{net}*0.5;
110	*TAB6-1 clb5Δ clb6Δ*	Inviable	kas_{net}=kas_{net}*0.5; ks_{b5}=0; $ks_{b5,bf}$=0; [CLB5T]=0;
111	*TAB6-1 CLB1 clb2Δ*	Viable	kas_{net}=kas_{net}*0.5; ks_{b2}=ks_{b2}*0.33; $ks_{b2,m1}$=$ks_{b2,m1}$*0.33;
112	*cdc14-ts*	Inviable	CDC14T=0;
113	*cdc14-ts sic1Δ*	Inviable	CDC14T=0; ks_{ki}=ks_{ki}*0.125; $ks_{ki,swi5}$=$ks_{ki,swi5}$*0.125; [CKIT]=0.2;
114	*cdc14-ts cdh1Δ*	Inviable	CDC14T=0; CDH1T=0; [CDH1A]=0;
115	*cdc14-ts GAL-SIC1*	Inviable	CDC14T=0; MDT=150; f=0.48; ks_{ki}=ks_{ki}*33.33;
116	*cdc14-ts GAL-CLN2*	Inviable	CDC14T=0; MDT=150; f=0.48; ks_{n2}=0.15;
117	*GAL-NET1*	Inviable	MDT=150; f=0.48; NET1T=NET1T*10.85;
118	*GAL-CDC14*	Inviable	MDT=150; f=0.48; CDC14T=CDC14T*7;
119	*GAL-NET1 GAL-CDC14*	Viable	MDT=150; f=0.48; CDC14T=CDC14T*7; NET1T=NET1T*10.85;

Table S7: **111 phenotypes that are captured by the feasible parameter vectors (Training Set)**

Phenotype number	Phenotype name	Phenotype number	Phenotype name
1	WT in glucose (Viable)	61	*CLB2-db*Δ multicopy *SIC1* (Viable)
2	WT in galactose (Viable)	62	*CLB2-db*Δ *clb5*Δ *clb6*Δ (Inviable)
3	*cln3*Δ (Viable)	63	*CLB2-db*Δ *clb5*Δ *clb6*Δ in galactose (Viable)
4	*bck2*Δ (Viable)	64	*GAL-CLB2-db*Δ (Inviable)
5	*cln3*Δ *bck2*Δ (Inviable)	65	*CLB1 clb2*Δ (Viable)
6	*cln3*Δ *bck2*Δ multicopy *CLN2* (Inviable)	68	*cdc20-ts* (*cdc20*Δ) (Inviable)
7	*cln3*Δ *bck2*Δ *sic*Δ (Inviable)	69	*clb5*Δ *clb6*Δ *cdc20*Δ (Inviable)
8	*cln3*Δ *bck2*Δ *whi5*Δ (Viable)	70	*cdc20*Δ *pds1*Δ (Inviable)
9	*GAL-CLN3* (Viable)	71	*clb5*Δ *clb6*Δ *cdc20*Δ *pds1*Δ (Viable)
10	Multicopy *BCK2* (Viable)	72	*CLB5-db*Δ *cdc20*Δ *pds1*Δ (Inviable)
11	*cln1*Δ *cln2*Δ (Viable)	73	*CLB5-db*Δ *pds1*Δ (Viable)
13	*cln1*Δ *cln2*Δ *sic1*Δ (Viable)	75	*GALL-CDC20 sic1*Δ *cdh1*Δ (Viable)
14	*cln1*Δ *cln2*Δ *cki*Δ (Viable)	76	*GALL-CDC20 sic1*Δ *Cdc6*Δ*2-49 cdh1*Δ (Viable)
15	*cln1*Δ *cln2*Δ *GAL-SIC1* (Inviable)	77	*APC-A* (Viable)
16	*cln1*Δ *cln2*Δ *GAL-CLN2* (Viable)	78	*APC-A sic1*Δ (Viable)
17	*cln1*Δ *cln2*Δ *GAL-SIC1 GAL-CLN2* (Viable)	79	*APC-A sic1*Δ *cdc6*Δ*2-49* (Viable)
18	*cln1*Δ *cln2*Δ *cdh1*Δ (Viable)	80	*APC-A cdh1*Δ (Inviable)
19	*cln1*Δ *cln2*Δ *cdh1*Δ *GAL-SIC1* (Inviable)	81	*APC-A cdh1*Δ in galactose (Inviable)
20	*cln1*Δ *cln2*Δ *cdh1*Δ *GAL-CLN2* (Viable)	82	*APC-A cdh1*Δ *GAL-SIC1* (Viable)
21	*cln1*Δ *cln2*Δ *cdh1*Δ *GAL-SIC1 GAL-CLN2* (Viable)	83	*APC-A cdh1*Δ multicopy *SIC1* (Viable)
22	*cln1*Δ *cln2*Δ *cln3*Δ (Inviable)	84	*APC-A cdh1*Δ multicopy *CDC20* (Viable)
23	*cln1*Δ *cln2*Δ *cln3*Δ *GAL-CLN2* (Viable)	85	*APC-A GAL-CLB2* (Inviable)
24	*cln1*Δ *cln2*Δ *cln3*Δ *GAL-CLN3* (Viable)	86	*pds1*Δ (Viable)
25	*cln1*Δ *cln2*Δ *cln3*Δ *sic*Δ (Viable)	87	*PDS1-db*Δ (Inviable)
26	*cln1*Δ *cln2*Δ *cln3*Δ multicopy *BCK2* (Viable)	88	*GAL-PDS1-db*Δ (Inviable)
27	*cln1*Δ *cln2*Δ *cln3*Δ *bck2*Δ *GAL-CLN2* (Viable)	89	*esp1-ts* (Inviable)
28	*cln1*Δ *cln2*Δ *cln3*Δ multicopy *CLB5* (Viable)	90	*GAL-PDS1-db*Δ *esp1-ts* (Inviable)
29	*cln1*Δ *cln2*Δ *cln3*Δ *GAL-CLB5* (Viable)	91	*GAL-ESP1 cdc20-ts* (Inviable)
30	*cln1*Δ *cln2*Δ *cln3*Δ *GAL-CLB2* (Inviable)	92	*ppx*Δ (Viable)
31	*cln1*Δ *cln2*Δ *cln3*Δ *cdh1*Δ (Inviable)	93	*GAL-PPX* (Viable)
32	*cln1*Δ *cln2*Δ *cln3*Δ *apc-ts* (Inviable)	94	*tem1*Δ (Inviable)
33	*cdh1*Δ (Viable)	95	*net1-ts* (Viable)
34	*CDH1* constitutively active (Inviable)	96	*tem1*Δ *net1-ts* (Viable)
35	*sic1*Δ (Viable)	97	*GAL-TEM1* (Viable)
36	*GAL-SIC1* (Viable)	98	*tem1-ts GAL-CDC15* (Viable)
37	*GAL-SIC1-db*Δ (Inviable)	99	*tem1-ts* multicopy *CDC14* (Viable)
38	*sic1*Δ *cdc6*Δ *(cki*Δ*)* (Viable)	100	Multicopy *CDC15* (Viable)
39	*swi5*Δ (Viable)	101	*tem1-ts* multicopy *CDC15* (Viable)
40	*sic1*Δ *cdc6*Δ*2-49 cdh1*Δ (Inviable)	102	*net1-ts cdc20-ts* (Inviable)
41	*swi5*Δ *cdh1*Δ (Inviable)	103	*cdc15*Δ (Inviable)
42	*swi5*Δ *cdh1*Δ *GAL-SIC1* (Viable)	104	*cdc15*Δ *net1-ts* (Viable)
43	*clb5*Δ *clb6*Δ (Viable)	105	*cdc15*Δ *net1-ts cdh1*Δ (Viable)
44	*clb5*Δ *clb6*Δ *cln1*Δ *cln2*Δ (Inviable)	106	*cdc15-ts* multicopy *TEM1* (Inviable)
45	*CLB5-db*Δ (Viable)	107	*cdc15-ts* multicopy *CDC14* (Viable)
47	*GAL-CLB5* (Viable)	108	*TAB6-1* (Viable)
49	*GAL-CLB5 cdh1*Δ (Inviable)	109	*cdc15*Δ *TAB6-1* (Viable)
50	*GAL-CLB5-db*Δ (Inviable)	110	*TAB6-1 clb5*Δ *clb6*Δ (Inviable)
51	*clb1*Δ *clb2*Δ (Inviable)	111	*TAB6-1 CLB1 clb2*Δ (Viable)
52	*clb1*Δ *clb2*Δ *clb5*Δ *clb6*Δ (Inviable)	112	*cdc14-ts* (Inviable)
53	*GAL-CLB2* (Viable)	113	*cdc14-ts sic1*Δ (Inviable)
54	Multicopy *GAL-CLB2* (Inviable)	114	*cdc14-ts cdh1*Δ (Inviable)
56	*GAL-CLB2 cdh1*Δ (Inviable)	115	*cdc14-ts GAL-SIC1* (Inviable)
57	*GAL-CLB2 swi5*Δ (Inviable)	116	*cdc14-ts GAL-CLN2* (Inviable)
58	*CLB2-db*Δ (Inviable)	118	*GAL-CDC14* (Inviable)
59	*CLB2-db*Δ in galactose (Inviable)	119	*GAL-NET1 GAL-CDC14* (Viable)
60	*CLB2-db*Δ *GAL-SIC1* (Viable)		

A parameter vector satisfies the first feasibility criterion (FC_1) if it captures the phenotypes on this list.

Table S8: **Eight phenotypes that are not captured by the feasible parameter vectors (Training Set)**

Phenotype #	Phenotype name
12	*cln1*Δ *cln2*Δ *bck2*Δ (Viable)
46	*CLB5-db*Δ *sic1*Δ (Inviable)
48	*GAL-CLB5 sic1*Δ (Inviable)
55	*GAL-CLB2 sic1*Δ (Inviable)
66	*CLB1 clb2*Δ *cdh1*Δ (Inviable)
67	*CLB1 clb2*Δ *pds1*Δ (Inviable)
74	*GAL-CDC20* (Inviable)
117	*GAL-NET1* (Inviable)

The model is not able to capture these phenotypes without losing some of the remaining 111 phenotypes due to competition between phenotypes. Hence, phenotypes on this list are excluded from the first feasibility criterion (FC_1).

Table S9: **Statistics regarding the parameter ranges depicted in Figure S1**

Distribution in Figure S1	The range of max/min values	The average of max/min values	The standard deviation of max/min values
Blue line	1.0000–1.0650	1.0056	0.0106
Green line	1.0035–1.2897	1.0813	0.0528
Red line	1.0124–1.3601	1.1209	0.0737

Statistics from the distributions shown in Figure S1. max/min is the magnitude of each parameter range, which is computed as the ratio of the parameter's maximum and minimum values among a given parameter vector population.

Table S10: **Statistics regarding the parameter ranges depicted in Figure S3**

Distribution in Figure S3	The range of max/min values	The average of max/min values	The standard deviation of max/min values
Blue line	1.0000–1.0654	1.0056	0.0106
Green line	1.0007–1.2126	1.0340	0.0349
Red line	1.0124–1.3601	1.1209	0.0737

Statistics from the distributions shown in Figure S3. max/min is the magnitude of each parameter range, which is computed as the ratio of the parameter's maximum and minimum values among a given parameter vector population.

Table S11: **Statistics regarding the distributions of CV values depicted in Figure 6a**

Distribution in Figure 6a	The range of CV values	The average of CV values	The standard deviation of CV values
Blue line	0.0037–0.1834	0.0246	0.0151
Green line	0.0090–0.5126	0.0549	0.0381
Red line	0.0090–0.5260	0.0643	0.0450

Statistics from the distributions shown in Figure 6a. CV values belong to relative abundance predictions (details specified in the caption of Figure 6).

Table S12: **Statistics regarding the distributions of CV values depicted in Figure 7a**

Distribution in Figure 7a	The range of CV values	The average of CV values	The standard deviation of CV values
Blue line	0.0093–0.4993	0.0572	0.0350
Green line	0.0093–0.5260	0.0639	0.0452
Red line	0.0090–0.5260	0.0659	0.0465

Statistics from the distributions shown in Figure 7a. CV values belong to relative abundance predictions (details specified in the caption of Figure 7).

Table S13: **Statistics regarding the distributions of CV values depicted in Figure 7h**

Distribution in Figure 7h	The range of CV values	The average of CV values	The standard deviation of CV values
Blue line	0.0133–0.4066	0.1234	0.0675
Green line	0.0096–0.2131	0.0553	0.0294
Red line	0.0133–0.4993	0.0862	0.0699

Statistics from the distributions shown in Figure 7h. CV values belong to relative abundance predictions (details specified in the caption of Figure 7).
