# Supplementary material for: Predicting network modules of cell cycle regulators using relative protein abundance statistics
Source: BMC Syst Biol. 2017 Feb 28;11:30. doi: 10.1186/s12918-017-0409-1 (PMC5329933; doi:10.1186/s12918-017-0409-1)
Supplement: Additional file 2 — Supplementary Text. This pdf file includes detailed descriptions of certain aspects of our study including the computation of the estimated volume spanned by an ensemble of parameter vectors (Section 1), using LHS for generating an ensemble of parameter vectors (Section 2), selection of the initial DE population that spans a large volume (Section 3), computation of the estimated volume spanned by the most recent subensemble of parameter vectors (Section 4), selection of the initial DE population that spans a large volume and has a large prediction range (Section 5), alternative parameter space exploration methods (Section 6), the impact of precision on the number of identified feasible parameter vectors (Section 7), the impact of additional normalization on the contributions of individual parameters to the feasible region’s volume (Section 8), the impact of additional normalization on the contributions of individual parameters to the robustness score (Section 9), the impact of the viability criteria on the model prediction range (Section 10), the choice of ODE solver (Section 11), a potential biological application of the parameter space exploration approach (Section 12), and discussion regarding the most critical model parameters (Section 13), and the most fragile phenotypes (Section 14). (PDF 350 kb) [file 12918_2017_409_MOESM2_ESM.pdf]

Supplementary Text

1. Computation of the estimated volume spanned by an ensemble of parameter vectors

In this study, prior to collecting statistics regarding predictions and robustness of parameter vectors from a DE run, we collect all the generated trial vectors that capture the 111 phenotypes listed in Table S7 out of the 119 total phenotypes (Table S6). Some of these trial vectors become parent vectors depending on the particular criteria enforced during a given DE run. However, the performance of a DE run is assessed based on the complete collection of feasible parameter vectors it produces regardless of the parent status of the individual vectors. With m as the total number of parameter vectors and n as the vector dimension (total number of parameters), the collection of these parameter vectors form the $m \times n$ ensemble matrix

$$\mathbf{X} = \begin{bmatrix} x_1^{(1)} & x_2^{(1)} & \cdots & x_n^{(1)} \\ x_1^{(2)} & x_2^{(2)} & \cdots & x_n^{(2)} \\ \vdots & \vdots & \ddots & \vdots \\ x_1^{(m)} & x_2^{(m)} & \cdots & x_n^{(m)} \end{bmatrix}, \tag{1}$$

where $x_j^{(i)}$ is the value of the jth parameter in the ith parameter vector in the ensemble. Then, we identify the minimum and maximum values of each parameter j, $1 \leq j \leq m$, in the ensemble $\mathbf{X}$,

$$\hat{x}_j = \min_{1 \leq i \leq m} x_j^{(i)}, \tag{2}$$

$$\breve{x}_j = \max_{1 \leq i \leq m} x_j^{(i)}. \tag{3}$$

The $\log_{10}$ volume of the ensemble is estimated by

$$V(\mathbf{X}) = \sum_{j=1, \hat{x}_j \neq \breve{x}_j}^{n} \log_{10}(\breve{x}_j - \hat{x}_j). \tag{4}$$

Note that the volume encompassed by the ensemble of m-dimensional vectors $x^{(i)}$ could be computed exactly by finding a Delaunay triangulation of the ensemble into m-simplices, and then computing the exact volumes of the simplices. This is a nontrivial computation, though, compared to $V(\mathbf{X})$.

2. Generating Ensemble 2 with Latin hypercube sampling (LHS)

Latin hypercube sampling (LHS) is a method [1] for generating a sample of parameter vectors from a multidimensional box.

- Let $x_j^{(i)}$ represent the value of the jth parameter in the ith parameter vector of Ensemble 1 (3146 vectors that capture the 111 phenotypes in Table S7) out of all 119 phenotypes. Each parameter vector stores 152 (126 kinetic parameters and 26 initial conditions) values.

- We sample for 50000 parameter vectors. The range of each parameter in Ensemble 1($[\hat{x}_j,\ \breve{x}_j]$ is the range for the jth parameter) is divided into $50,000$ mutually exclusive subintervals.

- One value is randomly selected from each subinterval in $[\hat{x}_1,\ \breve{x}_1]$ for the first parameter x_1. Hence, 50000 values are sampled: $x_{1,1}$ through $x_{1,50000}$. These values are paired with 50000 values sampled for the second parameter x_2 randomly without replacement, which means that each x_2 value is paired with only one value of x_1.

- Then, these 50000 pairs are combined with 50000 values of x_3, once again without replacement and 50000 triplets are obtained.

- This procedure is continued until 50000 152-tuples are generated.

LHS provides a fairly uniform coverage of each parameter range by dividing each range into 50000 intervals (or number of samples). Out of the 50000 LH samples, only 243 capture the 111 phenotypes in Table S7 out of all 119 phenotypes. These 243 feasible parameter vectors form Ensemble 2 (Table 2).

3. Selection of the initial DE population (from Ensemble 1) that spans a large volume in the parameter space

The following steps describe how we select a set of parameter vectors with a large spread (volume-wise) in the parameter space. This is done to ensure diversity in the parameter values among the initial DE population before we start searching for more feasible parameter vectors.

- Randomly select a parameter vector $x^{(r_1)}$ (row r_1) from Ensemble 1 ($\mathbf{X_{E1}}$).

- Identify the parameter vector $x^{(r_2)}$ (row r_2) that has the largest distance from $x^{(r_1)}$. Select this vector as the second member of the initial DE population.

  The distance squared between two vectors $x^{(r_1)}$ and $x^{(i)}$ in the ensemble is computed as

$$\sum_{j=1}^{n} \frac{(x_j^{(i)} - x_j^{(r_1)})^2}{\overline{x}_j^2}\,, \tag{5}$$

  where the average value of the jth parameter among the vectors in Ensemble $\mathbf{X_{E1}}$ is

$$\overline{x}_j = \sum_{i=1}^{m} \frac{x_j^{(i)}}{m}\,. \tag{6}$$

- Identify the parameter vector $x^{(r_3)}$ that has the largest sum of squared distances from $x^{(r_1)}$ and $x^{(r_2)}$. Select this vector as the third member of the initial DE population.

- Continue this procedure until 19 parameter vectors are collected as the initial DE population. This population will then be the starting point for exploring the feasible region through evolutionary operations of DE in order to discover new feasible vectors different from the parameter vectors in Ensemble 1.

This algorithm is a greedy heuristic that may not find the 19 ensemble points whose 18-simplex with the maximum volume (which is likely an NP hard problem). While selecting the initial DE population using this scheme described above, we use two distinct groups of parameters for computing the squared distances between parameter vectors. This is done to assess the effects of the choice between two groups on the performance of DE. The first group of parameters includes only the most critical ten parameters (listed in Table 3). The second group (123 parameters) includes all the parameters in Table S1 except the three parameters that are fixed during DE runs (MDT, f, and ks_{n2}). We do not take into account the additional 26 parameters representing the initial conditions of WT simulations since the values of these parameters do not affect the number of phenotypes captured by the model [14].

4. Computation of the estimated volume spanned by the most recent subensemble of parameter vectors

Figure 1 shows the dynamic evolution of the estimated volume that is spanned by subensembles of 19 feasible parameter vectors. Here, 19 is the population size of DE. Per ensemble, prior to plotting the evolution of estimated volume spanned by its subensembles, these subensembles are ordered with respect to their time appearance in DE. The first subensemble includes vectors 1 to 19, second ensemble includes vectors 2 to 20, third ensemble includes vectors 3 to 21, and so on until the last vector of the most recent subensemble coincides with the last vector in the complete ensemble. The true volume is zero, since 19 points in 152 dimensions lie in a hyperplane, which has zero volume.

5. Selection of the initial DE population that spans a large volume and has a large prediction range

3146 feasible parameter vectors from the initial DE optimization run (Ensemble 1) generate 30 unique phenotypic prediction vectors for the 129 novel mutants. 17 of these 30 vectors derive from more than one parameter vector in Ensemble 1. We expect these 17 prediction vectors to be more robust against the mutation and crossover operations of DE compared to the remaining 13 since they dominate the phenotype

space of the novel mutants. Hence, when we are generating an initial DE population with a large range of predictions (phenotypic diversity of novel mutants) in the population, we select parameter vectors predicting each of these 17 vectors while simultaneously increasing the spread of the selected population (volume-wise) in the parameter space. In order to meet these two objectives, we follow the steps below.

- Run the simulations for all the novel phenotypes with the parameter vectors in Ensemble 1. Label each parameter vector with a number corresponding to the unique prediction vector it generates.
- Identify the prediction vectors that are derived from more than one parameter vector. Create a set of the parameter vectors that generate each such prediction vector. Randomly select a vector $x^{(r_1)}$ from the first set.
- Identify the parameter vector $x^{(r_2)}$ in the second group (generating the second unique prediction vector) that has the largest distance from $x^{(r_1)}$, and select this vector as the second member of the initial DE population. As before, the distance is a weighted 2-norm, where the weights are the squares of the parameter ensemble averages.
- Identify the parameter vector $x^{(r_3)}$ from the third set of vectors that has the largest sum of squared distances from $x^{(r_1)}$ and $x^{(r_2)}$, and select this vector as the third member of the initial DE population.
- Continue this procedure until the number of required parameter vectors to form the initial DE population is reached. In case the number of sets represented by more than one parameter vector is less than the target population size, fill the remaining slots by performing the same type of distance maximization regardless of the prediction vector generated by the parameter vectors. The resulting population of parameter vectors serves as the starting point for exploring the feasible region by DE for discovering new feasible vectors.

6. Alternative parameter space exploration methods

Here, we explain the rationale behind our method of choice for exploring our model's parameter space, namely DE, over three alternatives simulated annealing (SA), random walk (RW), and Monte-Carlo (MC) based sampling methods.

During the initial phases of this study, we implemented a random walk strategy previously published in [2] for sampling the feasible region of our cell cycle model. Here, random parameter vectors in the feasible region are selected and per vector, all parameters are simultaneously perturbed by adding values from a

Gaussian distribution with zero mean and a tuned standard deviation value. With this strategy, we tried a range of values (with small/large values new parameter vectors are always/never feasible), the efficiency of sampling was well below the randomized Latin hypercube sampling (LHS) for both the initial optimization stage (maximizing the number of correctly simulated mutants in S6) as well as the exploration of the feasible region for novel predictions.

Next, we considered another sampling method ,namely simulated annealing (SA), for our model. SA is a stochastic algorithm [3] that pseudorandomly generates points in the neighborhood of an initial guess until it finds a new feasible point. The algorithm replaces the former guess with this new point with a certain probability if the objective function is improved. This process is continued until convergence. The probability value for accepting a new feasible point as the starting point of a new iteration depends on a variable (called "temperature") that is lowered throughout the search using a temperature schedule. As T decreases throughout the search, the search becomes more greedy and performs like a gradient descent method. In a recent study from our lab [4], the performance of SA was found to be significantly lower than DE for our model when we attempted to optimize the number of correctly simulated cell cycle mutants based on the experimental viability constraints in Table S6. Specifically, SA improved the number of captured phenotypes to 90 (from 72 captured phenotypes generated by the initial guess) compared to 111 correctly simulated mutants obtained by implementing DE. Hence, we did not use SA for exploring the feasible region of our model in this study.

We also considered direct Monte-Carlo (MC) based sampling, which has previously been used in exploring the feasible region of model parameter spaces in systems biology [5], for exploring the feasible region of our model. This approach is typically effective in fairly low dimensional parameter spaces (e.g., three model parameters are varied while performing MC sampling in [5]), whereas LHS is a more practical sampling approach for high-dimensional parameter spaces due to its ability to generate stratified random samples. With MC sampling, the number of samples needed to uniformly cover a parameter space quickly reaches very high values (e.g., k^n samples for n model parameters and k uniformly selected values per parameter). LHS circumvents this problem by allowing a single value to be included from each parameter subinterval (i.e. number of subintervals equal to the number of samples in LHS), without requiring k^n samples, but only k "stratified" samples to represent the parameter space with a fairly uniform coverage more efficiently. Nevertheless, for our model with a total of 152 varying parameters, LHS proves to be ineffective presumably due to high dimensionality of the parameter space, large number of phenotypic constraints that need to be simultaneously satisfied (phenotypes of 111 cell cycle mutants), and the of the model nonlinearity (e.g.,

sigmoid functions describing the activation/inactivation of several model species). By selecting a limited population of samples (due to the computational cost of running DE for many generations) while maximizing the volume spanned by this population (i.e. the starting population of DE), and then implementing DE for hundreds of generations for exploring the feasible region of our model, we are able to start with a fairly uniform coverage of the known part of the feasible region initially. Then, we gradually expand the model's prediction range through evolutionary sampling by using multiple feasibility criteria. Finally, we would like to note that, given the widely accepted "no-free lunch theorem of optimization" [6, 7], conclusions regarding the effectiveness of our approach to parameter space exploration are based on its application to our particular 152-dimensional model of the cell cycle and is not proposed as a universally effective approach. Hence, we focus on our conclusions regarding the interplay between the structure of the regulatory network and the predictive variance values associated with the network components, as well as the machine learning based models that are able to predict biological functions (or network modules) of model species using relative protein abundance statistics.

7. The impact of precision on the number of identified feasible parameter vectors

The criteria of feasibility in terms of the cell size at subsequent divisions and the maximum cell size in a simulation is described by the following: If the cell size exceeds 25 (arbitrary units) at any time during the simulation of a novel strain, its phenotype is inviable. Otherwise, if the cell size at the last division is within 5% of the cell sizes at the two previous divisions, the phenotype is viable. Finally, if the model generates multiple period cycles with the size at division oscillating between values that differ by more than 5%, the phenotype is neither viable nor inviable.

In order look at the impact of precision on the number of feasible parameter vectors, we randomized the precision of the parameter values and checked for feasibility. We used the group 3415 parameter vectors of which 3146 (Ensemble 1) were feasible with the single precision constraint. We first verified that the number of digits (beyond decimal points) used by Matlab with double precision as 15. Next, we randomized this number between 4 and 15 and identified 3011 vectors as feasible (out of the 3415) with random precision. The overlap (common parameter vectors) between Ensemble 1 and these 3011 vectors was 2809. In other words, 93.3% (2809/3011) of the new feasible ensemble was contained in Ensemble 1 suggesting that the randomized precision had a very small effect. When the randomization of precision was carried out within the range of 4 to 10 digits beyond decimal points we found 93.0% overlap of the feasible vectors with Ensemble 1, whereas the 6-10 range resulted in 93.4% overlap. In other words, the results were consistent with different

settings for precision randomization. We would also like to point out that ODE solver packages used in the literature typically come in single and double precision versions [8,9], hence a specific precision setting is to be preferred over randomized precision.

8. The impact of additional normalization on the contributions of individual parameters to the feasible region's volume

To test whether the parameters with small values are dominated by the parameters with large values when the feasible region's volume is computed, we took the following steps: In a DE run using Scheme 7 (with expansion of the feasible region), we computed the contributions of each model parameter to the volume of the feasible region in each generation (for a total of 2200 generations) of DE from the ensemble generated by the 19 parent vectors. Next, we computed the mean value of each parameter among all parent vectors and the mean value of the parameter's contribution to the feasible region volume (log10 normalized as in equation 4). The Pearson correlation coefficient between the two vectors (mean values and mean contributions) was 0.0074. The lack of correlation indicated that log10 normalization did a good job in terms of preventing parameters with small values from getting dominated by the parameters with large values while computing the feasible region's volume.

Next, we computed the contributions of each parameter to the feasible region's volume and ranked them based on their magnitude (one ranking per parameter per generation) which indicated fairly consistent ranking of the parameters among different generations. Pearson correlation coefficients between vectors formed by the parameter rankings in different generations ranged between 0.9980 to 1 (based on a 2200x2200 correlation matrix). Then, we identified the ratio of each parameter's contribution to the lowest contributing parameter. Inspection of these ratios led to the following observations: The ratios were within a 4-fold range 66.5% of the time on average (65.10%-67.11% range in 2200 generations), whereas they were within a 10-fold range 90.4% of the time (89.30%-91.30% range in 2200 generations). Therefore, we implemented another normalization (in addition to using log10 transformation) by computing a normalization coefficient as the ratio between the multiplicative inverse of the contribution per parameter and the sum of this value for all parameters. We multiplied the contribution per parameter (log10 of the difference between maximum and minimum values of the parameter) with this normalization coefficient while computing the feasible region's volume throughout an additional DE run. First, we confirmed that the contributions of individual parameters to the feasible region's volume were equal to each other (per volume computed in each generation). We then assessed the effects of this normalization approach on our analysis. We performed four individual DE runs

(2200 generations total) and obtained a new set of feasible vectors (Ensemble 17).

- Ensemble 17 (16176 feasible vectors) had a size (number of feasible vectors) that is only 4% higher than the size of Ensemble 15 generated under the same conditions (without volume normalization).

- The number of consistently viable mutants (among the 129 novel mutants) was 89 in Ensemble 17, which was equal to the same number on Ensemble 15.

- Prediction range of Ensemble 17 was 301 (the number of unique prediction vectors), only 1% higher than the prediction range of Ensemble 15 (293).

- For both ensembles 15 and 17, we ranked the informativeness of the 90 consistently viable novel mutants based on a prediction variability statistic, namely the sum of the values in each mutant's distribution of relative abundance CV's. The correlation between the ranks of these 90 mutants among the two ensembles was 0.9852 indicating the consistent ranking of the mutants with and without the volume normalization.

- For both ensembles 15 and 17, we ranked the informativeness of the 26 model species based on the sum of the CV values of relative abundances that included each species. The correlation between the ranks of these 26 species among the two ensembles was 0.9788 indicating the consistent ranking of the mutants with and without the volume normalization.

These results confirm that the additional normalization, which equalized the contributions of individual parameters to the feasible region's volume, did not significantly affect the results of our analysis.

9. The impact of additional normalization on the contributions of individual parameters to the robustness score

We also checked if the parameters with small absolute values will be dominated by those with large absolute values while computing the robustness score. As a reminder, the 10 most critical parameters in Table 3 are perturbed with different magnitudes to compute this score.

First, we computed the mean value of each of these 10 parameters in Ensemble 16 (the ensemble with the widest prediction range):

[2.06 0.14 0.39 3.03 1.25 3.37 0.27 0.47 0.99 0.70].

Next, we computed the contribution of each parameter to the robustness scores (one score for each of the 15050 feasible vectors in the ensemble) and obtained one value per parameter by summing its contributions to all of the 15050 individual scores:

[641650 701095 712215 797176 797283 776966 722972 613693 783316 621420].

Finally, we computed the Pearson correlation coefficient of these two vectors as 0.45 (p-value=0.19).

Since there was not a significant correlation, we concluded that parameters with large absolute values do not dominate the robustness score computations.

10. The impact of the viability criteria on the model prediction range

We note that the viability criterion for any given cell cycle mutant is as follows: "if cell size at the last division is within 5% of the size values in the previous two divisions, then the mutant is considered viable; if this difference is greater than 5%, but cell size is still less than 25, the mutant is considered neither viable nor inviable".

The size thresholds (absolute size and its variability) used to decide viability are based on the experimentally measured sizes of a large number of single budding yeast cells (and the size variability up to 20%) at birth in [10]. In order to assess the sensitivity of our results with respect to the range of cell size in the last three divisions (Vdiv) and maximum cell size (Vmax) in simulations, we used different thresholds for deciding cell viability. We note that the period (or frequency) of the cell cycle is fairly constant for any given growth rate (for viable cells that continually divide), hence only the cell's size is used to detect viable cycles as previously done in earlier cell cycle models, including the Chen2004 model [11]. For Vdiv, other than the standard 5% value (for the acceptable range of the cell size in the last three divisions), we used 10%, 15%, and 20% values. On the other hand, for Vmax, other than the standard maximum allowable cell size of 25, we used values of 50, 75, and 100. With standard values of Vmax and Vdiv (cell size of 25 and 5% range of size at division),the prediction range (number of unique prediction vectors for novel cell cycle mutants) was identified as 195 from the first of the four runs that generated Ensemble 16 using Scheme 8 that is described in Table 2. With varying maximum allowed cell size, the prediction range values were 195, 198, and 198 (0%-1.5% variability with respect to the reference value of 195) for the three maximum cell size thresholds of 50, 75, and 100. When we varied the allowed range of cell size at the last three divisions, the prediction range values were 180, 155, and 135 (8%-30% variability with respect to the reference value of 195) for the three ranges of acceptable cell size ranges of 10%, 15%, and 20%. These results suggested that the prediction is fairly sensitive to the the acceptable range of the cell size in the last three divisions, whereas there is no sensitivity compared to the maximum allowable cell size in a simulation.

The considerable sensitivity of the prediction range to Vdiv is due to the model and its initial parametrization rather than the parameter space exploration scheme. Any approach taken to explore the parameter space for feasible parameter vectors requires prior establishment of feasibility criteria. We selected the base values of Vdiv and Vmax (5% and 25) during the model development stage, based on the experimental

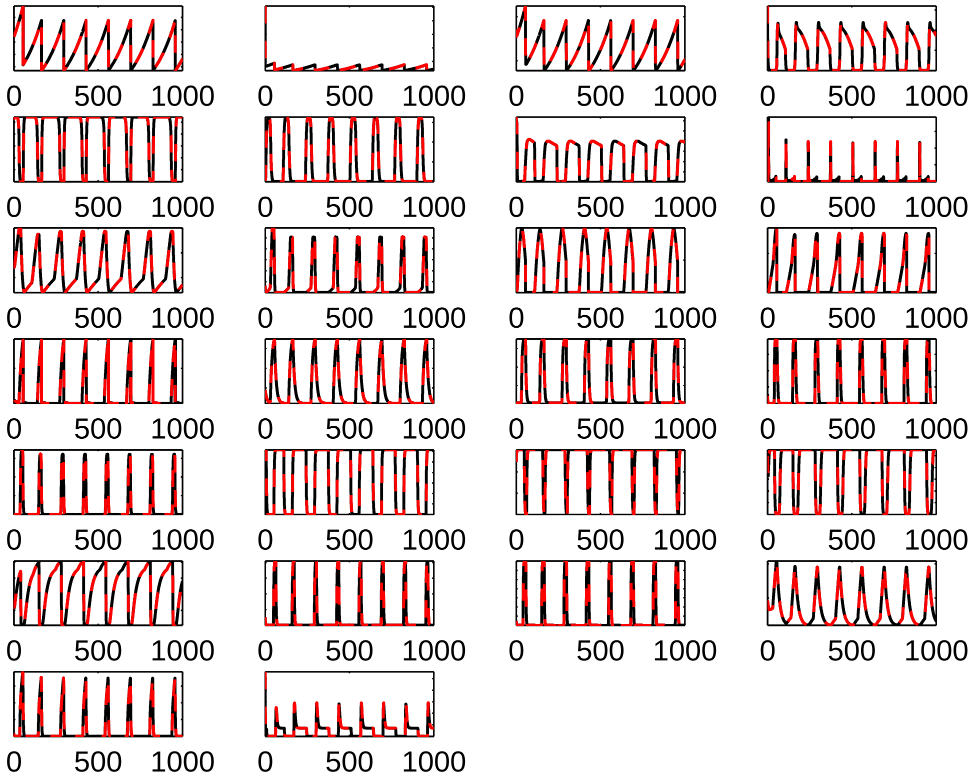

Figure 1: The comparison of wild type trajectories of the 26 model variables with LSODAR (black lines) and Euler's method (red lines). Time and concentrations of variables (cell cycle regulators) are represented by the x-axis and y-axis, respectively. LSODAR based results are obtained with the relative tolerance and absolute tolerance values of 1E-12, whereas the step size for Euler's method is 0.05 min.

data indicating that viable cells never grow more than ten times larger than wild-type cells and that the variability of Vdiv is typically 10–15% [10]. (In a deterministic model of cell cycle progression, the variability of Vdiv should be considerably smaller than the observed variability). If one desires to take into account the observed sensitivity of the prediction range to Vdiv, one could allow a range of Vdiv values (e.g., 5%-20%) for viable cell cycles while looking for feasible points in the parameter space.

11. The choice of ODE solver

In addition to the Euler-based ODE solver, we also used a higher order ODE solver (LSODAR) while this model was being developed. The initially published results of this model were actually based on LSODAR [12]. The results reported in this paper are based on Euler's method with a fixed step size in order to stay consistent with our previous study [13]. In addition, since Euler's method handles both deterministic and stochastic cases, it allows direct comparison between the deterministic model (used in this study) and

its stochastic version [14]. Figure 1 in this Supplementary Text compares the trajectories of the 26 model variables generated by LSODAR (black lines) and Euler's method (red lines) in a simulation of wild-type cells. The trajectory pairs are almost identical for all model variables indicating that the results are not dependent on the choice of the ODE solver. LSODAR based results are obtained with the relative tolerance and absolute tolerance values of 1E-12, whereas the step size for Euler's method is 0.05 min.

In a recently published study [4], we compared global optimization methods (including DE) on this cell cycle model, simulated by LSODAR. We found several optimal parameter vectors that correctly simulated the phenotypes of 110–112 of the strains listed in Table S6 suggesting that LSODAR and Euler's method give similar results for all yeast cell strains in our Training Set. Regarding its stability, LSODAR uses Adams methods (predictor-corrector) in the nonstiff case, and Backward Differentiation Formula (BDF) methods in the stiff case by checking for stiffness at every step, and both are implicit stable methods, whereas the step size we used for Euler's method (0.05 min) is set to ensure stability and to achieve an agreement of the individual trajectories of model variables with LSODAR.

12. Biological application

One potential application of this work is the identification of specific mutations that significantly impact the variability of relative protein abundances. Such variability is a critical aspect in biological systems, as indicated by the impact of disease-causing mutations in the human body through their effects on protein abundances even when these mutations are synonymous [15]. It has also been reported recently that proteins harboring germline mutations have different abundance variability signatures compared to proteins with somatic mutations [16], once again highlighting the intimate link between protein abundances and disease states.

The most informative relative abundance we identified is the ratio of APCP to Cdc20A-APC, which has a CV value of 0.53 in Mutant 128 (Table S5). This triple mutant is created by the elimination of three reactions in our model, namely SBF phosphorylation by Clb2, Whi5 dephosphorylation by Cdc14, and Net1 dephosphorylation by PPX. (For information regarding the mapping and analysis of phosphorylation sites that is critical for gaining a mechanistic understanding of signaling networks, we refer to reader to a review paper [17] and experimental studies that implemented mutations on phosphorylation sites of cell cycle proteins [18, 19]). If either one of the dephosphorylation mutations is dropped, keeping the mutation in the other dephosphorylation reaction and in the phosphorylation of SBF by Clb2 (Mutants 41 and 42 in Table S5), there is very little change in the CV of APCP/Cdc20A-APC ratio. However, if the mutation in

SBF phosphorylation by Clb2 is dropped and the two dephosphorylation mutations are kept (Mutant 45), the CV value of APCP/Cdc20A-APC drops to 0.12 (a 77% drop). Such model predictions can be tested in drug design studies, especially when a single protein or a pair of proteins are known to be involved in a disease state, since predictions of this nature point to individual mutations that are strongly (or weakly) tied to the variabilities of specific protein abundances.

13. Most critical model parameters

Close to the top of the list of ten most critical parameters (Table 3) are the total amounts of Cdc14 and Net1, because these two proteins bind to each other to prevent cells from exiting mitosis ahead of schedule. If there is too much Cdc14 or too little Net1, then Cdc14 will be released from the Net1:Cdc14 complex (called RENT) ahead of schedule, and cells will die of a mitotic catastrophe. The criticality of this interaction is confirmed by two other parameters on the list in Table 3, namely #7 (the rate constant for formation of the RENT complex) and #9 (the rate constant for phosphorylation of Net1 by Clb2, which leads to the release of Cdc14 from the RENT complex at the metaphase-to-anaphase transition). All four of these parameters are critically associated with the timing of exit from mitosis, and perturbations of these parameters can easily convert a viable strain to an inviable one or vice versa. Also on the list are two other parameters related to the metaphase-to-anaphase transition: #3 (the total amount of Esp1) and #6 (the rate constant for Esp1 inactivation by PPX). Esp1 (also known as securin) binds to and inactivates Pds1 (also known as separase). After the replicated chromosomes are fully aligned on the metaphase plate (SPN = 1), PPX inactivates Esp1, releasing Pds1, which promotes the separation of sister chromatids during anaphase. Again, it is clear that perturbations of this delicately balanced set of interactions can easily convert a viable strain to an inviable one or vice versa. This conclusion is confirmed by the appearance of the "rate constant for SPN accumulation" at position #2 in Table 3. If SPN accumulates too slowly or too rapidly in prometaphase, then the metaphase-to-anaphase transition may easily be compromised. Another critical parameter (#10 on the list) is the total amount of Mcm1, which is the transcription factor for the synthesis of Clb2, Swi5, Cdc20 and Polo kinase, proteins that all play important roles in mitotic exit. The degradation rate of Cdc20 is also on the list (#5), underscoring the critical role of the total cellular content of Cdc20 (a balance between Cdc20 synthesis and degradation) in the EXIT module. Finally, #8 on the list is the rate constant for the activation of nine of the proteins in Table S2. The larger its value, the faster these nine proteins (about one third of the model variables) are activated. It is not surprising, then, that perturbations of this time scale may have profound effects on the viability or inviability of many mutant strains.

14. Most fragile phenotypes

We list the ten most fragile phenotypes (mutants) in Table 4. In all cases the viability or inviability of the mutant strain is a consequence of a subtle balance among opposing interactions and is, therefore, sensitive to parameter variations in the underlying deterministic model. Hence, the phenotype prediction is fragile according to our sensitivity analysis. For example, cells carrying the Clb2 destruction box deletion *CLB2-db*Δ produce excess Clb2 protein in mitosis, because the mutant protein is degraded at a slower rate. The excess of Clb2 blocks these mutant cells in mitosis, so the mutant strain is inviable. *CLB2-db*Δ cells can be rescued by introducing multiple copies of *SIC1* (mutant 61), because the Sic1 protein inhibits excess Clb2, or by deleting the genes encoding Clb5 and Clb6 (the double deletion in mutant 63). The viability of mutants 61 and 63 is sensitive to other parameters in the model, making the phenotype predictions of these mutant fragile. On the other hand, the inviable mutants, #59 *CLB2-db*Δ in slow-growth (galactose) medium and #56 *GAL-CLB2 cdh1*Δ, are also close to the viability-inviability transition in deterministic models, and hence they are sensitive to parameter variations, making the phenotype predictions of these mutants fragile as well. The fragility of mutants 59 and 56 (the only two inviable mutants among the ten most fragile) is attributable to the strong global influence of Clb2 on the cell cycle network. In particular, high levels of Clb2 in mitosis, as is the case in *CLB2-db*Δ and *GAL-CLB2* strains, can cause cells to arrest in mitosis. Whether they arrest or not (i.e., inviable or viable, respectively) depends on the level of other B-type cyclins (Clb5 and Clb6) and the activities of the antagonists of the B-type cyclins (Cdh1 and Sic1). Simultaneous deletion of the two G1 cyclins *cln1*Δ *cln2*Δ, which promote production of B-type cyclins, and the protein responsible for B-type cyclin degradation in G1 *cdh1*Δ balance each other to keep the cells viable (mutant 18). But the balance is apparently sensitive to other parameter values, because the viability of this mutant strain is fragile. Its fragility is maintained even if Cln2 protein is overexpressed in the *cln1*Δ *cln2*Δ *cdh1*Δ *GAL-CLN2* strain grown in galactose (slow growth conditions) (mutant 20). APC phosphorylation (activation) by Clb2 is set to zero in *APC-A* (mutant 77) and this inhibits the degradation of Clb5 and Clb2. Despite the delayed separation of sister chromatids and degradation of Clb2, *APC-A* cells are viable since Cdc20A-APC eventually degrades cyclins allowing the cells exit from mitosis, even with reduced levels of the CDK inhibitor (Sic1) in mutant 78 (*APC-A sic1*Δ). Another interesting combination of exit module mutations that lead to viability is seen with mutant 105 (*cdc15*Δ *net1-ts cdh1*Δ). In this case, the inviable strain *cdc15*Δ (no Cdc15 → less phosphorylation of Net1 → higher levels of active Net1 → lower levels of active Cdc14 → inability to exit from mitosis) is rescued by a lowered association rate between Net1 and Cdc14 *net1-ts* (hence there is sufficient Cdc14 activity in this mutant to permit exit from mitosis, even in the

absence of Cdh1). Finally, cells carrying the Clb5 destruction box deletion *CLB5-db*Δ are viable with and without the additional deletion of "securin" *pds1*Δ (mutant 73), a protein involved in chromatid separation. In all these cases, the mutant cells are close to the transition between viability and inviability (in the context of the deterministic model), and hence there predicted phenotype is sensitive to parameter variations.

References

1. Santner TJ, Williams B, Notz W: *The Design and Analysis of Computer Experiments*. Springer-Verlag 2003.
2. Dayarian A, Chaves M, Sontag ED, Sengupta AM: **Shape, size, and robustness: feasible regions in the parameter space of biochemical networks**. *PLoS Comput Biol* 2009, **5**:e1000256.
3. Hwang CR: **Simulated annealing: theory and applications**. *Acta Applicandae Mathematicae* 1988, **12**:108–111.
4. Andrew T, Amos B, Easterling D, Oguz C, Baumann W, Tyson J, Watson L: **Global parameter estimation for a eukaryotic cell cycle model in systems biology**. In *Proceedings of the 2014 Summer Simulation Multiconference*, Society for Computer Simulation International 2014:45.
5. Cho K, Shin S, Kolch W, Wolkenhauer O: **Experimental design in systems biology, based on parameter sensitivity analysis using a Monte Carlo method: A case study for the TNF alpha-mediated NF-kappa B signal transduction pathway**. *Simul-T Soc Mod Sim* 2003, **79**(12):726–739.
6. Wolpert DH, Macready WG: **No free lunch theorems for optimization**. *Evolutionary Computation, IEEE Transactions on* 1997, **1**:67–82.
7. Ho YC, Pepyne DL: **Simple explanation of the no-free-lunch theorem and its implications**. *Journal of optimization theory and applications* 2002, **115**(3):549–570.
8. Cohen SD, Hindmarsh AC: **CVODE, a stiff/nonstiff ODE solver in C**. *Computers in physics* 1996, **10**(2):138–143.
9. Hindmarsh AC: **Serial Fortran solvers for ODE initial value problems**. *URL: http://www. llnl. gov/CASC/odepack [cited October 18, 2005]* 2002.
10. Di Talia S, Skotheim JM, Bean JM, Siggia ED, Cross FR: **The effects of molecular noise and size control on variability in the budding yeast cell cycle**. *Nature* 2007, **448**(7156):947–951.
11. Chen KC, Calzone L, Csikasz-Nagy A, Cross FR, Novak B, Tyson JJ: **Integrative analysis of cell cycle control in budding yeast.** *Mol Biol Cell* 2004, **15**(8):3841–3862.
12. Laomettachit T: **Mathematical modeling approaches for dynamical analysis of protein regulatory networks with applications to the budding yeast cell cycle and the circadian rhythm in cyanobacteria**. *PhD thesis*, Virginia Institute of Technology 2011, [http://scholar.lib.vt.edu/theses/available/etd-11072011-021528/].
13. Oguz C, Laomettachit T, Chen KC, Watson LT, Baumann WT, Tyson JJ: **Optimization and model reduction in the high dimensional parameter space of a budding yeast cell cycle model**. *BMC systems biology* 2013, **7**:53.
14. Oguz C, Palmisano A, Laomettachit T, Watson LT, Baumann WT, Tyson JJ: **A stochastic model correctly predicts changes in budding yeast cell cycle dynamics upon periodic expression of CLN2**. *PloS one* 2014, **9**(5):e96726.
15. Sauna ZE, Kimchi-Sarfaty C: **Understanding the contribution of synonymous mutations to human disease**. *Nature Reviews Genetics* 2011, **12**(10):683–691.
16. Schaefer MH, Yang JS, Serrano L, Kiel C: **Protein conservation and variation suggest mechanisms of cell type-specific modulation of signaling pathways**. *PLoS Comput Biol* 2014, **10**(6):e1003659.
17. Dephoure N, Gould KL, Gygi SP, Kellogg DR: **Mapping and analysis of phosphorylation sites: a quick guide for cell biologists**. *Molecular biology of the cell* 2013, **24**(5):535–542.

18. Herbig U, Griffith JW, Fanning E: **Mutation of cyclin/cdk phosphorylation sites in HsCdc6 disrupts a late step in initiation of DNA replication in human cells**. *Molecular biology of the cell* 2000, **11**(12):4117–4130.

19. Russo GL, van den Bos C, Marshak DR: **Mutation at the CK2 phosphorylation site on Cdc28 affects kinase activity and cell size in Saccharomyces cerevisiae**. *Molecular and cellular biochemistry* 2001, **227**(1-2):113–117.
