# Supplementary material for: Predicting network modules of cell cycle regulators using relative protein abundance statistics
Source: BMC Syst Biol. 2017 Feb 28;11:30. doi: 10.1186/s12918-017-0409-1 (PMC5329933; doi:10.1186/s12918-017-0409-1)
Supplement: Additional file 3 — Supplementary Figures. This pdf file includes five additional figures. (PDF 1640 kb) [file 12918_2017_409_MOESM3_ESM.pdf]

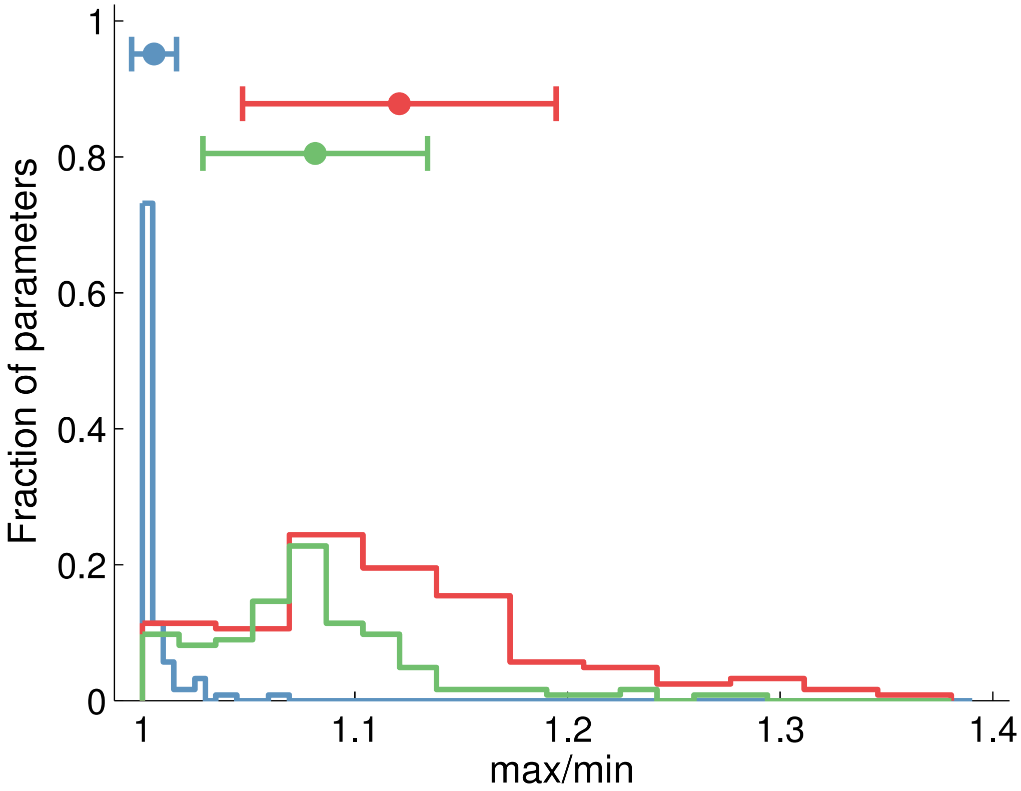

Figure S1. Distributions of the parameter range magnitudes for three distinct feasible parameter vector populations. Mean ± standard deviation for each distribution (listed in Table S9) is depicted by a single horizontal bar. The range of each parameter (x-axis) is computed as the ratio of its maximum and minimum values among each parameter vector population. Blue/green/red line: Magnitudes of the parameter ranges covered by all the parent vectors at the $400^{th}/400^{th}/1600^{th}$ generation of DE with Scheme 3/4/4 used for exploring the parameter space. Details of each scheme (1–8) are given in Table 2.

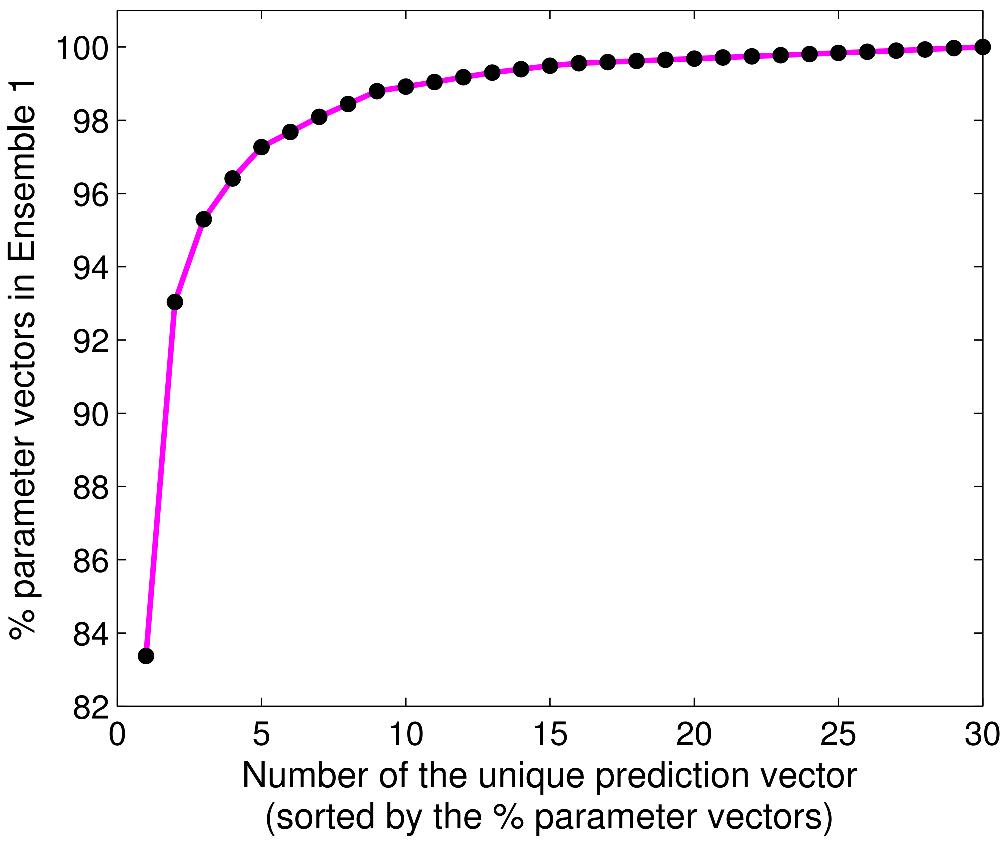

Figure S2. Percent of parameter vectors in Ensemble 1 that generate each unique prediction vector. This figure illustrates that 97% of the feasible parameter vectors in Ensemble 1 generate only five of the total of 30 unique prediction vectors.

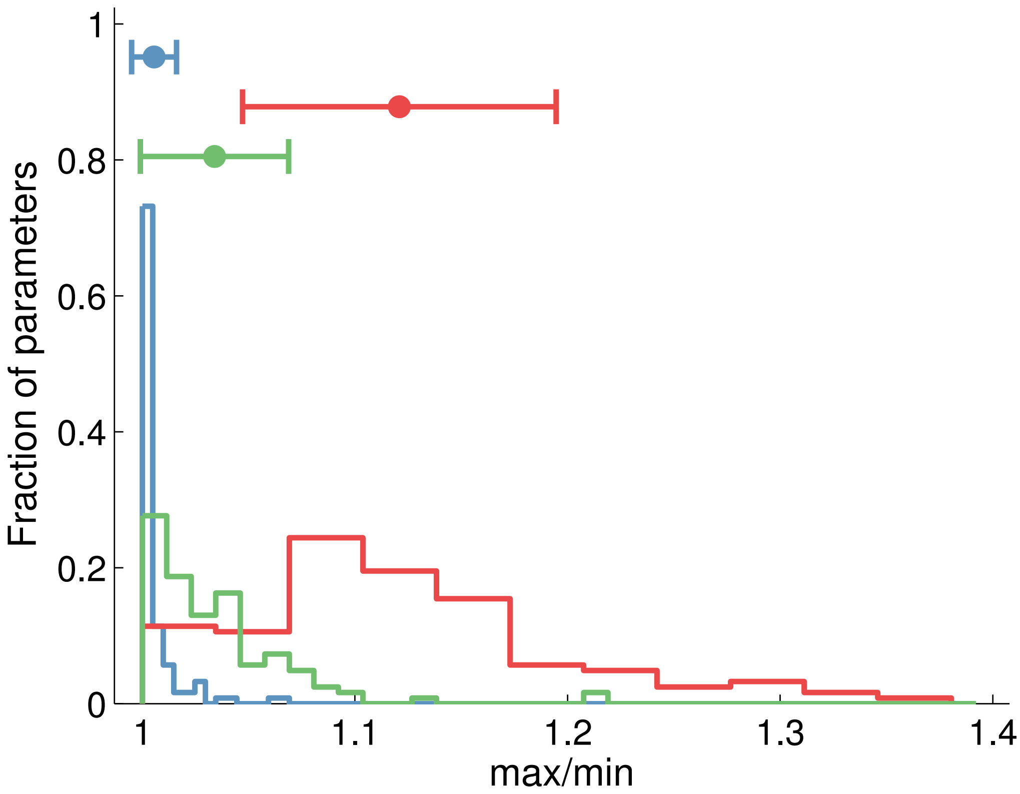

Figure S3. Distributions of the parameter range magnitudes for three distinct feasible parameter vector populations. Mean ± standard deviation for each distribution (listed in Table S10) is depicted by a single horizontal bar. The range of each parameter (x-axis) is computed as the ratio of its maximum and minimum values among each parameter vector population. Blue/green/red line: Magnitudes of the parameter ranges covered by all the parent vectors at the $400^{th}/1600^{th}/1600^{th}$ generation of DE with Scheme 2/8/4 used for exploring the parameter space. Details of each scheme (1–8) are given in Table 2.

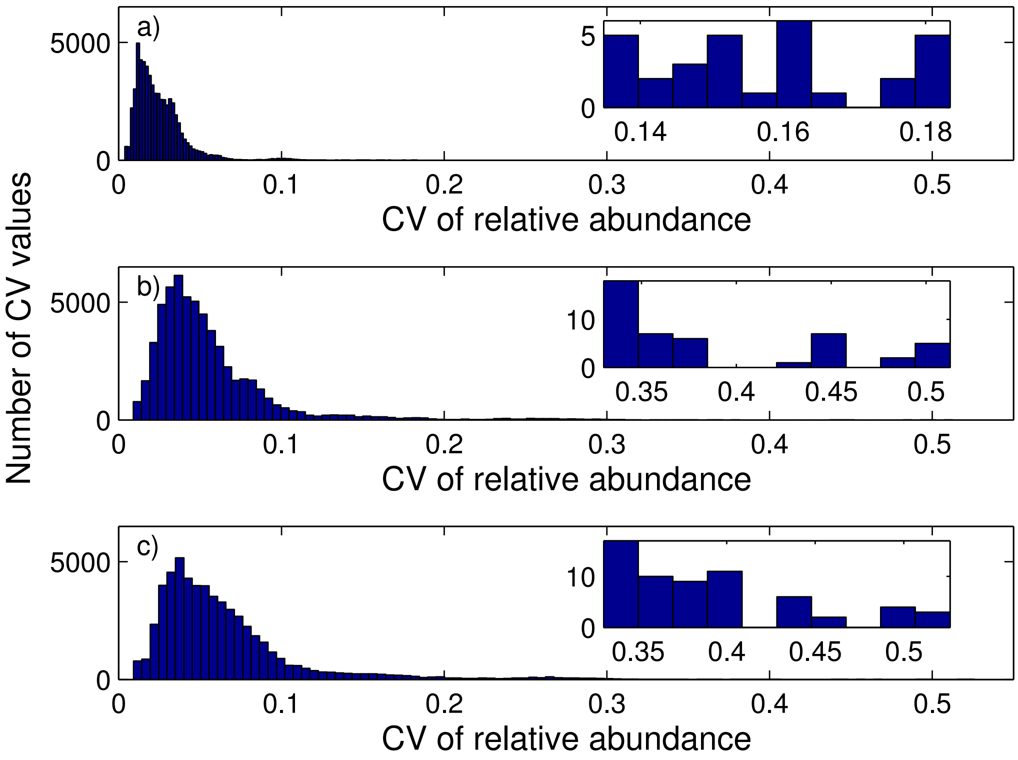

Figure S4. CV (standard deviation normalized by the mean) values of the relative abundance predictions generated by: a) Ensemble 1, b) Ensemble 15, and c) Ensemble 16. Insets are used to depict the maximum values within the distributions.

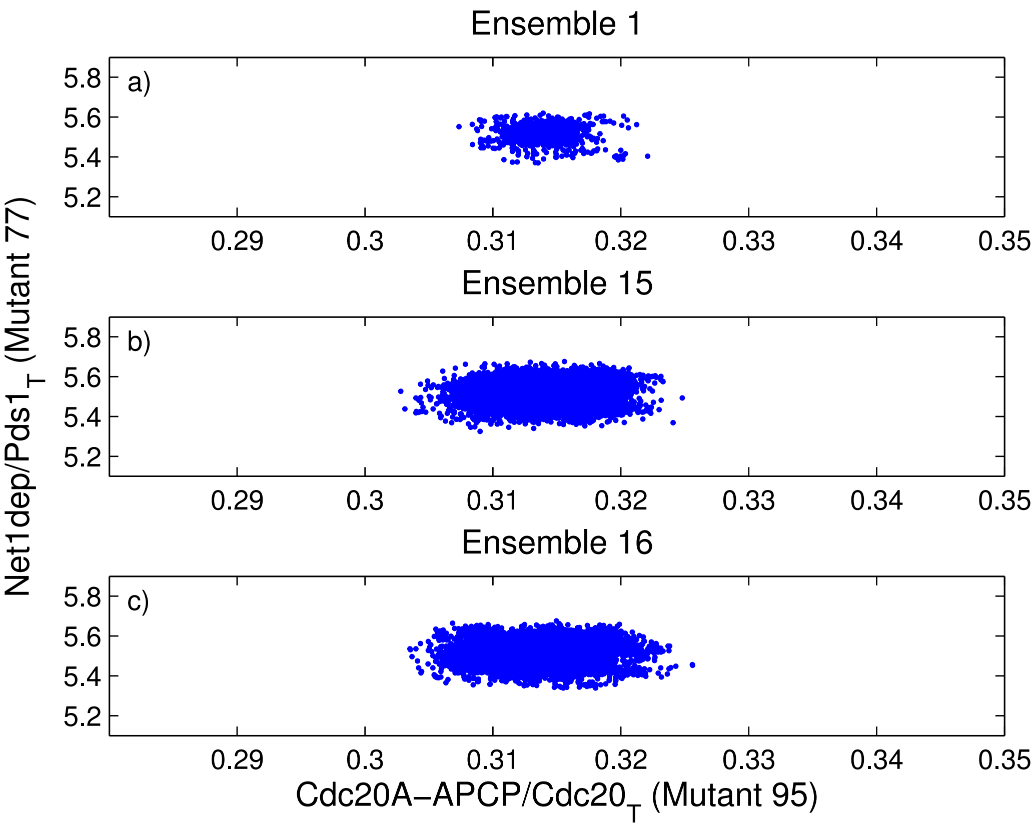

Figure S5. The comparison of relative abundance predictions that are generated from three different ensembles of parameter vectors. These relative abundance predictions (with low variability) are generated by Ensembles 1 (in a), 15 (in b), and 16 (in c). CV values of these predictions are of 0.0041/0.0093/0.0090 (x-axis) and 0.0047/0.0098/0.0095 (y-axis) among Ensemble 1/15/16.
